# Spatial transcriptomics atlas of inflammatory bowel disease to guide implementation in research consortiums and clinical trials

**DOI:** 10.1038/s41467-026-72482-w

**Published:** 2026-04-28

**Authors:** Yiming Li, Cenfu Wei, Wenjing Yang, Hai Wang, Alan Robinson, Isabella Peshek, Tianming Yu, Jiwoon Park, Jenny Yanyi Ding, Stephen B. Hanauer, Emanuelle Bellaguarda, Laura Yun, Ronen Sumagin, Guang-Yu Yang, Christopher E. Mason, James D. Lewis, Deyu Fang, Yingzi Cong, Yuan Luo, Parambir S. Dulai

**Affiliations:** 1 Department of Preventive Medicine, Northwestern University, Chicago, IL USA; 2 Department of Medicine, Division of Gastroenterology and Hepatology, Northwestern University, Chicago, IL USA; 3 Department of Systems and Computational Biomedicine, Weill Cornell Medicine, New York, NY USA; 4 The HRH Prince Alwaleed Bin Talal Bin Abdulaziz Alsaud Institute for Computational Biomedicine, Weill Cornell Medicine, New York, NY USA; 5 Department of Pathology, Northwestern University, Chicago, IL USA; 6 The Feil Family Brain and Mind Research Institute, Weill Cornell Medicine, New York, NY USA; 7 Division of Gastroenterology and Hepatology, Perelman School of Medicine, University of Pennsylvania, Philadelphia, PA USA; 8 Center for Human Immunobiology, Northwestern University, Chicago, IL USA; 9 Department of Microbiology and Immunology, Northwestern University, Chicago, IL USA; 10 Northwestern University Clinical and Translational Sciences Institute, Northwestern University, Chicago, IL USA; 11 Center for Collaborative AI in Healthcare, Institute for AI in Medicine, Northwestern University, Chicago, IL USA

**Keywords:** Immunology, Computational biology and bioinformatics, Gastroenterology, Biological techniques

## Abstract

Using over 100 intestinal tissue sections from non-diseased controls and patients with ulcerative colitis or Crohn’s disease across multiple inflammatory bowel disease consortia, we construct a spatially resolved atlas containing over three million cells and systematically evaluate two imaging-based spatial transcriptomics platforms. Here we show that CosMx tends to achieve higher detection efficiency than Xenium across commercially available panels in both ulcerative colitis and Crohn’s disease, whereas Xenium shows reduced performance associated with tissue type, block quality, and panel size. CosMx identifies regulatory T cell associated biology in both disease subtypes, which we validate using independent laboratory experiments and multi-plex spatial multi-omics. CosMx’s data quality remains stable across variation in fixation time and sectioning procedures, supporting its operational feasibility for multi-center studies. This study establishes a technical and biological benchmark for the application of single-cell-resolved spatial transcriptomics in gastrointestinal tissue, enabling more reliable application of spatial technologies in translational inflammatory bowel disease research.

## Abstract (web summary)

A spatial transcriptomic atlas of tissue from healthy people and people with inflammatory bowel disease evaluates spatial transcriptomics platforms, reveals Treg biology, and establishes a benchmark for applying spatial transcriptomics in translational research.

## Introduction

Inflammatory bowel diseases (IBD), which encompasses both ulcerative colitis (UC) and Crohn’s disease (CD), are immune-mediated inflammatory disorders of the gastrointestinal tract^1^. Within the intestinal microenvironment, niche signaling and cell-cell interactions are instrumental in driving or resolving inflammation in IBD^2^. Prior work has demonstrated the value of characterizing aggregated (bulk RNA-seq) and dissociated single-cell (scRNA-seq) transcriptomic signatures to identify associations with disease development, progression, and/or resolution^3–18^. However, these methods lack spatial context (e.g., lymphoid aggregates, crypt structure), which is essential for understanding the organization and interactions of cells within tissue.

Recent advances in sequencing-based and imaging-based spatial transcriptomics platforms enable single-cell and/or subcellular resolution profiling while preserving spatial information. Application of imaging-based technologies to formalin-fixed paraffin-embedded (FFPE) tissue offers a unique opportunity for using archived tissues, alignment with histologic features of disease, and improved feasibility for implementation of single-cell profiling within large research consortiums or clinical trials. Comparative studies to date for imaging-based spatial platforms in FFPE tissues have identified technical differences among the available platforms, with most comparisons being between Xenium and CosMx^19–25^. These early studies have been informative in understanding how spatial platforms can be qualitatively and quantitatively compared, but several limitations are present, including the relative size of studies for sample and/or slide comparisons, limited sampling of tissue specimens through tissue microarrays which fail to capture the breadth of spatial diversity and cellular architecture, technical differences in sample preparation or custom panel creation for platforms, and/or lack of consideration for segmentation variation across platforms and over time. Furthermore, none of these comparative studies have explored how differential performance of platforms would influence biological discovery within clinical cohort studies.

A comprehensive, sufficiently powered, systematic benchmarking study of these high-plex spatial transcriptomics platforms is needed to accurately guide the field of IBD on use of these technologies for drug development, biomarker discovery, and scientific innovation. In this study, we generate a large-scale spatial atlas of IBD and use it to systematically compare CosMx and Xenium in IBD across diverse tissue states, qualities, and panel designs for both platforms. We observed enhanced detection efficiency for CosMx compared to Xenium and enhanced feasibility for the implementation of CosMx in multi-center collaborations. This framework enables a clearer understanding of how platform characteristics shape data quality and biological interpretation, providing a foundation for reliable and reproducible spatial profiling in multi-center IBD studies.

## Results

### CosMx multi-tissue panel versus Xenium multi-tissue panel

The CosMx multi-tissue panel (950 genes) and the Xenium multi-tissue panel (377 genes) were run on 16 IBD intestinal FFPE blocks (*n* = 32 mucosal biopsies) and comparisons were made for the overall panels and overlapping genes (119 genes; Supplementary Dataset 1). All runs were performed for both platforms from serial sections cut by an experienced technician involved in clinical cohort studies and clinical trials, with oversight from both independent labs performing experiments. Runs were done independently at the same time to ensure consistency in instrument and software versions available for both technologies. CosMx and Xenium runs all passed quality metrics recommended by each respective company, and all Xenium runs were observed to have high overall probe detection confidence, achieving tissue-level Q-scores of greater than 20, consistent with vendor (10× Genomics) recommendations. (Supplementary Dataset 2)

**Fig. 1:**
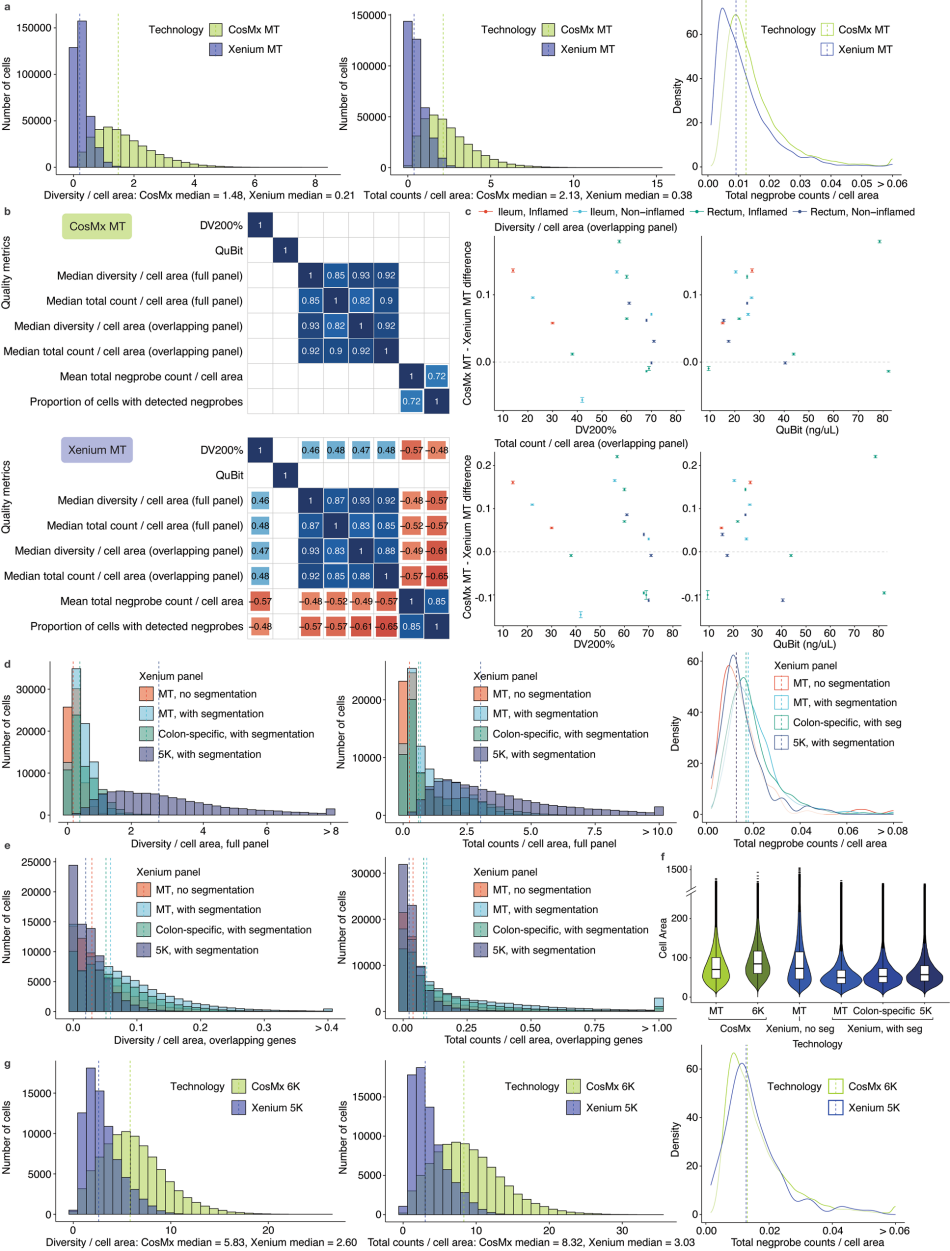
Comparative evaluation of CosMx and Xenium data in inflammatory bowel disease. **a** Transcript diversity, total counts, and total negprobe counts in CosMx multi-tissue data (*n* = 324,449 cells) and Xenium multi-tissue data (*n* = 369,331 cells). Medians are shown as vertical dashed lines, and total negprobe counts are visualized using density plots. **b** Kendall rank correlations among DV200%, QuBit, and data quality metrics in CosMx multi-tissue and Xenium multi-tissue data (*n* = 16 FFPE blocks, 32 biopsies each). Significant correlations (adjusted *p*-value < 0.05) are shown; tiles are colored by correlation coefficients. **c** Median data quality differences between CosMx multi-tissue and Xenium multi-tissue datasets (*n* = 16 FFPE blocks, 32 biopsies). The 95% confidence intervals from two-sided Wilcoxon rank sum tests are shown, and data points are colored by sample category. **d** Transcript diversity, total counts, and total negprobe counts (full panel) as well as **e** transcript diversity and total counts (overlapping panel) in Xenium 5K (*n* = 75,389 cells; 4 FFPE blocks, 8 biopsies), multi-tissue without segmentation (*n* = 66,057 cells; 4 FFPE blocks, 8 biopsies), multi-tissue with segmentation (*n* = 88,978 cells; 4 FFPE blocks, 8 biopsies), and colon-specific data (*n* = 66,338 cells; 4 FFPE blocks, 8 biopsies). Medians are shown as vertical dashed lines, and total negprobe counts are visualized using density plots. **f** Cell area distributions across technologies and panels (CosMx multi-tissue: 324,449 cells, CosMx 6K: 89,058 cells, Xenium multi-tissue without segmentation: 435,388 cells, Xenium multi-tissue with segmentation: 88,978 cells, Xenium colon-specific: 66,338 cells, Xenium 5K: 75,389 cells). Boxplots display medians and quartiles, with whiskers extending to 1.5 times the interquartile range; violin outlines represent kernel probability density. **g** Transcript diversity, total counts, and total negprobe counts in CosMx 6K data (*n* = 89,058 cells; 4 FFPE blocks, 8 biopsies) and Xenium 5K data (*n* = 75,389 cells; 4 FFPE blocks, 8 biopsies). Medians are shown as vertical dashed lines, and total negprobe counts are visualized using density plots. Source data are provided in the Source Data file. FFPE formalin-fixed paraffin-embedded, MT multi-tissue, seg segmentation.

At the time of performing this initial comparison, Xenium panels used an expanding boundary detection model for defining cells, which resulted in significantly higher cell sizes compared to CosMx (Supplementary Fig. 1). Xenium had substantially lower data quality as observed by the high number of cells with very low transcript counts (32.78% of Xenium cells and 0.35% of CosMx cells had fewer than 5 transcripts per cell). To help improve the comparative performance for Xenium, a quality control measure was applied where low-quality cells were filtered from the comparative analysis based on thresholds proportional to the plex size and direct visualization of data distribution for each platform (5 transcripts per cell for Xenium, 20 transcripts per cell for CosMx). This resulted in the removal of 32.78% of cells for Xenium and 6.71% of cells for CosMx. After applying this platform appropriate quality filtering and inclusion of only higher-quality cells for Xenium, CosMx retained higher sensitivity for transcript diversity (unique genes) and transcript counts (total counts) for overlapping genes between panels under these matched experimental conditions (Fig. 1a, and Supplementary Dataset 3). The performance difference between CosMx and Xenium was more pronounced when analyzing all the cells without filtering (Supplementary Dataset 4). Both platforms demonstrated intact tissue architecture post-run and feasibility for post-run H&E staining on the same slide to align with spatial data. (Supplementary Fig. 2).

Given the differences in cell segmentation algorithms between CosMx and Xenium, as well as the high proportion of Xenium cells that required removal due to low or undetected transcripts, we evaluated whether re-segmentation could improve Xenium data quality. We applied Baysor, a probabilistic cell segmentation method to enhance segmentation accuracy^26^ previously shown to be the most effective for Xenium data^27,28^. Despite utilizing this alternative best-practice cell segmentation method, Xenium continued to demonstrate weaker performance compared to CosMx (Supplementary Dataset 5).

In this IBD FFPE cohort, CosMx data did not show a detectable association between data quality and block quality. Specifically, there was no correlation between transcript diversity and counts with RNA quality (DV200%) or RNA quantity (QuBit) for FFPE blocks (Fig. 1b). CosMx was observed to have no significant correlation between transcript diversity and counts with negative probes on a per-cell basis. This suggests that background non-specific probe binding, as measured by negative probe detection, has no influence or relationship to data quality with CosMx. This robustness is important given that many IBD research programs rely on FFPE biopsies with variable pre-analytic conditions.

Xenium, however, was observed to have a significant correlation between transcript diversity and counts with RNA quality. The lower the DV200%, the poorer the data quality for Xenium (Fig. 1b). In high-quality FFPE blocks (DV200% > 50%), CosMx still provided better data quality than Xenium (Fig. 1c). Xenium was observed to have a significant correlation between transcript diversity and counts with negative probes on a per-cell basis. The higher the negative probe counts, the lower the transcript diversity and counts for Xenium (Fig. 1b). This suggests that some degree of background correction or filtering based on negative probes may be needed for Xenium data.

### Xenium panel comparisons, and Xenium 5K versus CosMx 6K

With the availability of Xenium protein cell segmentation markers, we wanted to understand if this improved performance, and we wanted to define the performance of different Xenium panels against each other to guide users on panel selection. Adding protein-based segmentation markers might improve boundary accuracy and transcript assignment, thereby potentially increasing sensitivity. Another 4 colon FFPE blocks (UC inflamed and non-inflamed, healthy colon; *n* = 2 biopsies per FFPE block; *n* = 8 total mucosal biopsies) underwent spatial transcriptomics using the 3 commercially available Xenium panels on sequential tissue sections: colon-specific panel with cell segmentation markers (322 genes), multi-tissue panel (377 genes) with or without cell segmentation markers, and 5K panel with cell segmentation markers. The comparison of the multi-tissue panel with and without cell segmentation markers was intended to understand if our comparative assessments may have been influenced by the absence of these markers.

Overall, the 5K panel had higher transcript diversity and counts compared to the other Xenium panels. However, when limiting the comparison to overlapping genes between Xenium panels, the 5K panel was observed to have the lowest per gene sensitivity among all Xenium panels (Fig. 1d, e, Supplementary Fig. 3, and Supplementary Dataset 3). This indicates that the higher total counts in the 5K panel reflect panel size, not improved sensitivity on a per-gene basis. The addition of the cell segmentation markers to the Xenium multi-tissue panel did reduce cell size (Fig. 1f), but it did not have an impact on the sensitivity of the multi-tissue Xenium panel for transcript diversity and counts. This demonstrates that our comparative analysis for CosMx multi-tissue and Xenium multi-tissue panels is unlikely to have been influenced by the built-in Xenium cell segmentation approach.

These same blocks also underwent spatial transcriptomics with the CosMx 6K panel from sequentially cut sections. Like the multi-tissue panel comparison, a filtering step was needed for Xenium 5K due to the large proportion of cells with zero or very low transcript detection (48.81% of Xenium cells and 1.56% of CosMx cells had fewer than 50 transcripts per cell). In the matched colon FFPE experiments, CosMx 6K panel had higher transcript diversity and transcript counts for overlapping genes compared to the Xenium 5K panel despite filtering and inclusion of only high-quality cells for Xenium (Fig. 1g, Supplementary Figs. 3 and 4, and Supplementary Dataset 3). The performance advantage of CosMx 6K was greater when analyzing all the cells and applying no cell filtering (Supplementary Dataset 4, and Supplementary Fig. 5). Re-segmentation of Xenium 5K using Baysor^26^, a method shown to outperform Xenium’s built-in segmentation algorithm^27,28^, reduced some boundary inflation but improvements in transcript assignment were modest and did not meaningfully change comparative performance metrics.

### CosMx identifies Treg-Mast cell interaction in ileal Crohn’s disease

**Fig. 2:**
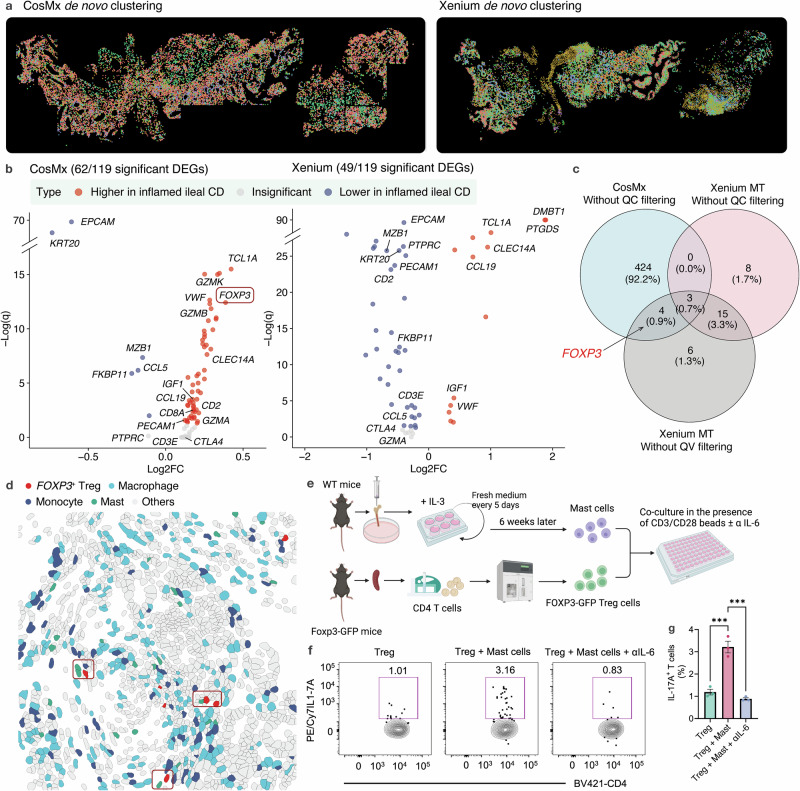
CosMx identifies *FOXP3*^+^ Treg interaction with Mast cells in inflamed ileal Crohn’s disease. **a** De novo clustering results of CosMx multi-tissue and Xenium multi-tissue data from the same sample (I0275 ITDF00A) with a clustering resolution of 0.7. **b** Volcano plot of genes differentially expressed in inflamed ileal CD compared to other cells. FOXP3 is identified as up-regulated inflamed ileal CD in CosMx data (*n* = 34,096 inflamed ileal CD cells vs 290,353 other cells) but not Xenium data (*n* = 24,427 inflamed ileal CD cells vs 344,904 other cells). Visualization is limited to 119 overlapping genes between panels. Known T-cell-related genes and top genes from each technology are labeled. Up-regulated genes are colored red, down-regulated genes blue, and insignificant genes (adjusted *p*-value < 0.05) gray. **c** Venn diagram showing numbers of differentially expressed genes under different filtering criteria: CosMx QC filtering – minimum 20 unique molecular identifiers (UMI) per cell, Xenium QC filtering – minimum 5 UMI per cell, and Xenium QV filtering – minimum Q-score 20 per manufacturer recommendation. Low gene detection confidence as measured by Q-score for Xenium may be the source of the discordance between CosMx and Xenium for differential gene analyses. **d** *FOXP3*^+^ Tregs co-localize with monocytes, macrophages, and mast cells in an ileal inflamed CD sample (I0275 ITDF00A). The areas of co-localization are highlighted. **e** Schematic diagram of the experimental workflow. Created in BioRender. Yang, W. (2026) https://BioRender.com/b30k874. **f** Representative flow cytometry profile of IL-17A^+^ CD4^+^ T cells. **g** Dot plots of the frequency of IL-17^+^ CD4^+^ T cells (*n* = 3 samples per group, three groups in total). Representative data are visualized with confirmation from two independent experiments. One-way ANOVA with Dunnett’s multiple comparisons test was performed (Treg versus Treg + Mast: *p*-value = 0.0003; Treg + Mast versus Treg + Mast + αIL-6: *p*-value = 0.0001). Source data are provided in the Source Data file. ^***^p-value < 0.001; CD, Crohn’s disease; DEG, differentially expressed genes; Log2FC, log fold change; MT, multi-tissue; q, false-discover-rate-adjusted *p*-value; QC, quality control; QV, Xenium Q-score; Treg, regulatory T cell; WT, wild type.

A major objective of our study was to understand if any technical differences between platforms were translated into novel biology. This biological validation is lacking from prior comparative analyzes done to date, but it is needed to ensure accuracy of detection and truly define the meaningfulness of comparative analyzes for spatial platforms. De novo clustering of data from both platforms yielded spatial populations (Fig. 2a, and Supplementary Fig. 6). Differential gene expression (DGE) analysis for inflamed ileal CD demonstrated common biologically relevant genes identified by both platforms (*CCL19*^29^, *IL3RA*^30^, *MZB1*^31^), as well as discordant results between the two platforms. A notable observation was that CosMx identified significant up-regulation of T cell markers in inflamed ileal CD, including FOXP3 a well-established T-reg marker, which were not initially identified by Xenium (Fig. 2b, Supplementary Fig. 7a, and Supplementary Dataset 6). Dysregulation of T cell response is a well-established mechanism for disease pathogenesis in CD, and a recent dissociated scRNA-seq study identified *FOXP3*^+^ (forkhead box P3) pro-inflammatory Tregs in ileal CD, which express IFNg (interferon gamma) and IL17 (interleukin 17)^32^. The observed discordance illustrates how platform-specific sensitivity and probe-level confidence, particularly in inflamed tissue, can impact biological inference. To further explore the source of this discordance between platforms, we examined per-gene probe detection and confidence in Xenium (Supplementary Dataset 6).

As noted in Supplementary Dataset 2, all Xenium multi-tissue panel runs were of high quality, meeting the vendor recommended probe detection confidence threshold (sample-level Q-score > 20). At the per-gene level, 347 out of 377 panel genes also showed Q-scores > 20 across samples, and the 30 genes with lower universal Q-scores were of limited biological relevance to IBD (Supplementary Dataset 7). Notably, although *FOXP3* had a Q-score above 20 aggregating across samples, suggesting high probe detection confidence, per-sample examination revealed reduced *FOXP3* Q-scores specifically in a subset of inflamed ileal CD and UC samples relative to other sample types. It is important to note that Q-scores represent a measure of detection confidence in each tissue after considering background and imaging conditions, not transcript absence. To understand whether this sample-wise variability in probe detection confidence (inflamed versus non-inflamed) influenced cohort-level DGE results, we re-analyzed Xenium data without Q-score filtering and recovered *FOXP3* as a significant differentially expressed gene in inflamed ileal CD (Fig. 2c). This demonstrates that Xenium’s variability in performance confidence, as measured by Q-scores for *FOXP3* across samples, was the underlying reason why *FOXP3* was detected as a significant differentially expressed gene in inflamed ileal CD by CosMx but not by Xenium with standard Q-score filtering.

Spatial cell-cell interaction analyzes of inflamed ileal CD in our CosMx data observed multiple interactions between *FOXP3*^+^ Tregs and other immune cells, including monocytes, macrophages, and mast cells, which were not seen in inflamed rectum CD or inflamed UC samples. Pathways enriched for these ileal CD *FOXP3*^+^ Tregs interactions with monocytes, macrophages, and mast cells included IL-6 (interleukin 6), OX40, IL-1 (interleukin 1), TNF (tumor necrosis factor), and OSM (oncostatin M). Visual inspection of spatial co-localization demonstrated a spatial proximity for *FOXP3*^+^ Tregs with mast cells in inflamed ileal CD (Fig. 2d). Therefore, we sought to confirm that *FOXP3*^+^ Treg interactions with mast cells contributed to the development of pro-inflammatory IL-17-expressing *FOXP3*^+^ Tregs. We also investigated the role of IL-6, a crucial pro-inflammatory cytokine in driving IBD^33^ (Supplementary Methods – Mast cell-Treg experiments).

Splenic Foxp3-GFP^+^ regulatory T cells (Tregs) were isolated from Foxp3-GFP reporter mice and cultured either alone or in co-culture with bone marrow-derived mast cells in the presence or absence of anti-IL-6 antibodies and anti-CD3/CD28 beads. The IL-17A levels in Tregs were assessed by flow cytometry 5 days later. Tregs co-cultured with mast cells exhibited a statistically significantly higher level of IL-17A (3.16%) compared to Tregs cultured alone (1.01%). In addition, treatment of anti-IL-6 significantly reduced IL-17A levels in Tregs during co-culture with mast cells (0.83% vs. 3.16%, Fig. 2e), indicating a possible critical role of the IL-6 produced by mast cells. Additional functional experiments are warranted to confirm the biological importance of this finding. Recent clinical trial evidence demonstrates that anti-IL-6 therapy improves disease activity in CD^34^, and our spatial atlas resource helps to provide biological context to those mechanisms for further exploration.

### CosMx identifies Treg biology in ulcerative colitis

**Fig. 3:**
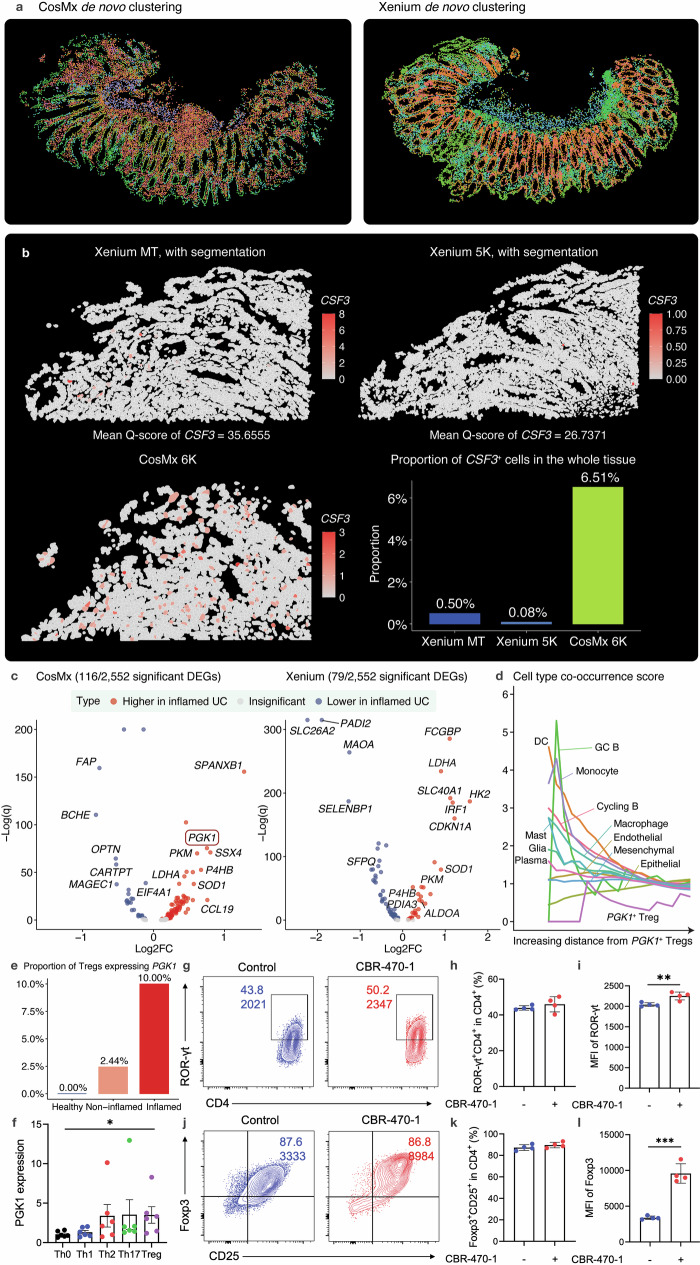
CosMx identifies differential role for *PGK1* in Tregs in inflamed ulcerative colitis. **a**De novo clustering results of CosMx 6K and Xenium 5K data from sample I0387 RSDF00A with a resolution of 0.6. **b** Normalized expression of *CSF3* across CosMx 6K, Xenium multi-tissue, and Xenium 5K data in an inflamed UC sample (I0387 RSDF00A). **c** Volcano plot of genes differentially expressed in inflamed UC and healthy colon. PGK1 is up-regulated in CosMx data (43,347 inflamed versus 23,596 healthy cells) but not Xenium data (28,678 inflamed versus 29,646 healthy cells). Visualization includes 2552 overlapping panel genes. Top genes from each technology are labeled. Up-regulated genes are colored red, down-regulated genes blue, and insignificant genes (adjusted *p*-value < 0.05) gray. **d** Co-occurrence score between *PGK1*^+^ Tregs and other cell types at increasing distances in a CosMx inflamed UC sample (I0387 RSDF00A; 16,627 cells). **e** Proportion of Tregs expressing *PGK1* in healthy (27 Tregs), inflamed UC (130 Tregs), and non-inflamed UC (82 Tregs) samples. **f** PGK1 gene expression in splenic CD4^+^ T cells cultivated under Th0, Th1, Th2, Th17, and iTreg polarization for three days (Treg versus Th0 two-tailed unpaired t test *p*-value = 0.0427). **g**–**i** Splenic CD4^+^ T cells cultivated under Th17 polarization with/without CBR-470-1 (5 μM) for three days. **g** Representative plot and quantification of **h** ROR-γt^+^ CD4^+^ percentage and **i** ROR-γt MFI (p-value = 0.0074). *n* = 4 samples per group, 3 biological replicates per sample. **j**–**l** Splenic CD4^+^ T cells cultivated under iTreg polarization with/without CBR-470-1 (5 μM) for three days. **j** Representative plot and quantification of **k** CD25^+^ Foxp3^+^ percentage and **l** Foxp3 MFI (*p*-value = 0.0001). *n* = 4 samples per group, 3 biological replicates per sample. Data is shown as mean ± standard deviation and analyzed by unpaired two-tailed Student’s *t* test. Source data are provided in the Source Data file. ^*^p-value < 0.05; ^**^p-value < 0.01; ^***^p-value < 0.001; DC, dendritic cells; DEG, differentially expressed genes; GC, germinal center; Log2FC, log fold change; MFI, mean fluorescent intensity; MT, multi-tissue; q, false-discover-rate-adjusted *p*-value; Treg, regulatory T cell; UC, ulcerative colitis.

De novo clustering of data from the CosMx 6K and Xenium 5K panels similarly yielded spatial populations (Fig. 3a, Supplementary Fig. 8). As demonstrated in Fig. 1, the Xenium 5K panel had lower sensitivity for overlapping genes than the Xenium multi-tissue panel and substantially lower sensitivity than the CosMx 6K panel. *CSF3* is a gene known to be critical to UC pathology and inflammation^8,35^, and it is highly expressed in surface epithelial inflammatory niches in UC where transcript abundance is expected to be robust and therefore serves as a stringent test for platform sensitivity and potential impacts of optical crowding in inflamed UC. In an inflamed UC sample, the Xenium multi-tissue panel detected sixfold more cells expressing *CSF3* (colony-stimulating factor 3) than the larger Xenium 5K. However, both Xenium multi-tissue and Xenium 5K had substantially lower *CSF3* expression than CosMx 6K in the same spatial regions (Fig. 3b), despite the Xenium 5K panel having high confidence (Q-score) in probe detection (Supplementary Dataset 7). Similar to FOXP3, when removing the Q-score filter, we were able to identify a higher level of *CSF3* expression in Xenium data in the same spatial regions as CosMx. It is important to note that Q-scores assess probe-detection confidence once a transcript is detected; they do not account for sensitivity losses that manifest as dropout or under-detection, particularly in inflamed tissues. The reduced sensitivity and confidence of Xenium panels for a key marker of granulocytes in UC highlights the potential impact of platform performance on exploring these cellular populations in the NIDDK IBD consortiums, given granulocytes are known to be biologically relevant for dysplasia progression in UC and contribute to mechanisms of treatment response for hyperbaric oxygen therapy^16,26,36–39^.

DGE analyzes for the overlapping genes between both panels identified different biology for inflamed UC versus non-inflamed UC or healthy controls (Fig. 3c, Supplementary Fig. 7b, and Supplementary Dataset 6). *PGK1* (phosphoglycerate kinase 1) was identified by CosMx, but not Xenium, as a differentially expressed gene in UC. To determine whether the absence of *PGK1* as a DGE in Xenium reflected probe failure or tissue-specific sensitivity issues, we examined per-cell probe detection confidence across samples and cell types. All 4 samples separately demonstrated high average Q-scores (>30) for *PGK1* across cells in the Xenium 5K panels, however the range of Q-scores for *PGK1* varied substantially within each sample (Supplementary Dataset 7). A wider range in probe detection confidence was observed among cells for *PGK1* in the inflamed UC samples (Q-score range 9-40) compared to the non-inflamed UC sample (Q-score range 17–40). Further review of cell-type-specific variance in Q-scores for *PGK1* revealed that CD8+ T cells, Naïve/Memory T cells, and Tregs had a wider range in *PGK1* Q-scores in inflamed UC samples compared to non-inflamed UC or healthy rectum samples, and the lowest Q-scores for *PGK1* in T cells were seen in inflamed UC samples (Supplementary Dataset 8). CosMx 6K detected *PGK1* in twofold higher T-cells compared to Xenium 5K in inflamed UC (5% vs. 2%) even after including low Q-score gene detections for Xenium. T-cell specific DGE for Xenium 5K did not identify *PGK1* as a significantly differentially expressed gene due to the low overall detection and sensitivity for *PGK1* in inflamed UC.

Inhibition of PGK1 has been demonstrated to reduce colitis activity in mouse models^40^. The differential expression of *PGK1* in inflamed UC samples was greatest in Tregs (Fig. 3d, e). The expression profile of *PGK1* in T cells was confirmed using mouse splenic CD4^+^ T cells cultivated under Th0, Th1, Th2, Th17, and iTregs polarization conditions for 3 days (Fig. 3f). Recent work has demonstrated that inhibition of PGK1 attenuates myocarditis by reprogramming CD4^+^ T cell metabolism^41^, and we therefore sought to assess the impact of PGK1 inhibition in T cells for UC (Supplementary Methods – PGK1 experiments). In vitro treatment of Th17 cells with CBR-470-1, a PGK1 inhibitor, resulted in the significant upregulation of ROR-γt expression per cell (Mean Fluorescence Intensity (MFI)) (Fig. 3g–i). Treatment of Tregs (iTregs) with CBR-470-1 resulted in the significant upregulation of FOXP3 expression per cell (MFI) (Fig. 3j–l). There was no significant change in Treg or Th17 percentages with PGK1 inhibition. PGK1 upregulation may represent a compensatory response to inflammatory stress, limiting overactivation of both Th17 and Treg programs. The paradoxical effect on both lineages underscores the complexity of metabolic control in T cell fate and supports the idea of PGK1 as a rheostat rather than a binary switch. Future studies are needed to dissect whether PGK1 influences T cell lineage stability or plasticity under chronic inflammatory settings. Nonetheless, these experiments further support how platform-dependent sensitivity can alter biological inference in clinical cohort studies and highlight the value of high-resolution spatial atlases for resolving subtle inflammatory and metabolic programs in UC.

### Implementation of technology in multi-center collaboration studies

Our analyzes were done to guide the future implementation of imaging-based spatial technology in the CCF IBD Plexus program and NIDDK Consortiums. We therefore sought to assess technical aspects related to implementation that may influence data quality. Tissue thickness significantly impacted data quality, with 4–5 μm being the optimal thickness for IBD tissue sectioning with CosMx. (Supplementary Fig. 9) Optimal section thickness is particularly important in IBD given the dense immune cell infiltrates and architectural distortion, which can affect segmentation accuracy and transcript capture. Using biopsies obtained from a single donor (UC inflamed, 8 rectal biopsies) we applied differential fixation times (6 h, 24 h, 48 h, 72 h; *n* = 4 FFPE blocks with 2 biopsies per block) to mimic routine variability in pathology practices (i.e., weekend fixation for biopsies taken on Friday, or same day fixation for biopsies taken early in the day). We did not see any consistent impact of fixation time on data quality for CosMx (Supplementary Fig. 9). Minor fluctuations were observed between timepoints, but no systematic trend emerged, suggesting that CosMx is robust to fixation variability at the ranges commonly encountered in clinical practice. Using 4 FFPE blocks (*n* = 8 biopsies) we generated multiple tissue sections that were then stored for variable time periods before spatial profiling (0 weeks, 2 weeks, 4 weeks with or without vacuum storage to prevent oxidative damage, 6 weeks with or without vacuum storage to prevent oxidative damage). We did not see any consistent impact of delayed sectioning timing on data quality, and vacuum storage did not improve data quality (Supplementary Fig. 9).

**Fig. 4:**
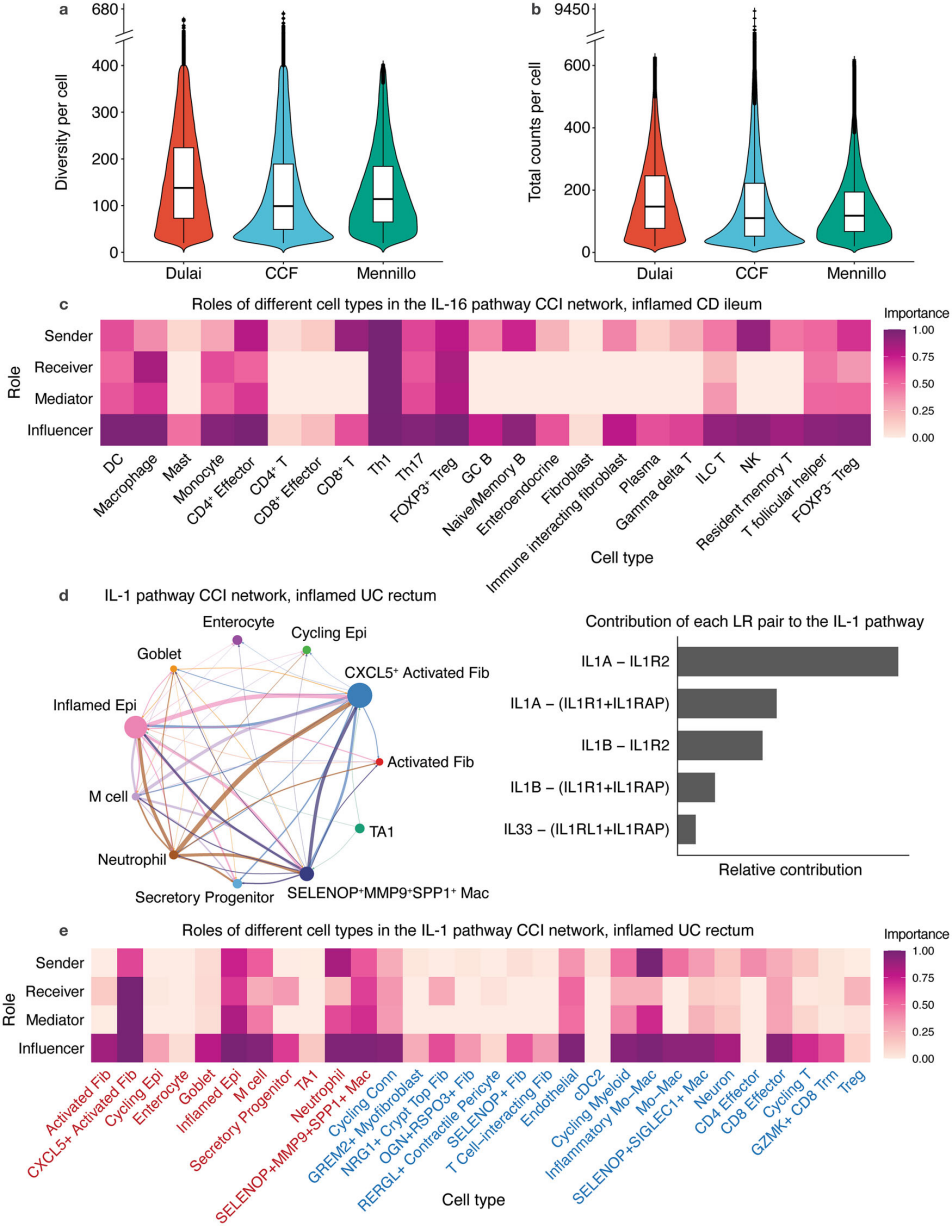
CosMx identifies cell-type-specific roles in IL-16 and IL-1 signaling pathways in inflamed Crohn’s disease and inflamed ulcerative colitis. The in-house, Crohn’s & Colitis Foundation (CCF), and external (Mennillo et al. 2024) CosMx multi-tissue datasets demonstrate similar data quality between CCF and Mennillo datasets, and higher **a** diversity and **b** total counts per cell for Dulai lab (Dulai lab: *n* = 324,449 cells, CCF: *n* = 255,035 cells, Mennillo et al.: 62,746 cells). The boxplots display medians and quartiles, with whiskers extending to 1.5 times the interquartile range, and the violin plot outlines represent the kernel probability density. **c** The roles of different cell types in the IL-16 signaling pathway cell-cell interaction network in inflamed Crohn’s disease ileum samples (129,747 cells). Standardized role importances are shown for cell types with non-zero role importance. **d** Cell–cell interactions among cell types of interest through the IL-1 signaling pathway in inflamed ulcerative colitis rectum samples (105,038 cells). Relative contributions of ligand-receptor pairs to the pathway are shown. **e** Cell-type roles in the IL-1 signaling pathway interaction network in inflamed ulcerative colitis rectum samples (105,038 cells). Standardized role importances are shown for cell types with non-zero role importance. Cell types of interest shown in **d** are colored red, and other cell types are colored blue. Source data are provided in the Source Data file. CCF, Crohn’s & Colitis Foundation; CCI, cell-cell interaction; CD, Crohn’s disease; cDC, conventional dendritic cell; DC, dendritic cell; Epi, epithelial; Fib, fibroblast; GC, germinal center; ILC, innate lymphoid cell; LR, ligand-receptor; Mac, macrophage; Mo, monocyte; NK, natural killer; Treg, regulatory T cell; Trm, tissue-resident memory T cell; UC, ulcerative colitis.

After determining that fixation time and sectioning timing did not impact data quality, we provided our standardized sample preparation protocol to the CCF IBD Plexus program with pre-labeled slides for cutting. In this cohort, Xenium performance showed greater dependence on RNA quality metrics, whereas CosMx performance was less sensitive to these factors within the tested range. Pre-screening of FFPE blocks for RNA quality for DV200% would therefore be needed, and review of the CCF IBD Plexus program FFPE blocks demonstrated that this would not be feasible without exhausting the entirety of tissue specimens. Therefore, we only ran CosMx on the 8 samples cut centrally. Overall data quality for the centrally cut CCF IBD Plexus program tissue was comparable to our data and other published IBD CosMx data^22^ (Fig. 4a, b). This cross-site concordance demonstrates that standardized preparation protocols can be reliably implemented across centers, a critical prerequisite for multi-institutional spatial trials.

Having established technical reproducibility, we next qualitatively evaluated whether biological signals detected in our benchmark dataset were consistently recapitulated in the centrally processed IBD Plexus program samples. Pathway analyzes for cell-cell interactions from the CCF IBD Plexus program inflamed ileal CD samples yielded some qualitative overlap to pathways of known biological importance in CD identified in our cell-cell interaction analyzes (i.e., GALECTIN, CCL, TNF). We identified *FOXP3*^+^ Tregs, macrophages, and monocytes as central contributors to the IL-16 signaling pathway cell-cell interaction network in the inflamed ileal CD samples from the CCF IBD Plexus program which may have been enabled by the larger number of cells present in this dataset (Fig. 4c). IL-16 has been demonstrated to preferentially induce migration of *FOXP3*^+^ Tregs^42^, and stimulation of monocytes and maturing macrophages with IL-16 has been demonstrated to result in the secretion of IL-1β, IL-6, IL-15 and TNF-α^43^. Pathway analyzes for cell-cell interactions from the CCF IBD Plexus program inflamed UC samples similarly yielded some qualitative overlap for multiple pathways identified in our data (i.e., OSM, VEGF, SPP1, CXCL, ICAM). Notably, there was a paucity of neutrophils annotated using the reference UC atlas from Smillie et al.^17^. Given the importance of studying neutrophils for the NIDDK consortiums, we re-annotated the CosMx multi-tissue inflamed UC samples from our in-house and CCF IBD Plexus program data with an alternative reference atlas that contained neutrophils^36^. Cell-cell interaction analyzes from our datasets identified IL-1 signaling between fibroblasts and neutrophils via IL1α-IL1 receptor type 1 (R1) and IL1-receptor accessory protein (RAP), consistent with prior reports^44^ (Fig. 4d, e). Our analyzes also identified IL-1 signaling between *SPP1* ^+^ Macrophages and neutrophils primarily via IL1β-IL1R2 (Fig. 4d), and this was identified independently in both our data and the CCF IBD Plexus program Biorepository samples. *SPP1* ^+^ Macrophages have been observed to interact with fibroblasts in colitis-associated cancer via IL1β signaling, and *SPP1* ^+^ Macrophages via IL1 promote the recruitment of CSF3R^+^ neutrophils^45,46^, a gene we observed to be better detected by CosMx than Xenium. The observation that both IL1α and IL1β may be important signaling mechanisms for neutrophil-mediated inflammation in UC helps provide a biological context for the investigation of Lutikizumab, an anti-IL1 α/β variable domain immunoglobulin, in moderate-severe UC. This re-analysis with an alternative single-cell RNA sequencing reference atlas highlights the importance of defining optimal strategies for annotation to guide accurate biological discovery.

### Cell type annotation and spatial multi-omics

**Fig. 5:**
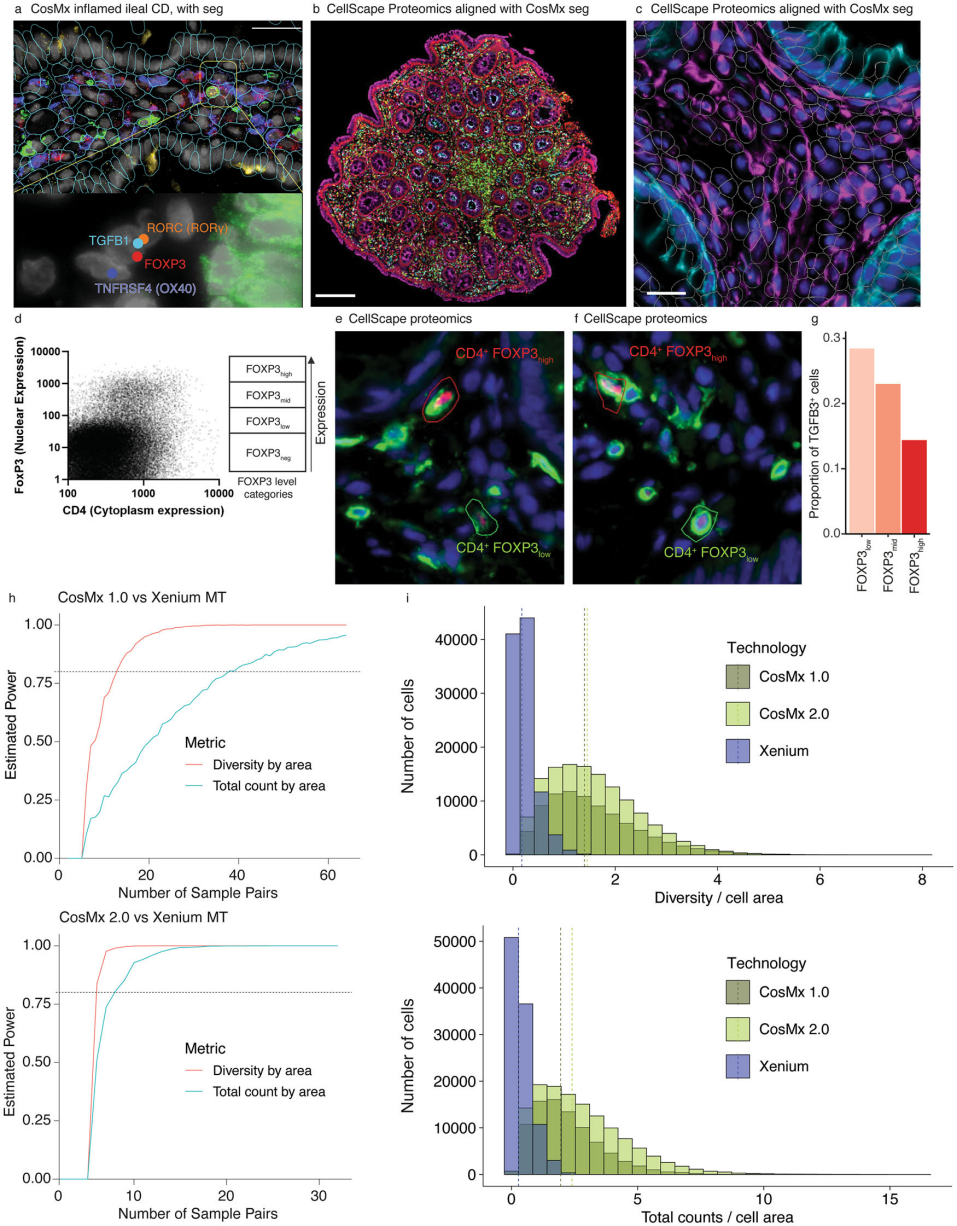
Future directions for spatial technology in cohort studies: spatial multi-omics, power calculations, and impacts of technology improvements. **a** Upper panel: immunofluorescence protein markers (CD3/CD4, green; FOXP3, yellow; CD163, blue; CD68, red) with CosMx multi-omics (aligned 64-plex spatial proteomic panel and 6K spatial transcriptomic panel) in an inflamed ileal CD sample. Lower panel: co-localization of a pro-inflammatory Treg expressing FOXP3/TGFB1/RORC with a macrophage expressing TNFRSF4 (OX40). Each dot represents a single CosMx transcript detection. Scale bar: 30 µm. **b** Representative tissue section showing pixel-level alignment between CosMx and CellScape data across magnification levels. Scale bar: 200 µm. **c** Overlay of CellScape protein markers (ATP1A1, cyan; CD45, magenta; DNA, blue) with CosMx-derived cell-segmentation boundaries (gray). Scale bar: 20 µm. **d** Scatterplot of single-cell CD4 (cytoplasmic) versus FOXP3 (nuclear) protein expression used to classify T cell subtypes (*n* = 37,009 cells; 4 FFPE blocks, 8 biopsies). **e**, **f** Immunofluorescence images showing representative CD4⁺ FOXP3^high^ and CD4⁺ FOXP3^low^ T cells. FOXP3 (red), CD4 (green), DNA (blue). **g** Bar plot showing proportions of *TGFB3* RNA-positive cells among CD4⁺ FOXP3^low^ (427 cells, 28.3% *TGFB3*^+^), CD4⁺ FOXP3^medium^ (310 cells, 22.9% *TGFB3*^+^), and CD4⁺ FOXP3^high^ (189 cells, 14.3% *TGFB3*^+^) T cell groups (4 FFPE blocks, 8 biopsies). *TGFB3* RNA is correlated with FOXP3 protein expression at the single-cell resolution. **h** Simulated power curves for paired platform comparisons showing statistical power versus number of paired samples. Power is estimated as the proportion of simulations yielding a two-sided Wilcoxon rank-sum test *p*-value < 0.05 for per-sample metrics (median total counts, blue; median diversity, red) comparing CosMx 1.0 versus Xenium MT (upper) and CosMx 2.0 versus Xenium MT (lower). The horizontal dashed line indicates the 80% power threshold. **i** Transcripts diversity (upper) and total counts (lower) by cell area across CosMx 2.0 (*n* = 132,727 cells; 6 FFPE blocks, 12 biopsies), CosMx 1.0 (*n* = 85,207 cells; 6 FFPE blocks, 12 biopsies) and Xenium MT (*n* = 101,500 cells; 6 FFPE blocks, 12 biopsies). Medians are shown as vertical dashed lines. Source data are provided in the Source Data file. CD, Crohn’s disease; FFPE, formalin-fixed paraffin-embedded; MT, multi-tissue; seg, segmentation.

The overall confidence in RNA-based annotation with Seurat label transfer of major cell types was higher for Xenium. (Supplementary Dataset 9) RNA-based annotation confidence for minor cell-types, however, was quite variable and lower for Tregs on both platforms across commercial panels. (Supplementary Dataset 9) The use of a protein-gating strategy did improve confidence in annotation for CosMx (Supplementary Dataset 9), suggesting that multi-omics-based annotation approaches may ultimately prove most ideal. The low overall confidence in the annotation of Tregs across platforms warranted further protein-based evaluation of biological observations. CosMx multi-omics (64-plex protein panel + 6K RNA panel) of new inflamed ileal CD samples demonstrated spatial co-localization of Tregs with macrophages using spatial proteomic marker-based annotations. We explored cell-cell interactions observed from our comparative analyzes in this multi-omics dataset and observed consistency using spatial transcriptomics expression (Fig. 5a). This orthogonal assessment in new samples provides further confidence in our biological insights derived from comparative benchmarking and supports the value of multi-omics.

### Quantitative spatial multi-omics and transcriptional heterogeneity

When performing spatial multi-omics in clinical research, customization of protein markers will be of tremendous importance, so panels can be fit-for-purpose based on biological insights and/or intended use. Ideally, spatial multi-omics could leverage complementary platforms run sequentially on the same slide for optimal data alignment. To test the feasibility of this, we performed CellScape, a multiplex quantitative immunofluorescent panel capable of using off-the-shelf primary conjugated antibodies, followed by CosMx on the same slide. We were able to successfully align the data and achieve CellScape and CosMx multi-omics with high-quality data from both platforms. (Fig. 5b, c) These samples allowed further exploration regarding the relationship between protein expression and transcriptional heterogeneity. As noted in our work and prior work in the field using dissociated scRNAseq, there appear to be both anti-inflammatory and pro-inflammatory *FOXP3*^+^ Tregs in IBD. Given the well-established role of FOXP3 as a master regulator of Treg function, we hypothesized that the relative quantitative protein expression of FOXP3 may identify Treg transcriptional subtypes. Indeed, Cellscape was able to spatially identify FOXP3^high^ Tregs, FOXP3^medium^ Tregs, and FOXP3^low^ Tregs. (Fig. 5d–f) These Treg sub-types were transcriptionally distinct with a higher proportion of FOXP3^low^ Tregs expressing TGFβ3 compared to FOXP3^medium^ Tregs and FOXP3^high^ Tregs. (Fig. 5g) TGFβ3 has known pro-inflammatory effects and IL-23 dependent TGFβ3 production in combination IL-6 induces highly pathogenic Th17 cells compared to TGFβ1+IL-6 induced Th17 cells in autoimmune disease^47^. This demonstrates the need for multi-omics and the potential ability to use quantitative proteomic multi-omics to build and/or train new annotation models that can be used to not only define cell types but also potentially define cellular transcriptional heterogeneity.

### Future directions: power calculations

An important consideration when implementing any technology into cohort studies or clinical trials is defining the potential impact of sample variance and evolutions in technology on power calculations. Given the rapidity of evolution in instruments and bioinformatics, we evaluated this potential impact by re-running samples on a recently upgraded CosMx platform (CosMx 2.0) to understand how instrument and bioinformatic upgrades may change data quality and how this might influence the interpretability of comparative studies or the future design of clinical studies. We observed our original comparative analysis of CosMx 1.0 versus Xenium to be sufficiently powered for the comparison of transcript diversity (unique genes), but not transcript counts (Fig. 5h). Comparative studies leveraging CosMx 1.0 would need a minimum of 10 samples, with biological replicates for each sample and entire tissue sections for each biopsy to capture tissue variability in data quality, for comparison of gene diversity (unique genes detection) and 30 samples, with biological replicates for each sample, for comparison of transcript counts (counts per gene detected) across platforms and all these samples should be from a single disease (e.g., IBD) and tissue type (e.g., gastrointestinal) to be sufficiently powered. Ideally, these samples should include various disease states for the tissue type to account for variability in performance of platforms in inflamed tissue, as we have demonstrated. Notably, CosMx 2.0 yielded improved data quality, which resulted in substantial improvements in power calculations. (Fig. 5h, i) This highlights that when performing studies or comparing across studies, investigators should account for instrument and software versions to avoid the introduction of batch effects from technical and/or bioinformatic upgrades. Further work is needed to understand how within-patient and pre-post intervention variance may further influence power calculations to accurately guide sample size estimates in clinical research. This is particularly relevant when considering the accuracy of cell-cell interactions after accounting for cellular proportion variance, cellular proximity variance due to sectioning variance, disease states, treatment exposures, and patient characteristics, and future work is needed to define these influences on precision for cell-cell interactions in power calculations. Ideally, future power calculations will focus on minimum replicates and samples needed to identify biology and clinically meaningful variables.

## Discussion

We have provided a systematic evaluation of two leading spatial imaging platforms for single-cell spatial transcriptomics on FFPE tissue to guide use of this evolving and expensive technology for NIDDK-supported IBD research consortiums and the CCF IBD Plexus program Biorepository. Together, these analyzes reveal how platform-specific technical constraints—particularly sensitivity in inflamed tissue and dependence on RNA quality—directly shape biological discovery in cohort-level spatial studies. We observed that within the intestinal samples evaluated in this study: (1) CosMx demonstrated higher gene detection and quantification compared to Xenium in IBD FFPE tissue blocks, (2) Xenium exhibited a significant reduction in performance with increasing plex size, (3) Xenium, but not CosMx, was significantly influenced by tissue type (inflamed tissue) and block quality (DV200%) thereby requiring pre-screening of tissue blocks to ensure feasibility which was observed to be impractical for the CCF IBD Plexus program Biorepository, (4) higher sensitivity observed with CosMx in inflamed FFPE tissue enabled detection of biological signals that were not robustly captured by Xenium, and (5) CosMx demonstrated more consistent performance in this cohort across samples with variable fixation time, variable slide preparation timing, and slides cut remotely by the CCF IBD Plexus program Biorepository supporting its use for multi-center research consortiums and clinical trials for IBD and related diseases. Finally, we provide a large single-cell spatial atlas for the research community and anticipate that our work may help shape the utilization of spatial transcriptomics for other GI clinical trials and consortiums^48,49^.

Previous comparative studies have suggested that Xenium may be technically superior to CosMx^19–25^. These studies have limited sample sizes and lack sufficient power, along with technical and methodological differences that make it difficult to extrapolate observations to all tissue types and disease states. Notably, they lack a direct comparison of platforms within a clinical cohort study design to understand how performance variability influences biological discovery. After accounting for data quality, filtering, and variations in cell segmentation approaches, we observed that variability in probe detection confidence and sensitivity for Xenium in inflamed IBD tissue was a major contributor to discordant results between platforms when performing cohort-level analyses. These discrepancies were not random; they disproportionately affected biologically informative genes with low-to-moderate expression levels, particularly within T cells and granulocytes in inflamed mucosa. Our observations related to *FOXP3* and *PGK1* highlight that greater attention is needed for gene-level probe detection confidence for Xenium for each sample and cell type, and researchers will need to account for this potential variance across disease states when conducting cohort-level studies with this platform. Although analogous probe-level confidence metrics are not available for CosMx, orthogonal validation through multi-omics and functional assays, and the well-accepted biological understandings of the disease, provided additional support for the biological signals observed in this context.

Prior comparative analyzes assumed that Xenium would have no meaningful change in performance across blocks from different disease states and/or with increasing plex size. Comparisons were therefore made primarily in non-diseased or non-inflamed samples, and they were normalized to the smaller plex size of the Xenium multi-tissue panel^28^. Our data suggests a substantial drop off in performance for Xenium with lower quality blocks as measured by DV200% and negative probes, greater variability in probe detection efficiency for Xenium with inflamed IBD tissue, and a reduction in sensitivity with increasing panel size. This suggests that the increased optical and molecular complexity of higher-plex designs may exacerbate sensitivity limitations in structurally complex or inflamed tissues. The exact reasons for these observations could not be fully discerned from the data; however, possible explanations could include RNA fragmentation, background non-specific binding, optical crowding, limitations in amplification technology for Xenium, and/or the change in chemistry for the 5K panel. Nonetheless, this demonstrates that clinical research programs need to take these factors into consideration through pre-screening of tissue blocks and more in-depth data quality assessments on a per-sample and per-gene basis to ensure sufficient quality to yield meaningful data. CosMx, however, showed comparatively consistent performance in this study across samples with varying quality, disease states, fixation, preparation, and panel size. The extensive time needed for each run, often 2–3 days of prep and up to 2 weeks of instrument time, and the extensive cost in the several thousands of dollars per slide associated with these experiments, makes it important to maximize value in clinical research. Among the platforms evaluated, CosMx exhibited greater stability (e.g., in data quality and detection efficiency) in a multi-center IBD cohort study, and this may make it well-suited for large multi-center collaborations and clinical trial programs for IBD focused on biological discovery.

Beyond benchmarking, our work provides a blueprint for scaling spatial transcriptomics within multi-center research programs. A persistent challenge for the field is the lack of harmonized spatial reference datasets that support reproducible power calculations, sample-size estimation, and multimodal integration. As spatial technologies diffuse into clinical research, such reference datasets will become essential for reproducible power calculations, annotation consistency, and biologically informed model development. To address this need, future datasets from the NIDDK IBD consortia and CCF IBD Plexus program can be incorporated into a multi-organ, open-access spatial reference framework such as the Spatial Atlas of Human Anatomy (SAHA)^50^. Integrating IBD datasets within SAHA will enable cross-cohort harmonization and support the development of advanced models for spatial remodeling, cell–cell communication, and treatment response.

Although our study was conducted with rigor, it does have certain limitations. We did not evaluate the other available imaging-based single-cell spatial transcriptomics platform (MERSCOPE) because the plex size is limited compared to CosMx and Xenium. The intent of our study was to define the ideal platform for spatial biology discovery, and we therefore cannot comment as to which platform is ideal for small plex or custom experimental designs. In this regard, Xenium did identify differentially expressed genes which were not captured by CosMx, and Xenium may offer some advantages through custom panel creation and small panel designs for discrete experimental questions, given the observed performance for its colon-specific panel in comparison to larger panels. It will be important to ensure the custom panel genes have high confidence in detection across tissue types, and it is important to note that all 3 platforms have distinct approaches to custom probe design, with recent data suggesting Xenium panels may have off-target probe binding^51^. This off-target probe binding has been demonstrated with other 10× platforms (Visium) as well^52^. Similar evaluations for CosMx were not feasible because the probe sequences are not publicly available; however, our in-vitro validation and multi-omic orthogonal confirmation of biological observations made by CosMx provide some confidence that the more consistent performance in comparisons was not driven by false positive detections. Custom probe development and comparisons across platforms will need to consider this potential risk, and further work is needed for all spatial platforms in this regard. Along these lines, further work is needed to define optimal approaches to data quality control and filtering with consideration for probe specificity, probe gene detection confidence, and impacts on cellular annotations and cellular interactions, ideally leveraging protein-based annotation as ground truth through multi-omics. A detailed and biologically driven methodology for data filtering is yet to be developed for uniform application in spatial imaging-based technologies.

In conclusion, we provide a comprehensive evaluation of two high-plex imaging-based single-cell spatial transcriptomics platforms in IBD. By linking technical performance to downstream biological inference, these findings provide a framework for selecting and deploying spatial platforms in translational and clinical research. These results will guide the use of these platforms for two large NIDDK-funded IBD consortiums as well as the CCF IBD Plexus program Biorepository, and we anticipate these results may help guide the use of single-cell spatial transcriptomics for other GI research collaborations and/or initiatives. The accompanying spatial atlas serves as a foundational resource for benchmarking, annotation, and hypothesis generation in IBD research and establishes a framework that can be extended to emerging multi-organ spatial datasets.

## Methods

Our study complies with all relevant ethical regulations. FFPE samples were obtained through the Northwestern Digestive Health Foundation (DHF) IBD Biorepository, approved by the Northwestern University Institutional Review Board (IRB: STU00203172), as well as through the centrally stored CCF IBD Plexus program Biorepository (Project #1300564), approved by a central Institutional Review Board at the University of Pennsylvania (IRB protocol: 823980). All donors provided written informed consent for the use of their samples and associated data in the present study, including sharing of data, prior to sample collection and analysis. Appropriate protocols governing sample use were reviewed and approved by the respective Institutional Review Boards listed above prior to the conduct of experiments. All human tissue samples analyzed in this study were collected in a cross-sectional manner, and no post-intervention samples were included. Animal use and care were conducted in accordance with institutional guidelines at Northwestern University, and all experiments were approved by the Institutional Animal Care and Use Committee of Northwestern University (IACUC protocol: IS00026385).

### Sample procurement and preparation

We aimed to systematically and rigorously compare CosMx and Xenium in intestinal samples across commercially available panels to guide decisions on platform utilization for the Crohn’s & Colitis Foundation (CCF) IBD Plexus program Biorepository as well as two ongoing NIDDK funded IBD research consortiums: (1) a clinical trial of hyperbaric oxygen therapy in UC where spatial transcriptomics will be used to understand mechanisms of action^53^, and (2) a clinical trial of dysplasia surveillance in IBD where spatial transcriptomics will be used to understand mechanisms of dysplasia development and/or progression.

The Northwestern DHF IBD Biorepository is a fully de-identified prospective cohort of IBD patients recruited at Northwestern Medicine, and samples were collected and processed prospectively in a systematic and uniform approach to maintain consistent sample quality and comparability. FFPE blocks were obtained through the Northwestern Digestive Health Foundation (DHF) IBD Biorepository (IRB: STU00203172; Supplementary Dataset 10). IBD patients are consented in clinic and/or endoscopy for the collection, use, and/or sharing of data or samples from the procedure. A standardized biopsy collection protocol is used with routine collection from the ileum, right colon (cecum/ascending), left colon (descending/sigmoid colon), and rectum. Biopsies are targeted for the most inflamed portion of each segment, and if no inflammation is present, then they are taken from areas adjacent to where routine care biopsies are taken to most closely align with routine care histology assessments. Disease activity is measured using standardized endoscopic indices for Crohn’s disease (Simple Endoscopic Score for Crohn’s Disease) and ulcerative colitis (Mayo endoscopic sub-score). Biopsies are obtained and immediately placed in formalin jars and transported immediately to the biorepository where a dedicated lab technician allows for a fixation of 24 h followed by transfer to 70% ethanol and stored at 4° in a refrigerator until they are transferred to the Northwestern University Pathology Core for automated embedding, cataloging, and H&E imaging. A wide range of tissue types were selected for the comparative study (inflamed or non-inflamed; ileum or rectum; CD, UC, or healthy; *n* = 2 biopsies per location for each FFPE block to account for intra-patient variability; *n* = 40 total mucosal biopsies in 20 FFPE blocks). A second set of samples (inflamed or non-inflamed UC, or healthy; *n* = 2 biopsies per location for each FFPE block, *n* = 16 total mucosal biopsies in 8 FFPE blocks) was selected for evaluating technical aspects related to implementation in consortiums or multi-center studies that may influence data quality.

The CCF IBD Plexus program Biorepository is a multi-center collaboration, and the IBD Plexus program samples were obtained from the Study of a Prospective Adult Research Cohort with IBD (SPARC IBD), a component of the Crohn’s & Colitis Foundation IBD Plexus program research platform^24^. The samples were collected and processed prospectively using a standardized protocol where biopsies were taken from the most inflamed section or from standardized locations when no inflammation was present^48,49^. Given the multi-center nature of the study, FFPE blocks from the CCF IBD Plexus program Biorepository had natural variation in biopsy size, processing time, shipping, and/or fixation times. These samples therefore, offered a representation of expected performance for platforms within multi-center consortiums or clinical trials. A range of tissue types and disease states were selected for the study (inflamed or non-inflamed ileum CD, inflamed rectum UC; *n* = 8 FFPE blocks, 1–2 biopsies per FFPE block). Slides were cut centrally by the CCF IBD Plexus program Biorepository technician and shipped to Northwestern University for spatial profiling to mimic anticipated applications.

### CosMx and Xenium slide preparation

To ensure consistency and avoid biases in evaluations, the following occurred with all comparative runs: (1) sequential cuts of the FFPE blocks were done by a single senior experienced pathology technician (Northwestern University Pathology Core) and placed on dedicated slides for CosMx and Xenium at the same time (*Tissue sectioning and preparation*), (2) Xenium samples were run by an independent 10x Genomics certified service provider (Northwestern University Sequencing Core), (3) preparation for each platform was done simultaneously in respective locations (Xenium – Northwestern Sequencing Core; CosMx – Dulai lab; *CosMx sample preparation and Xenium sample preparation*) by experienced senior technicians within 1 week of sectioning, (4) field of view placement was gridded for the entire tissue specimen(s), (5) comparisons were done for entire panels and separately for overlapping genes present in platforms’ panels being assessed, and (6) the technical support teams from each company were provided an opportunity to review their instruments data quality to ensure we did not include technical run failures in comparisons. Neither company requested that any samples be re-run, and all runs passed recommended quality control parameters by both companies independently.

#### Tissue sectioning and preparation

Sectioning was done by the Northwestern University Pathology Core, and full block sections were used for all experiments. The comparative analysis of CosMx and Xenium used a standard section thickness of 5 μm, consistent with 10× Genomics recommendations. Sequentially cut sections were placed on the CosMx and Xenium slide capture areas to ensure no variation in microtome settings, cutting, or block section variation might influence comparative analyzes. Sample preparation followed recommended protocols and procedures by manufacturers^8,19–21^. The CosMx slide processing and CosMx instrument loading were done in the Dulai lab. The Xenium slide processing and Xenium analyzer loading were done at the Northwestern University Sequencing facility core.

#### CosMx sample preparation

Tissue sections were mounted on Leica Biosystems Apex BOND Superior Adhesive Slides (cat# 3800040) and subsequently baked overnight at 60 °C, followed by preparation for in-situ hybridization (ISH) by deparaffinization in Citrisolv (2× 5 min), 100% ethanol (2× 5 min) and heat-induced epitope retrieval (HIER) at 100 °C for 15 min using low pH citrate buffer (NanoString/Bruker). After HIER, tissue sections were digested with Proteinase K diluted in PBS at 40 °C for 30 min. Tissue sections were then washed twice with diethyl pyrocarbonate (DEPC)-treated water (DEPC H_2_O) and incubated in 0.0015% fiducials (NanoString/Bruker) in 2X saline sodium citrate, 0.001% Tween-20 (SSCT solution) for 5 min at room temperature in the dark. Excess fiducials were rinsed from the slides with 1X PBS, then tissue sections were fixed with 10% neutral buffered formalin (NBF) for 5 min at room temperature. Fixed samples were rinsed twice with Tris-glycine buffer (0.1 M glycine, 0.1 M Tris-base in DEPC H_2_O) and once with 1X PBS for 5 min each before blocking with 100 mM N-succinimidyl (acetylthio) acetate (NHS-acetate, Thermo Fisher Scientific) in NHS-acetate buffer (0.1 M NaP, 0.1% Tween pH 8 in DEPC H2O) for 15 min at room temperature. The sections were then rinsed with 2X saline sodium citrate (SSC) for 5 min, and an Adhesive Hybridization Chamber (NanoString/Bruker) was placed over the tissue. ISH probes were prepared by incubation at 95 °C for 2 min and submerged in ice, and the ISH probe mix was pipetted into the hybridization chamber. The chamber was sealed to prevent evaporation, and hybridization was performed at 37 °C for 18 h. Tissue sections were washed twice in 50% formamide (Ambion) in 2X SSC at 37 °C for 25 min, washed twice with 2X SSC for 2 min at room temperature, and blocked with 100 mM NHS-acetate in the dark for 15 min. In preparation for loading onto the CosMx SMI instrument, a flow cell (NanoString/Bruker) was affixed to the slide.

#### CosMx multi-omics sample preparation

Proprietary reagents were provided by Bruker Spatial Biology, and protocols were per the Manual Slide Preparation Protocol. In summary, in situ protein expression followed the procedures, taking 5 mm paraffin-embedded tissue sections mounted on Apex BOND (Leica Biosystems, Deer Park, IL, USA) through quantification of expression on the CosMx spatial molecular imager platform (Nanostring, Seattle, WA, USA). Initially, formalin-fixed, paraffin-embedded tissue sections were mounted on glass slides and dried overnight at 60 °C, then deparaffined with Citrisolv (Decon Labs., Inc., King of Prussia, PA, USA), washed in 100% then 70% ethanol and finally 1× PBS. Sections were subject to antigen retrieval in target retrieval buffer (citrate-based) at 100 °C for 15 min and allowed to cool in buffer for 25 min. Following PBS washes, tissue was blocked with buffer W for 1 h. Primary antibodies (64 total) and antibody markers CD298/B2M, PanCK/CD45, and CD3 were incubated on tissue for 16–18 h in buffer W. Following washes in 1× TRIS-buffered saline with Tween-20 fiducials (0.00005%) were applied. Following 1× PBS washes, tissue was incubated for 15 min with DSP (Pierce, Rockford, IL, USA) solution, washed and incubated with DAPI stain, washed and treated with NHS acetate (Sulfo-NHS-Acetate, ProteoChem, Hurricane, UT, USA), prior to washing in 1× PBS and flowcell assembly. Flow cells were loaded onto a CosMx machine and the protein run was initiated. FOV regions were selected, and expression data generated and stored in AtoMx version 2.1 for analysis. The protein expression study was followed by RNA analysis on the same tissue slides; the flowcell cover was carefully removed with a disposable scalpel blade under 1× PBS. Tissue was then treated for 15 min with 50 mM TCEP (Millipore Sigma, St. Louis, MO, USA) and washed in 1× PBS. Tissues were incubated with Subtilisin at 40 °C for 30 min. After washing, fiducials were applied at 0.01% for 5 min, washed, and incubated for 16–18 h with 1000 plex RNA in situ hybridization probes in 0.5 nM rRNA. Tissues were washed in 50% formamide 2× saline-sodium citrate buffers, and DAPI stain was applied. Following 1× PBS washes, the tissues were incubated for 1 h with antibody markers CD298/B2M, PanCK/CD45, and CD68. Tissues were washed in 1× PBS and treated with NHS acetate, washed again in 1× PBS, and flow cells assembled. Flow cells were loaded onto the same CosMx machine and the same positional placement within the machine as used for the protein run. FOV regions very closely matching the protein run were selected, and RNA expression data generated and stored in AtoMx v2.1 for analysis. Cells were annotated using protein expression and a cell type marker approach, with subsequent visual confirmation of protein staining quality and examination of staining distribution patterns in AtoMx. For the RNA data paired at the single-cell level to each sample, expression of target genes was visualized using Seurat’s ImageFeaturePlot function, with the default boundary set to “segmentation”.

#### Cellscape sample preparation

Multiplex immunofluorescence staining and imaging were performed using the CellScape™ Precise Spatial Proteomics platform (Bruker Spatial Biology, Saint Louis, MO, USA). Human FFPE intestinal biopsy samples were deparaffinized and rehydrated according to the CellScape™ User Manual (Bruker Spatial Biology, Saint Louis). Briefly, samples were submerged for 5 min three times in Histo-Clear II (catalog no. 6411101; Electron Microscopy Sciences, Hatfield, PA, USA), two times in 100% Ethanol (EtOH), one time each in 90% EtOH, 70% EtOH, 50% EtOH, 30% EtOH, and finally CellScape Wash Buffer (catalog no. PRSM-BUF-WASH-500mL; Bruker Spatial Biology, Saint Louis, MO, USA). Immediately following deparaffinization heat-induced epitope retrieval (HIER) was performed at 110–120 °C under low pressure in a pressure cooker with samples placed in plastic Coplin jars (Fisher, Waltham, MA, USA) filled with Discovery CC1 buffer (catalog no. 06414575001; Roche, Basel, Switzerland) for 15-min. Samples were allowed to cool for 25 min on the benchtop. Slides were then washed with CellScape Wash Buffer and mounted with the CellScape Whole-Slide Imaging Chamber (catalog no. PRSM-CSWSIC-010; Bruker Spatial Biology, Saint Louis, MO, USA), and the microfluidic chamber was immediately filled with CellScape Storage Buffer (catalog no. PRSM-BUF-STR-50mL; Bruker Spatial Biology, Saint Louis, MO, USA) and stored at 4 °C until use.

The 34-plex assay was performed on the CellScape platform using automated iterative cycles of staining, imaging, and photobleaching. The assay consisted of 12 cycles, with each cycle beginning with a 10 s enhanced photobleach in the presence of EpicIF (catalog no. PRSM-BUF-EPIC-500mL; Bruker Spatial Biology, Saint Louis, MO, USA) and subsequent background measurements for each channel to be stained. Automated staining was performed with up to 3 antibodies incubated for 60 min per cycle. Following incubation, unbound antibodies were washed off with CellScape wash, and fluorescence images were acquired. The 34-plex assay included VistaPlex Cell Boundaries (catalog no. VISTAPLEX3101; Bruker Spatial Biology, Saint Louis, MO, USA), Immune Profiling (catalog no. VISTAPLEX3102; Bruker Spatial Biology, Saint Louis, MO, USA), and Architecture (catalog no. VISTAPLEX3103; Bruker Spatial Biology, Saint Louis, MO, USA) kits.

#### Xenium sample preparation

Sections placed on Xenium slide capture area, followed by 42C incubation of 3 h and overnight drying. Then, slides were stored at room temperature in the desiccation chamber until proceeding to deparaffinization. Slide processing and Xenium analyzer loading were carried out using Xenium slide and sample prep reagent kit (PN1000460), Xenium decoding consumables (PN-1000487), and Xenium decoding reagent (PN-1000461). Slides were first deparaffinized and de-crosslinked according to manufacturers’ protocol (CG000580). Then, probes with pre-designed panel genes were hybridized to sample slide overnight, following by probe ligation and amplification, according to manufacturers’ protocol (CG000582 for Xenium In situ gene expression; CG000749 for Xenium in situ gene expression with cell segmentation; and CG000760 for Xenium Prime in situ gene expression with cell segmentation). Autofluorescence quenching and nuclei staining were also performed before imaging and decoding signals on Xenium Analyzer.

#### Comparison of CosMx and Xenium data

To compare the quality of CosMx multi-tissue, Xenium multi-tissue, Xenium colon-specific, CosMx 6K, and Xenium 5K data, we utilized various cell-level quality metrics, including transcript diversity (the total number of unique genes detected within a cell), transcript total count, and total negative probes (negprobes). Cell-level metrics were evaluated and compared on a per-cell level and then also after being divided by cell area (μm^2^) to account for differences arising due to variations in cell segmentation between platforms. A subset of FFPE tissue blocks (*n* = 16 FFPE blocks, *n* = 32 biopsies) underwent assessment after run completion for RNA quality (DV200%) and RNA quantity (QuBit, ng/uL). Blocks were not pre-selected based on DV200% or QuBit to help ensure our comparison is most closely aligned with how end users might use the technology within large consortiums (e.g., NIDDK IBD consortiums), biorepositories (e.g., Crohn’s & Colitis Foundation IBD Plexus program), and/or clinical trial programs, where pre-screening and tissue exhaustion are not feasible or practical. We further performed Kendall rank correlation analyzes among DV200%, QuBit (ng/μL), median diversity by cell area (full and overlapping panel), median total count by cell area (full and overlapping panel), mean total negprobes by cell area, and proportion of cells with detected negprobes on CosMx multi-tissue and Xenium multi-tissue data, respectively. Wilcoxon rank-sum tests were conducted to analyze the difference between the same quality metric (transcript diversity by cell area and total count by cell area) within matched CosMx and Xenium samples. Additionally, linear mixed models were fitted with a quality metric as the outcome variable, platform (CosMx or Xenium) as a fixed effect, and sample key as a random effect, to further account for the variability in data quality across samples. All the *p*-values were adjusted for multiple testing using the FDR approach, and we assumed statistical significance at an adjusted *p*-value of $q < 0.05$. All data processing and analysis were conducted using R V4 and Seurat V5^54^.

#### The effect of sample preparation conditions on CosMx data quality

To study how sample preparation conditions affected the quality of CosMx multi-tissue data, we regressed different cell-level metrics (transcript diversity by cell area, transcript total count by cell area, total falsecode by cell area, and total negprobes by cell area) on tissue thicknesses (μm; 4–7 μm tested) and fixation time (hours; 6–72 h tested) in samples without any delayed cut, and estimated the 95% confidence interval of the regression coefficients. We further visualized the distribution of different cell-level metrics (transcript diversity by cell area, transcript total count by cell area, and total falsecode by cell area) across different delayed cut time length (no delayed cut, 2, 4, or 6 weeks), and vacuum storage among delayed cut samples (no or yes) using violin plots. We also visualized whether the cell’s being free of negprobes was independent of its sample preparation conditions using mosaic plots^55^. When assessing the impacts of sample preparation, we did not introduce additional protocol modifications to avoid confounding effects between the two levels of modifications. All data processing and analysis were conducted using R V4 and Seurat V5^54^.

### CosMx and Xenium data analysis

#### Quality control and data processing

We performed quality control (QC) on CosMx multi-tissue, Xenium multi-tissue, Xenium colon-specific, CosMx 6K, and Xenium 5K data. There existed no widely adopted best practices for choosing the QC thresholds for CosMx and Xenium, so we chose thresholds based on data visualization and panel size for each platform. For CosMx multi-tissue, we performed cell QC by removing the cells with fewer than 20 unique molecular identifiers (UMIs). For Xenium multi-tissue and Xenium colon-specific, we performed cell QC by removing the cells with fewer than 5 UMIs. For CosMx 6K and Xenium 5K data, given the panel size similarity, we tested how different QC thresholds affected the post-QC number of cells, transcript total counts, and transcript diversity (Supplementary Fig. 5). We observed an inflection point at UMI = 50 for several Xenium 5K samples and no obvious inflection point for CosMx 6K data. To ensure that subsequent comparisons using QC-ed data are fair, we performed cell QC on CosMx 6K and Xenium 5K data by excluding the cells with fewer than 50 UMIs. Sensitivity analyzes are presented with no QC threshold applied.

After QC, we normalized and transformed the raw datasets using log normalization (each cell’s gene counts are divided by its total counts, multiplied by 10,000, and log transformed). For CosMx 6K and Xenium 5K data, the top 3000 highly variable genes were next identified. After further scaling the data, we performed principal component analysis on the normalized data and clustered the cells through a shared nearest neighbor approach. All data processing was conducted using R V4 and Seurat V5^54^.

#### Extracting nuclear and non-nuclear counts

For CosMx data, we extracted the nuclear and non-nuclear counts from the transcript coordinates file based on the CellComp column which indicates the subcellular location of target^56^. For Xenium data, we used Xenium Ranger 2.0 to generate a nucleus-only count matrix by setting the expansion distance to 0 µm, as documented by 10× Genomics^57^. The non-nuclear count matrix was next obtained by subtracting the nucleus-only count matrix from the original count matrix on a per-feature basis.

#### Power analysis for platform data quality comparison

We compared platform data quality at the sample level using paired summaries. For each sample and each platform, we computed the median total counts by cell area and median diversity by cell area to summarize cell-level metrics. Paired differences between CosMx and Xenium at the sample level were next tested using two-sided Wilcoxon rank-sum tests.

To estimate statistical power, we adopted a simulation-based approach. We modeled the empirical distribution of paired differences for each metric as a Gaussian with mean and standard deviation equal to the observed mean paired difference between platforms and the standard deviation of differences across sample pairs, respectively. Using the estimated parameters, we simulated 5000 datasets by repeatedly sampling paired differences from the normal distribution. For each simulated dataset, we performed a two-sided Wilcoxon rank-sum test, and computed power as the proportion of datasets returning significant results (*p*-value < 0.05). We next iterated this procedure across a range of sample sizes to generate power curves and determined the minimum number of sample pairs required to achieve 80% power for each metric. Data analysis was conducted using R V4.

#### Cell type annotation

We performed cell type annotation on the processed data using Seurat’s label transfer approach^58^. The healthy, ulcerative colitis (UC) and Crohn’s disease (CD) samples were annotated separately using scRNA-seq data of the matching conditions^7,17^. Since CosMx data provided protein markers in addition to RNA data, we performed annotation using a protein-gated approach after directly visualizing staining to confirm biologically appropriate localization based on tissue architecture. Pancytokeratin (PanCK) is a known marker for epithelial cells^59^, and CD45 is a known marker for immune cells^60^. We visually examined the spatial distribution of Max.PanCK and found that 5000 was a reasonable cut-off for defining epithelial cells across all the samples. On the other hand, immune cells do not typically have a defined spatial organization. Therefore, we performed minimum-maximum scaling on the Max.PanCK and Max.CD45 markers and used the scaled value that corresponded to Max.PanCK = 5000 in the sample as the cut-off for scaled Max.CD45. In summary, our gating approach consisted of three steps. Firstly, we identified the epithelial cells with Max.PanCK = 5000. Secondly, the remaining cells were divided into immune cells and others based on the scaled Max.CD45 values. Finally, the three cell groups (epithelial, immune, and others) were annotated separately using Seurat’s label transfer approach^58^ and a subset of the scRNA-seq reference containing only cells of the matching category. We found that compared with the pure RNA approach, the protein-gated approach provided more consistent cell type annotation results (Supplementary Fig. 1). For each annotated cell type, we performed Wilcoxon rank-sum tests to examine if the cells of this cell type had significantly different cell areas in CosMx multi-tissue and Xenium multi-tissue (no segmentation) data. All the *p*-values were adjusted for multiple testing using the FDR approach, and we assumed statistical significance at an adjusted *p*-value of $q < 0.05$.

Given the importance of studying neutrophils for the NIDDK consortiums, we also provided alternative annotations of CosMx multi-tissue inflamed UC samples from the in-house and CCF IBD Plexus program datasets with an alternative reference atlas that contained neutrophils^36^. We visually re-examined the spatial distribution of Max.PanCK and found that 5000 stayed a reasonable cut-off for defining epithelial cells across all the samples. Therefore, in the annotation, we first identified the epithelial cells with Max.PanCK = 5000 and next annotated the two cell groups (epithelial and others) separately using Seurat’s label transfer approach^58^ and a subset of the scRNA-seq reference containing only cells of the matching category. All data processing and analysis were conducted using R V4 and Seurat V5^54^.

#### Differential gene expression analysis

Our CosMx multi-tissue and Xenium multi-tissue data were based on 16 samples from seven different sample categories—ileal inflamed CD, ileal non-inflamed CD, rectal inflamed CD, rectal non-inflamed CD, ileal non-inflamed UC, rectal inflamed UC, and rectal non-inflamed UC. To identify the genes differentially expressed in different sample categories, we performed Wilcoxon rank-sum tests comparing each sample category versus the others on the merged CosMx multi-tissue and Xenium multi-tissue datasets, respectively, using SCTransform, a framework for the normalization and variance stabilization of molecular count data^61^, and the FindAllMarkers function in Seurat V5^54^. The testing was limited to the genes detected in a minimum of 1% cells in either compared group and showing at least 0.1 log fold difference between the compared groups. The CosMx 6K and Xenium 5K data were generated from four rectum samples representing three different conditions—healthy, inflamed UC, and non-inflamed UC. To identify differentially expressed genes across these conditions, we performed Wilcoxon rank-sum tests comparing each pair of sample conditions on the merged CosMx 6K and Xenium 5K datasets, respectively, using SCTransform^61^ and the FindAllMarkers function in Seurat V5^54^. SCTransform was performed for all genes in each platform’s panels separately, as this is how the panels would be intended for use in cohort studies and clinical trials, where the entirety of the panel is used. We restricted the analysis to genes detected in at least 10% of cells in either compared condition and with at least 0.1 log fold difference between groups, and we present results for overlapping genes between panels to guide interpretation of comparative detection of differential genes between platforms. All the *p*-values were adjusted for multiple testing using the FDR approach, and we assumed statistical significance at an adjusted *p*-value of $q < 0.05$.

#### Cell-cell interaction analysis

To investigate the interactions among different cell types in ileal inflamed CD, we performed pathway-based spatial cell-cell interaction analyzes on the CosMx multi-tissue data of ileal inflamed CD samples (in-house: I0294 ITDF00A and I0275 ITDF00A; CCF: 7771, 772A, and 720A) and rectal inflamed UC samples (in-house: I0321 RSDF00A, I0286 RSDF00A, I0284 RSDF00A, and I0277 RSDF00A; CCF: 877A and 3774) using CellChat 2.1.2^62,63^. CellChat infers spatially proximal cell-cell interactions between cell groups on the signaling pathway level based on spatial transcriptomics data by summarizing the communication probabilities of all ligand-receptor interactions associated with each signaling pathway^62,63^, enabling insights into localized cell interactions specific to the inflamed CD environment. To characterize the roles of different cell types in different cell-cell interaction networks, we also calculated network centrality metrics, including weighted out degrees (sender role), weighted in degrees (receiver role), flow betweenness score (mediator role), and information centrality score (influencer role), using CellChat^62,63^. In our cell-cell interaction analyzes, pathway-level results were used as the primary unit of interpretation, while individual ligand or receptor genes were examined in the context of their contribution to broader signaling pathways rather than as standalone findings. This framework reduces arbitrariness in interpretation and ensures that the analytical results are supported by existing literature.

#### Co-localization analysis

To examine the co-localization between *PGK1*^+^ Tregs and other cell types, we conducted cell type co-occurrence analyzes on CosMx 6K data from an inflamed UC sample (I0387 RSDF00A). The co-occurrence scores between *PGK1*^+^ Tregs and all the cell types were computed using Python 3.12, SCANPY 1.10.3^64^, and Squidpy 1.6.1^65^. This approach allowed us to identify specific cell populations that frequently co-localize with *PGK1*^+^ Tregs, indicating potential interactions within the tissue microenvironment^65^.

### CellScape multiplex immunofluorescence data processing and analysis

The Cellscape platform generated 16-bit OME-TIFF images following a standardized protocol that included image alignment, stitching, and flat-field correction, all performed within the Navigator software. Pixel-wise autofluorescence correction was applied independently for each marker using autofluorescence reference images acquired prior to each imaging cycle. Regions of Interest (ROIs) were manually annotated to include only tissue areas suitable for analysis, excluding artifacts such as folds and antibody aggregates. Cell-level segmentation was adapted from CosMx segmentation, enabling direct comparison of protein and transcriptional features. Cell masks were visually inspected to confirm pixel-level alignment for each tissue. Marker expression features were extracted at the cell level, and automatic thresholding was performed using the Otsu algorithm. Both two-level (negative/positive) and three-level (negative/low/high) thresholds were computed independently for each tissue region. Cells were classified into 79 user-defined phenotypes based on combinatorial marker expression across up to three markers per cell, encompassing immune, lineage, and architecture populations as defined by the Immune Profiling and Architecture VistaPlex kits. Spatial neighborhood analysis was conducted using a k-nearest neighbors (k-NN) approach (*k* = 15) to define 25 neighborhood clusters, integrating both cell-intrinsic and neighborhood phenotypic information.

### Mast cell-Treg experiments

#### Isolation and culture of bone marrow-derived mast cells

Bone marrow-derived mast cells were generated from the bone marrow of C57BL/6 wild-type (WT) mice^66^. Femurs were dissected, and the bone marrow was harvested by flushing the cavity using RPMI-1640 medium. Bone marrow cells were cultured with 15 ng/mL recombinant murine IL-3 in complete RPMI-1640 medium, supplemented with 10% fetal bovine serum (FBS), 1% penicillin-streptomycin, 2  mM L-glutamine, and 50 μM β-mercaptoethanol. Cells were cultured in a humidified incubator at 37 °C with 5% CO₂, with fresh media and cytokines added every 5 days. Bone marrow-derived mast cells were harvested after a 6-week culture. Before use, all the mice experiments were reviewed and approved by the Animal Care and Use Committee of Northwestern University.

#### Isolation of Tregs

Tregs were isolated from spleens of C57BL/6 Foxp3-GFP reporter mice (*n* = 3 male, *n* = 3 female). Total CD4^+^ T cells were purified using BD anti-mouse CD4 Magnetic Particles. Subsequently, live Tregs were sorted by gating on propidium iodide-negative, Foxp3-GFP^+^ cells using a Sony SH800 cell sorter.

#### Co-culture of Tregs and mast cells

Tregs (150 K cells/well) were activated with anti-CD3/28 beads (beads: Tregs =  2:1) in the presence or absence of bone marrow-derived mast cells (300 K cells/well) and anti-IL-6 (10 µg/mL) in 250 µL of complete RPMI-1640 medium, 10% fetal bovine serum (FBS), 1% penicillin-streptomycin, 2 mM L-glutamine, 1 mM sodium pyruvate, and 50 μM β-mercaptoethanol.

Flow cytometry: Cells were harvested and stained with a Fixable Near-IR Dead Cell Stain Kit and anti-mouse CD4 (1:40, Biolegend). After permeabilization and fixation using Foxp3/Transcription Factor Fixation/Permeabilization set, cells were stained with anti-mouse IL-17A (1:40, Biolegend). Finally, cells were collected by BD BD FACSymphony™ A5 SE and BD FACS Diva software and analyzed by FlowJo. The gating strategy is shown in Supplementary Fig. 10a.

### Treg PGK1 experiments

#### Murine Th1, Th2, Th17, and iTregs in vitro differentiation

Spleens were harvested and dissociated in a solution buffer containing 1× PBS with 2% FBS and 1 mM EDTA. The resulting mixture was filtered through a 70 μm sterile mesh filter. Cells were centrifuged at 1500 rpm for 5 min and treated with ACK lysis buffer (0.15 M ammonium chloride, 10 mM potassium biacarbonate, and 0.1 mM EDTA) for 2 min. CD4^+^ total T cells were isolated from the spleens from C57BL/6 J mice by a CD4^+^ T cell negative selection kit (Stem Cell Technologies #19765). Isolated CD4^+^ T cells were subsequently activated by anti-CD3 (3 μg/mL, Thermofisher Scientific#16-0031-86) and anti-CD28 (5 μg/mL, Thermofisher Scientific#16-0281-86) plate-bound antibodies and differentiated using polarizing T cell mediums made of RPMI 1640 medium containing 10% FBS, 1% penicillin/streptomycin, β-mercaptoethanol (50 μM, Thermofisher Scientific#21985023), and 1% L-glutamine (Thermofisher Scientific#25030081) with different conditions for 3 days. For Th1 polarization: IL-2 (5 ng/mL, PeproTech#212-12), IL-12 (5 ng/mL, PeproTech #210-12), and anti-IL-4 (1 μg/mL, Bio X Cell#BE0045); For Th2 polarization: IL-2 (5 ng/mL, PeproTech#212-12) and IL-4 (30 ng/mL, PeproTech#214-14); For Th17 polarization: IL-6 (50 ng/mL, PeproTech#216-16), TGF-β (5 ng/mL, PeproTech#100-21), anti-IFN-γ (2 μg/mL, Bio X Cell# BE0055), and anti-IL-4 (2 μg/mL, Bio X Cell#BE0045); For iTregs polarization: IL-2 (5 ng/mL, PeproTech#212-12), TGF-β (5 ng/mL, PeproTech#100-21), anti-IFN-γ (2 μg/mL, Bio X Cell# BE0055), and anti-IL-4 (2 μg/mL, Bio X Cell#BE0045).

#### Quantitative polymerase chain reaction (PCR)

RNA was extracted using a RNeasy Mini Kit (Qiagen#74106), and RT-PCR was performed following the manufacturer’s protocol using gene-specific primer sets. β-actin (F): 5′-GTGACGTTGACATCCGTAAAGA-3’, β-actin (R): 5′-GCCGGACTCATCGTACTCC-3′; PGK1 (F): 5′- ATGTCGCTTTCCAACAAGCTG-3’, PGK1 (R): 5′- GCTCCATTGTCCAAGCAGAAT-3′. Data were analyzed using the comparative Ct method (ΔΔCt method).

#### Flow cytometry

Cells were collected, washed twice with PBS, and centrifuged at 350 rcf for 5 min. Cells were then stained with Fixable Viability Dye 450 (Thermofisher Scientific, 65-0863-18) at 1:1000 in PBS for 10 min at 4 °C. After washing with FACS buffer (3% FBS in PBS), cells were stained with surface markers, including CD4 (GK1.5) and CD25 (PC61) for 20 min at 4 °C. For the detection of intracellular proteins, the FoxP3/Transcription Factor Staining Buffer Set (Thermofisher Scientific#00-5223-56) was used. Cells were stained according to the manufacturer’s recommendation after cell surface stain. Intracellular antibodies included FoxP3 (FJK-16s) and ROR-γt (Q31-378). All analyzes were performed on a BD LSRFortessa X-20 Analyzer. The gating strategies are shown in Supplementary Fig. 10b, c.

### Reporting summary

Further information on research design is available in the Nature Portfolio Reporting Summary linked to this article.

## Supplementary information

Supplementary Information

Description of Additional Supplementary Files

Supplementary Dataset 1

Supplementary Dataset 2

Supplementary Dataset 3

Supplementary Dataset 4

Supplementary Dataset 5

Supplementary Dataset 6

Supplementary Dataset 7

Supplementary Dataset 8

Supplementary Dataset 9

Supplementary Dataset 10

Reporting Summary

Transparent Peer Review file

## Source data

Source Data

## Data Availability

The comparative dataset generated and analyzed in this study have been deposited in NCBI’s Gene Expression Omnibus database under accession codes GSE312415 and GSE312420. The Crohn’s & Colitis Foundation data used in this study are available upon approved application to Crohn’s & Colitis Foundation IBD Plexus (https://www.crohnscolitisfoundation.org/ibd-plexus). The CellScape protein expression data and the corresponding CosMx Seurat object are available via Figshare (10.6084/m9.figshare.31595527). Source data are provided with this paper and available via Figshare (10.6084/m9.figshare.31751848). The processed data presented in graphs are provided in the Source Data file. The scRNA-seq reference data used for cell type annotation in this study are available in the Gut Cell Atlas (https://www.gutcellatlas.org/), the Single Cell Portal under accession code SCP259, and NCBI’s Gene Expression Omnibus database under accession code GSE232217. Source data are provided with this paper.

## Code availability

Custom scripts for data processing and quality evaluation are available at https://zenodo.org/records/19006691.
