# Supplementary material for: Spatial transcriptomics atlas of inflammatory bowel disease to guide implementation in research consortiums and clinical trials
Source: Nat Commun. 2026 Apr 28;17:5808. doi: 10.1038/s41467-026-72482-w (PMC13328602; doi:10.1038/s41467-026-72482-w)
Supplement: Supplementary file 1 — Supplementary Information [file 41467_2026_72482_MOESM1_ESM.pdf]

Supplementary Information for "Spatial transcriptomics atlas of inflammatory bowel disease to guide implementation in research consortiums and clinical trials"

Yiming Li[1], Cenfu Wei[2], Wenjing Yang[2], Hai Wang[2], Alan Robinson[2], Isabella Peshek[2], Tianming Yu[2], Jiwoon Park[3,4], Jenny Yanyi Ding[1], Stephen B. Hanauer[2], Emanuelle Bellaguarda[2], Laura Yun[2], Ronen Sumagin[5], Guang-Yu Yang[5], Christopher E. Mason[3,4,6], James D. Lewis[7], Deyu Fang[5,8], Yingzi Cong[2,5,8,9], Yuan Luo[1,10,11*], Parambir S. Dulai[2,8*]

[1]Department of Preventive Medicine, Northwestern University, Chicago, IL, USA

[2]Department of Medicine, Division of Gastroenterology and Hepatology, Northwestern University, Chicago, IL, USA

[3]Department of Systems and Computational Biomedicine, Weill Cornell Medicine, New York, NY, USA

[4]The HRH Prince Alwaleed Bin Talal Bin Abdulaziz Alsaud Institute for Computational Biomedicine, Weill Cornell Medicine, New York, NY, USA

[5]Department of Pathology, Northwestern University, Chicago, IL, USA

[6]The Feil Family Brain and Mind Research Institute, Weill Cornell Medicine, New York, NY, USA

[7]Division of Gastroenterology and Hepatology, Perelman School of Medicine, University of Pennsylvania, Philadelphia, PA, USA

[8]Center for Human Immunobiology, Northwestern University, Chicago, IL, USA

[9]Department of Microbiology and Immunology, Northwestern University, Chicago, IL, USA

[10]Northwestern University Clinical and Translational Sciences Institute, Northwestern University, Chicago, IL, USA

[11]Center for Collaborative AI in Healthcare, Institute for AI in Medicine, Northwestern University, Chicago, IL, USA

[*]Co-corresponding author: Yuan Luo (yuan.luo@northwestern.edu), Parambir S. Dulai, MD (parambir.dulai@northwestern.edu)

Supplementary Figures

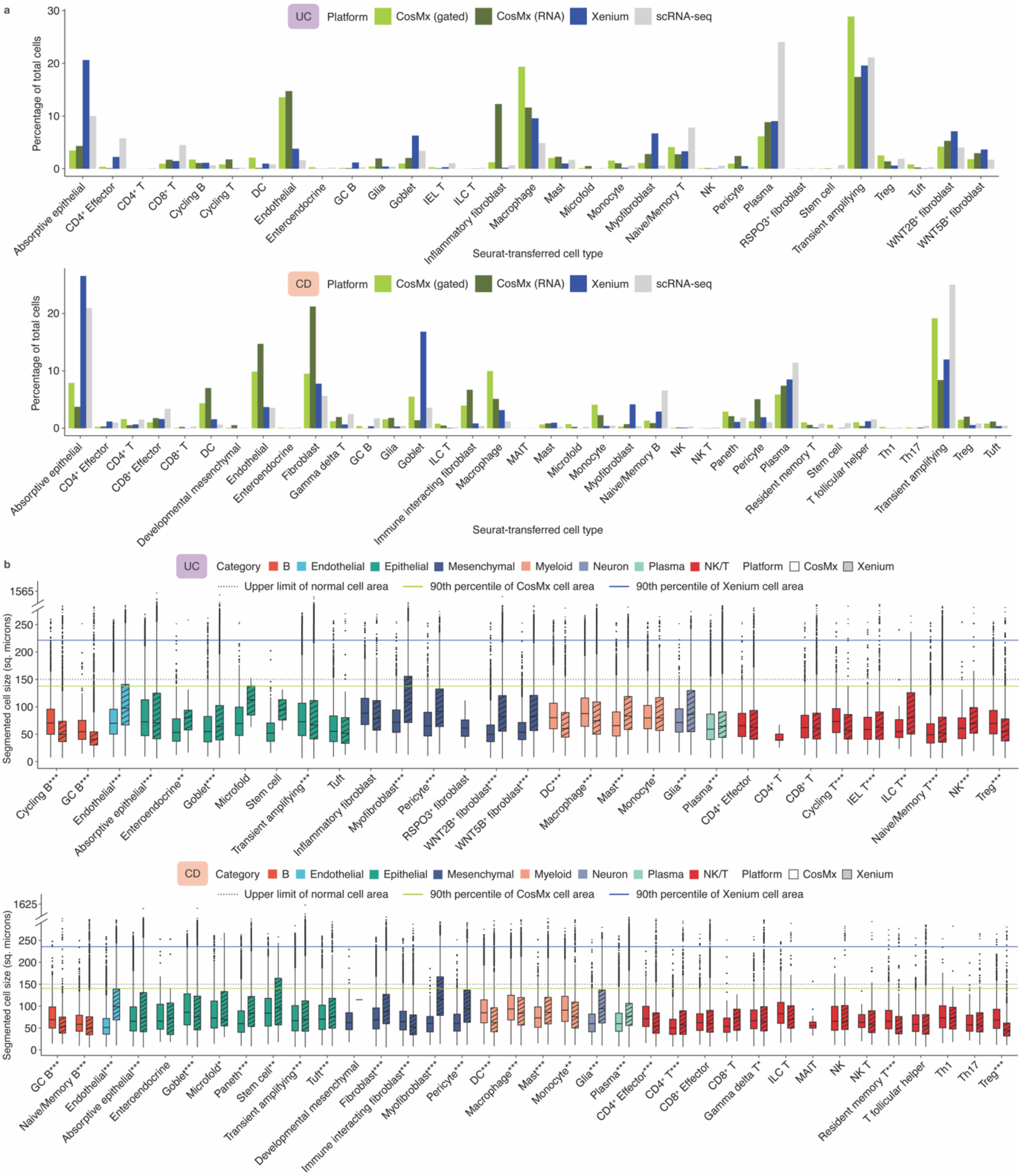

Supplementary Fig. 1: Cell type proportions and cell area distributions across different cell types in CosMx multi-tissue and Xenium multi-tissue data. a, Predicted cell type proportions using CosMx (protein-gated), CosMx (only RNA-based), Xenium, and scRNA-seq data in ulcerative colitis and Crohn's disease data. **b**, Cell areas across different predicted cell types based on CosMx (protein-gated; n = 197,541 cells for ulcerative colitis and n =

126,908 cells for Crohn's disease) and Xenium (n = 211,335 cells for ulcerative colitis and n = 157,996 cells for Crohn's disease) data. The boxplots are colored by cell type category, and display medians and quartiles, with whiskers extending to 1.5 times the interquartile range. The upper limit of normal cell area (150 squared microns) as well as the 90th percentiles of CosMx and Xenium cell areas are shown as horizontal lines. Two-sided Wilcoxon rank-sum tests are performed to examine whether CosMx and Xenium cell areas of the cell type are significantly different. All the p-values are adjusted for multiple testing using the false discovery approach. *, adjusted p-value < 0.05; **, adjusted p-value < 0.01; ***, adjusted p-value < 0.001; CD, Crohn's disease; DC, dendritic cells; GC, germinal center; IEL, intraepithelial lymphocytes; ILC, innate lymphoid cells; MAIT, mucosal-associated invariant T cells; NK, natural killer cells; RNA, ribonucleic acid; scRNA-seq, single-cell RNA sequencing; sq., squared; UC, ulcerative colitis.

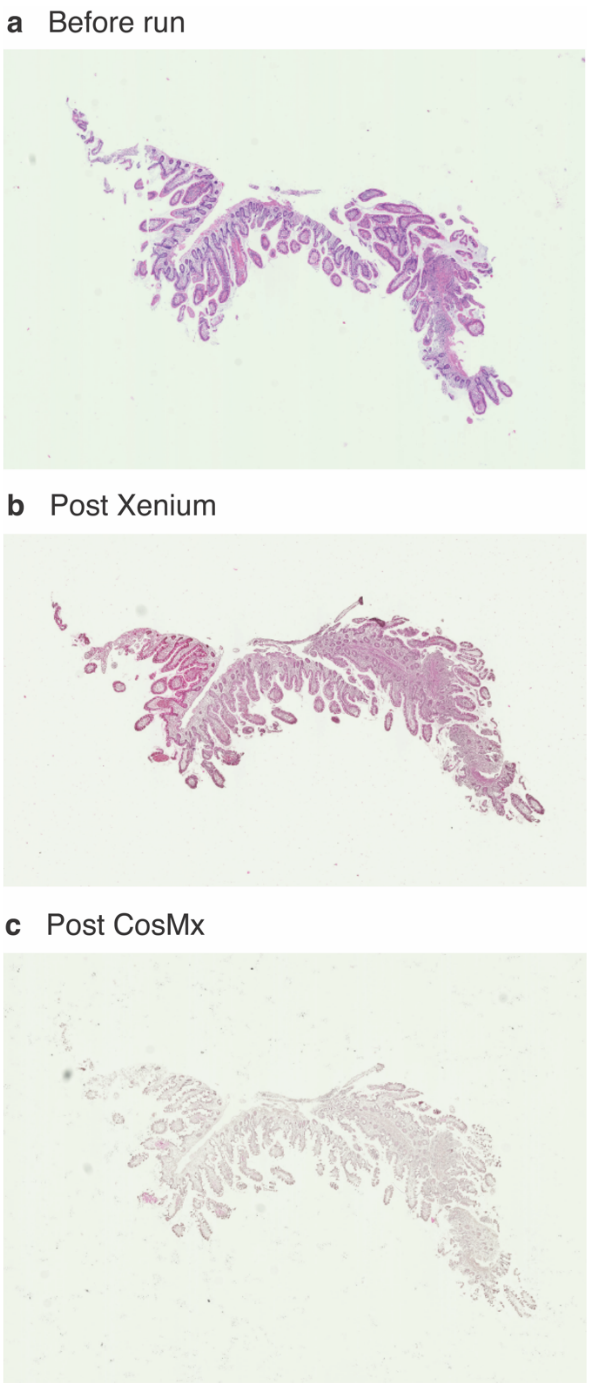

Supplementary Fig. 2: Example H&E images before and post run. Both platforms demonstrated intact tissue architecture post-run, with better staining observed for Xenium.

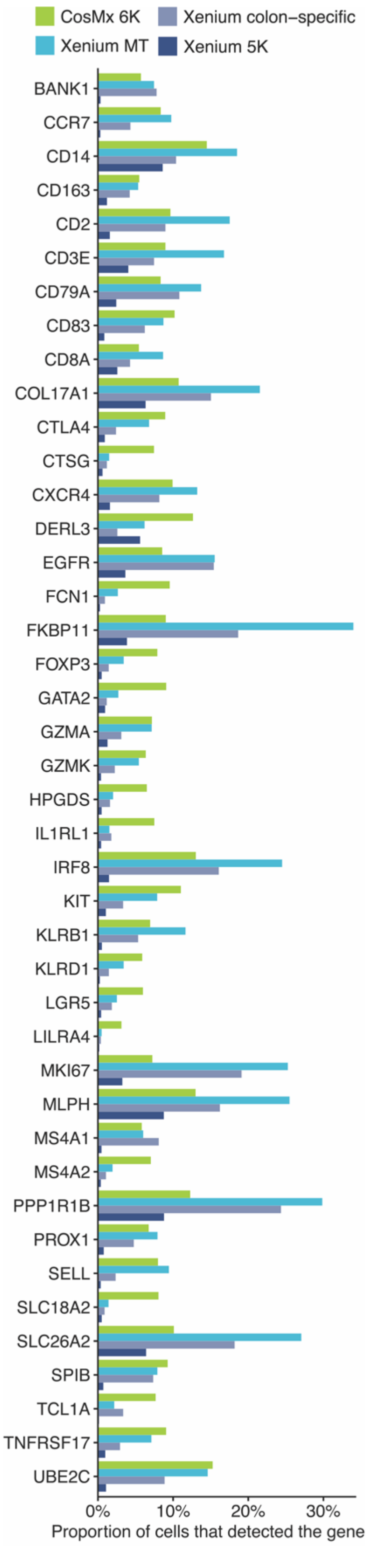

Supplementary Fig. 3: Detection rate of the overlapping genes across CosMx 6K, Xenium multi-tissue, Xenium colon-specific, and Xenium 5K. The Xenium 5K panel has the lowest sensitivity across all compared panels (CosMx 6K: n = 90,470 cells, Xenium

multi-tissue: n = 135,360 cells, Xenium 5K: n = 147,287 cells, Xenium colon-specific: n = 111,933 cells). MT, multi-tissue.

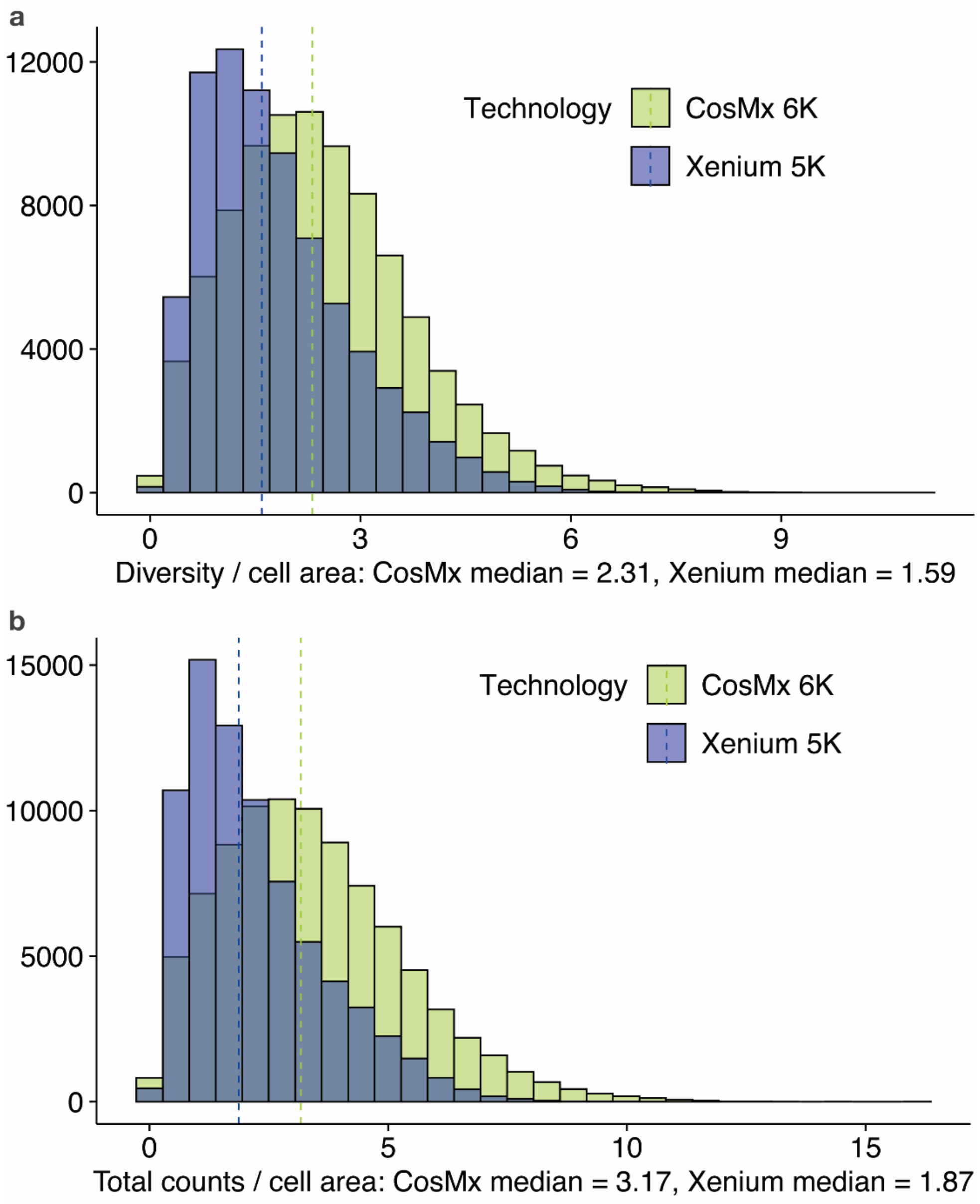

Supplementary Fig. 4: Difference in overlapping genes diversity and total counts across CosMx 6K and Xenium 5K data. CosMx 6K data (n = 89,058 cells) has higher transcript diversity and total counts than Xenium 5K data (n = 75,389 cells). The CosMx and Xenium medians are shown in vertical dashed lines.

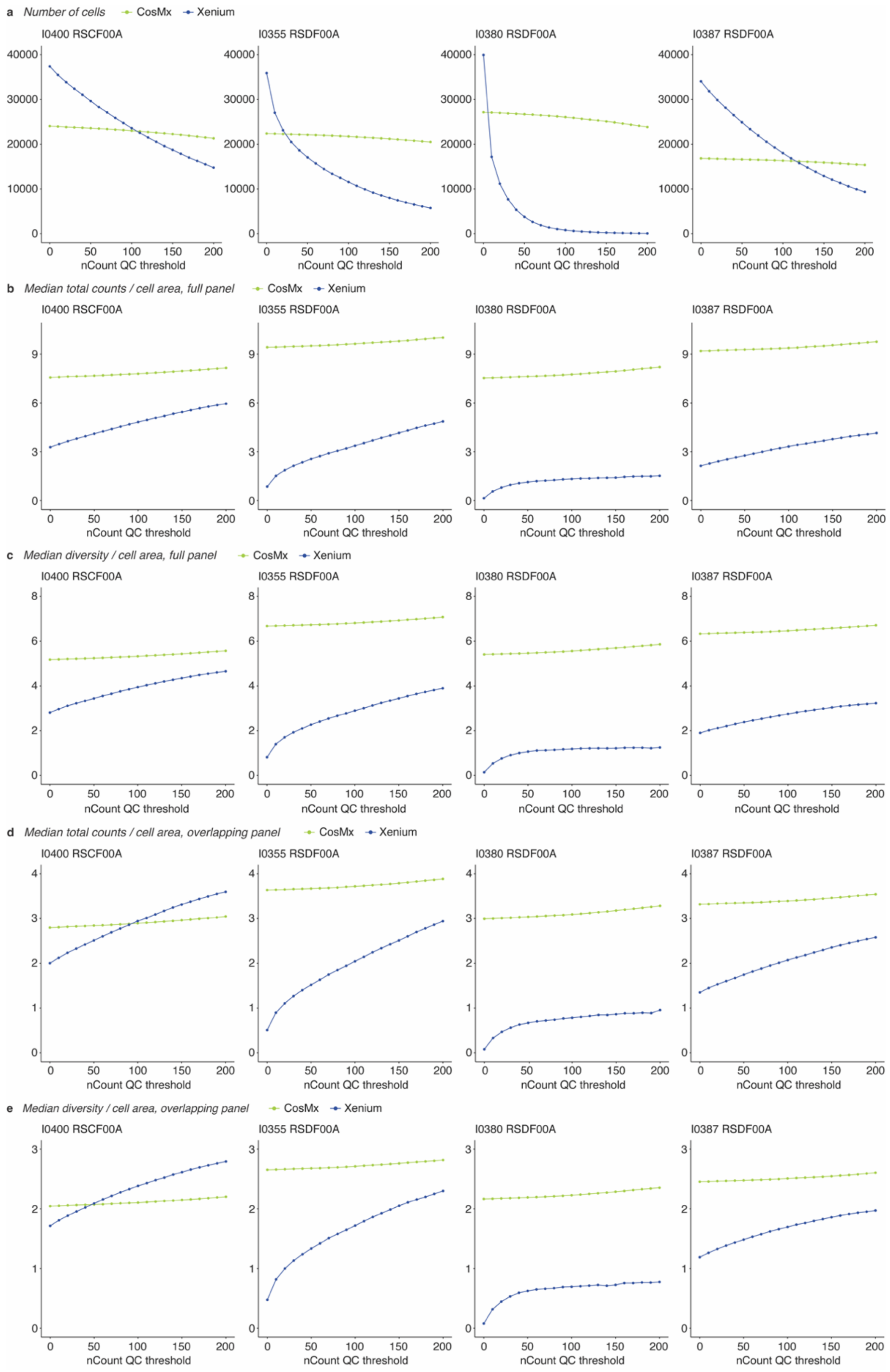

Supplementary Fig. 5: Before and after quality control cell counts and data quality in CosMx 6K and Xenium 5K data. The numbers of cells are shown in panel (a). nCount, total counts of unique molecular identifiers; QC, quality control.

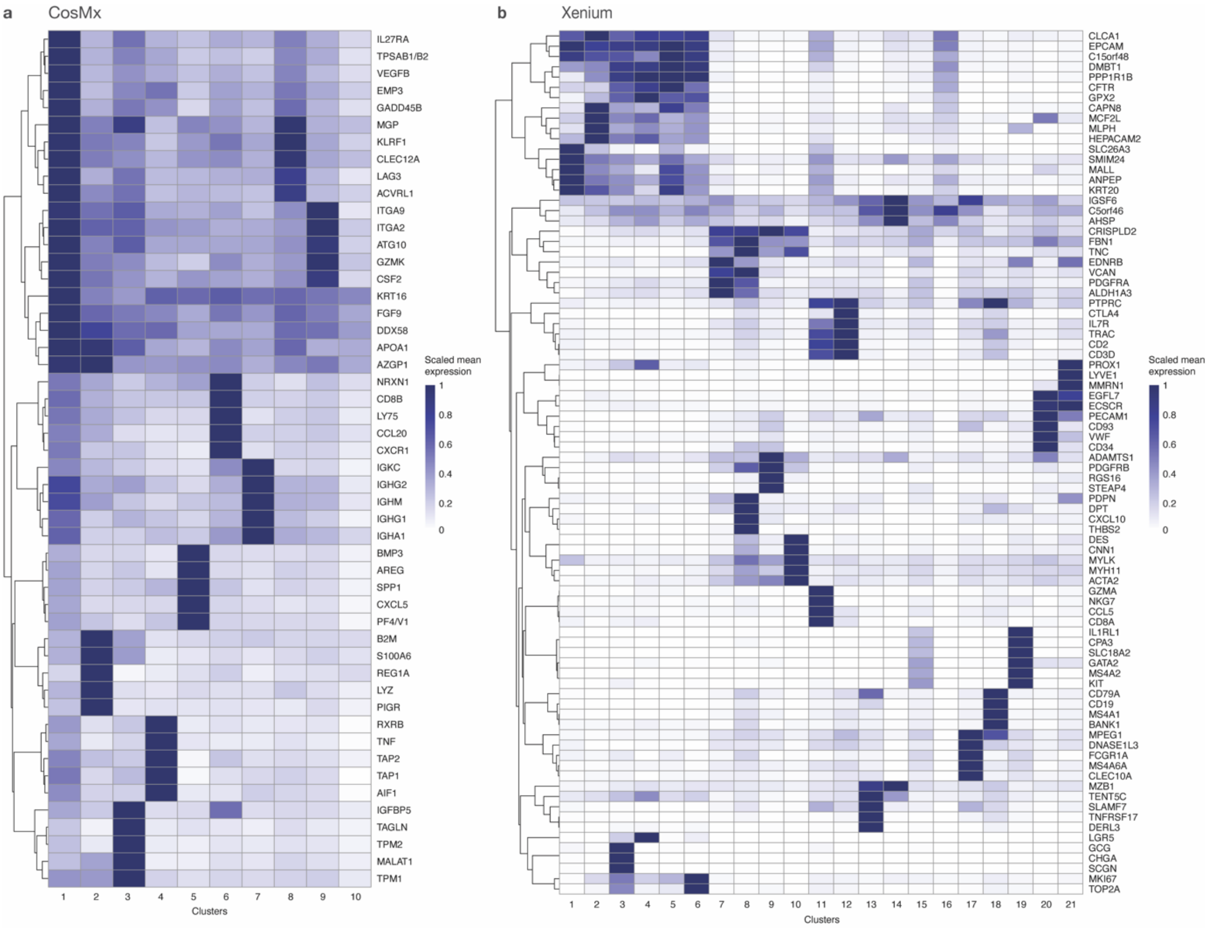

Supplementary Fig. 6: Top marker genes in different clusters of CosMx multi-tissue (n = 18,756 cells) **and Xenium multi-tissue** (n = 19,289 cells) **data from an inflamed ileal Crohn's disease sample.** Differential gene expression analysis was performed using two-sided Wilcoxon rank-sum tests. The top five genes in each cluster with an average log fold change above 0.5 are selected. The tiles are colored by the scaled mean expression of the considered gene (row) in each cluster (column).

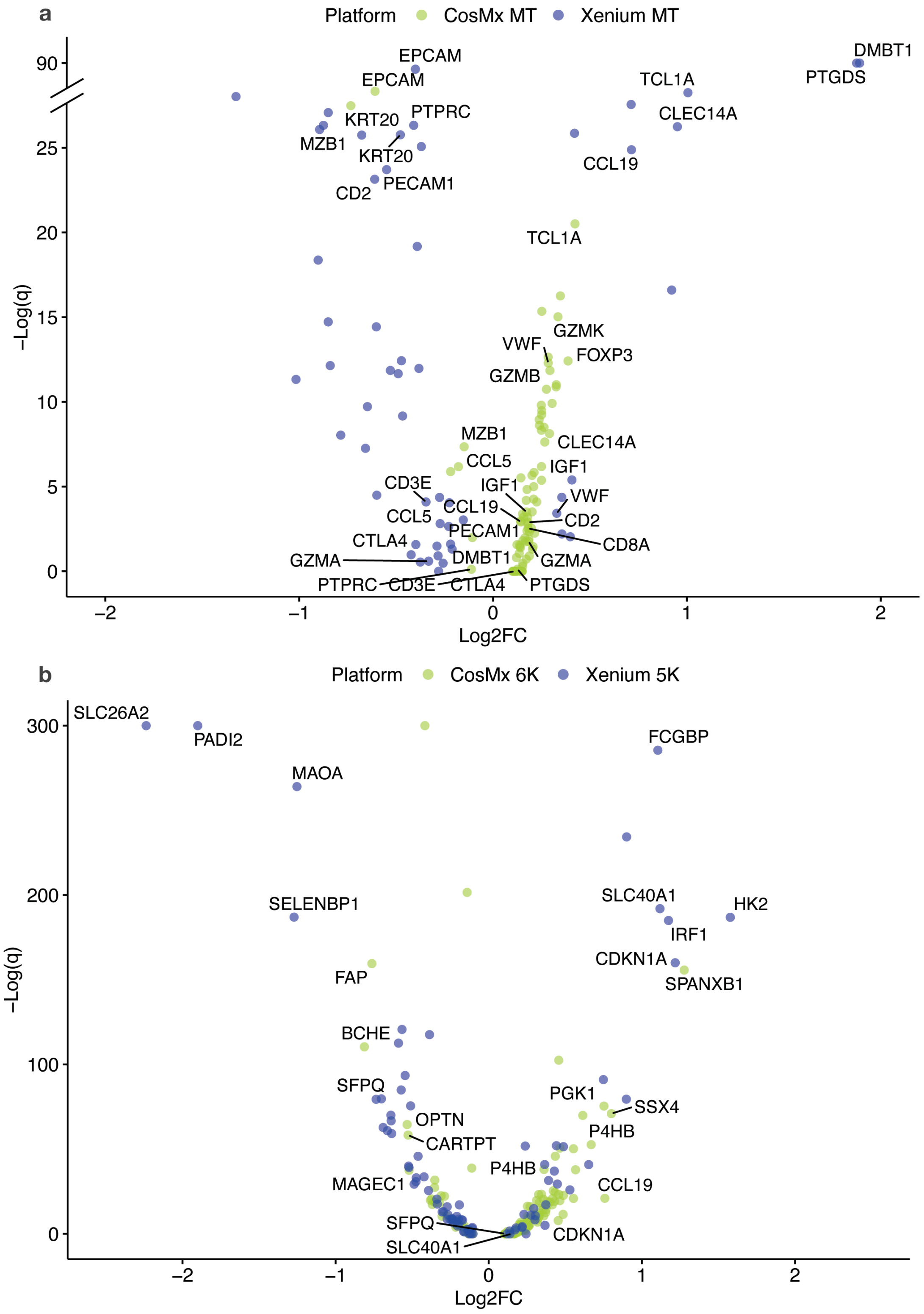

Supplementary Fig. 7: Extended figures for (a) Fig. 2b and (b) Fig. 3c. a, Volcano plot of the genes differentially expressed in inflamed ileal CD compared to others in CosMx multi-tissue and Xenium multi-tissue data. FOXP3 is identified as an up-regulated gene in inflamed

ileal CD by CosMx data (n = 34,096 inflamed ileal CD cells versus 290,353 other cells) but not Xenium data (n = 24,427 inflamed ileal CD cells versus 344,904 other cells). The visualization is limited to the 119 overlapping genes between the two panels. Known T-cell-related genes and the top genes from each technology are labelled. The points are colored by technology (green – CosMX, blue – Xenium). **b**, Volcano plot of the genes differentially expressed in inflamed UC and healthy colon in CosMx 6K and Xenium 5K data. PGK1 is identified as an up-regulated gene in inflamed UC by CosMx data (n = 43,347 inflamed UC cells versus 23,596 healthy colon cells) but not Xenium data (n = 28,678 inflamed UC cells versus 29,646 healthy colon cells). The visualization is limited to the 2,552 overlapping genes between the two panels. The top genes from each technology are labeled. The points are colored by technology (green – CosMX, blue – Xenium).

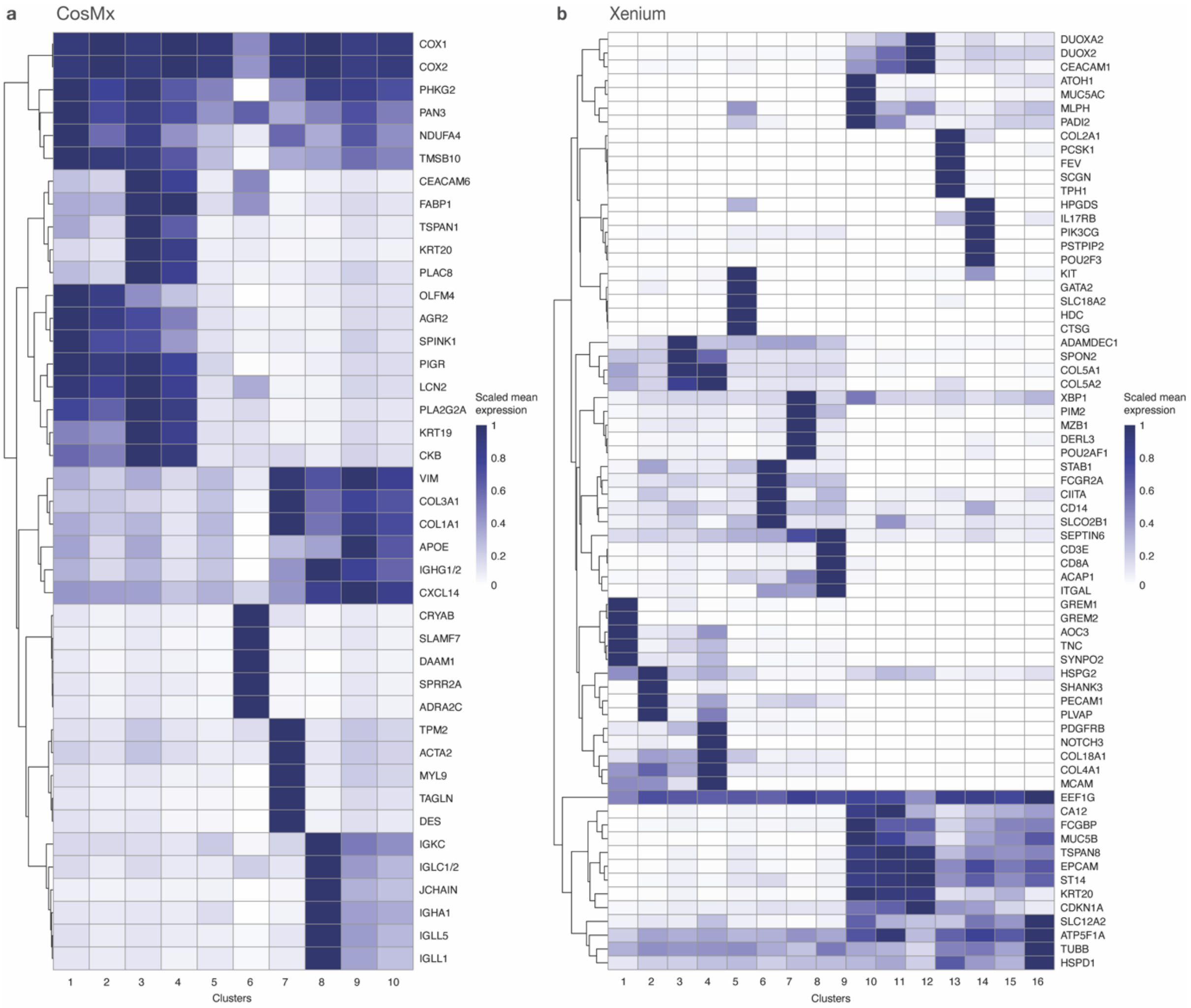

Supplementary Fig. 8: Top marker genes in different clusters of CosMx 6K (n = 16,627 cells) **and Xenium 5K** (n = 24,911 cells) **data from an inflamed ulcerative colitis sample.** Differential gene expression analysis was performed using two-sided Wilcoxon rank-sum tests. The top five genes in each cluster with an average log fold change above 0.5 are selected. The tiles are colored by the scaled mean expression of the considered gene (row) in each cluster (column).

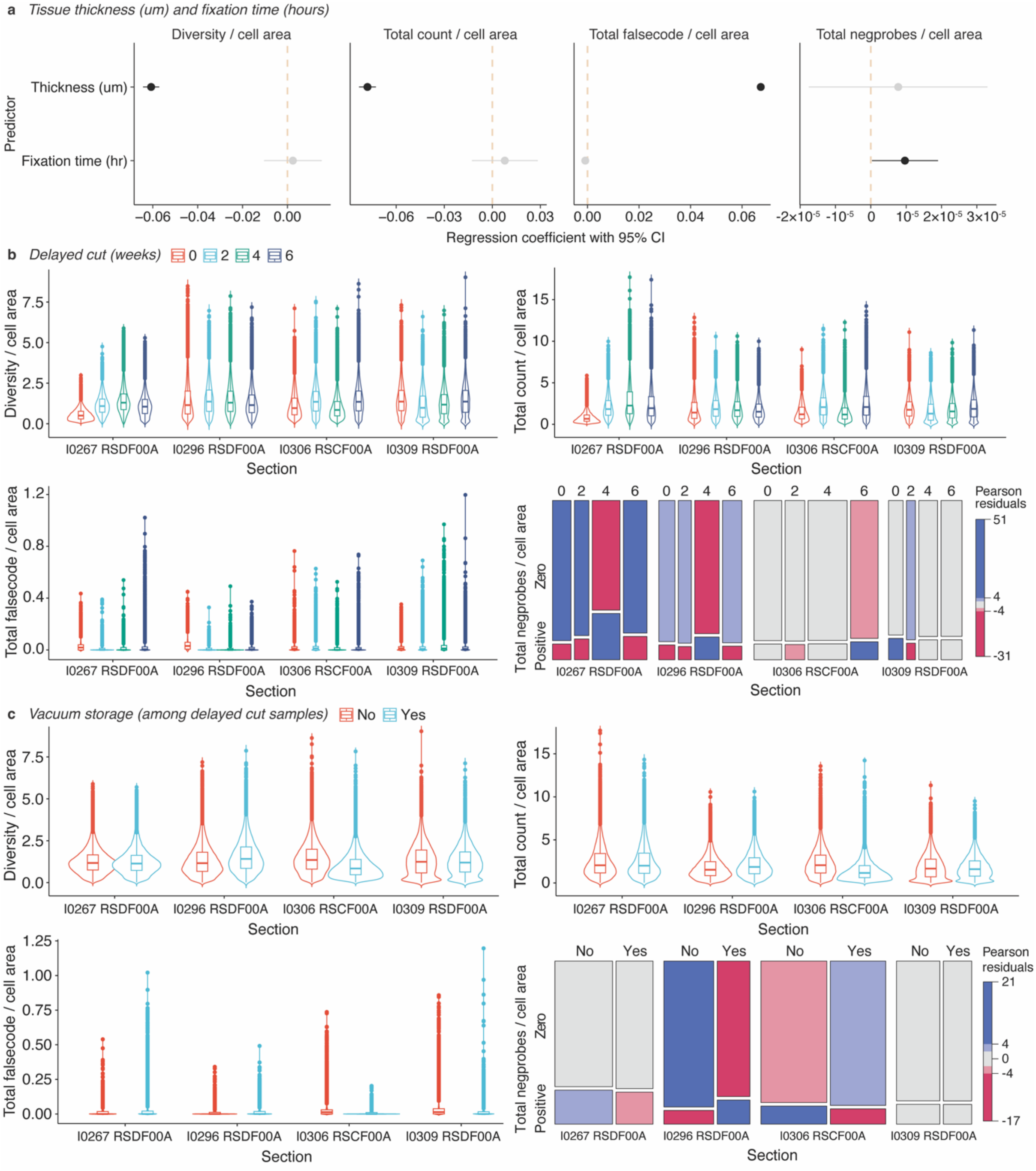

Supplementary Fig. 9: The effect of sample preparation condition on CosMx data quality. Sample preparation conditions including **a**, tissue thickness and fixation time, **b**, delayed cut, and **c**, vacuum storage influence transcript diversity, total count, total falsecode, and total negprobes by cell area differently (n = 355,733 cells). **a**, A greater tissue thickness is shown to be significantly associated with lower diversity and total counts as well as higher total falsecode, whereas a longer fixation time is significantly associated with higher total

negprobes. The regression coefficients with 95% confidence intervals are shown, and the insignificant (adjusted p-value ≥ 0.05) coefficients are colored gray. On the other hand, **b**, delayed cut time, and **c**, vacuum storage show no clear and consistent impact on data quality. The boxplots display medians and quartiles, with whiskers extending to 1.5 times the interquartile range, while the violin plot outlines represent the kernel probability density. The mosaic plots visualize the binarized total negprobes by sample category contingency tables, in which each block's size is proportional to the cell counts in that category. The blocks are colored by the Pearson residuals. Red indicates there are fewer observations than expected, blue indicates there are more observations than expected, and gray indicates insignificance (adjusted p-value ≥ 0.05). CI, confidence interval.

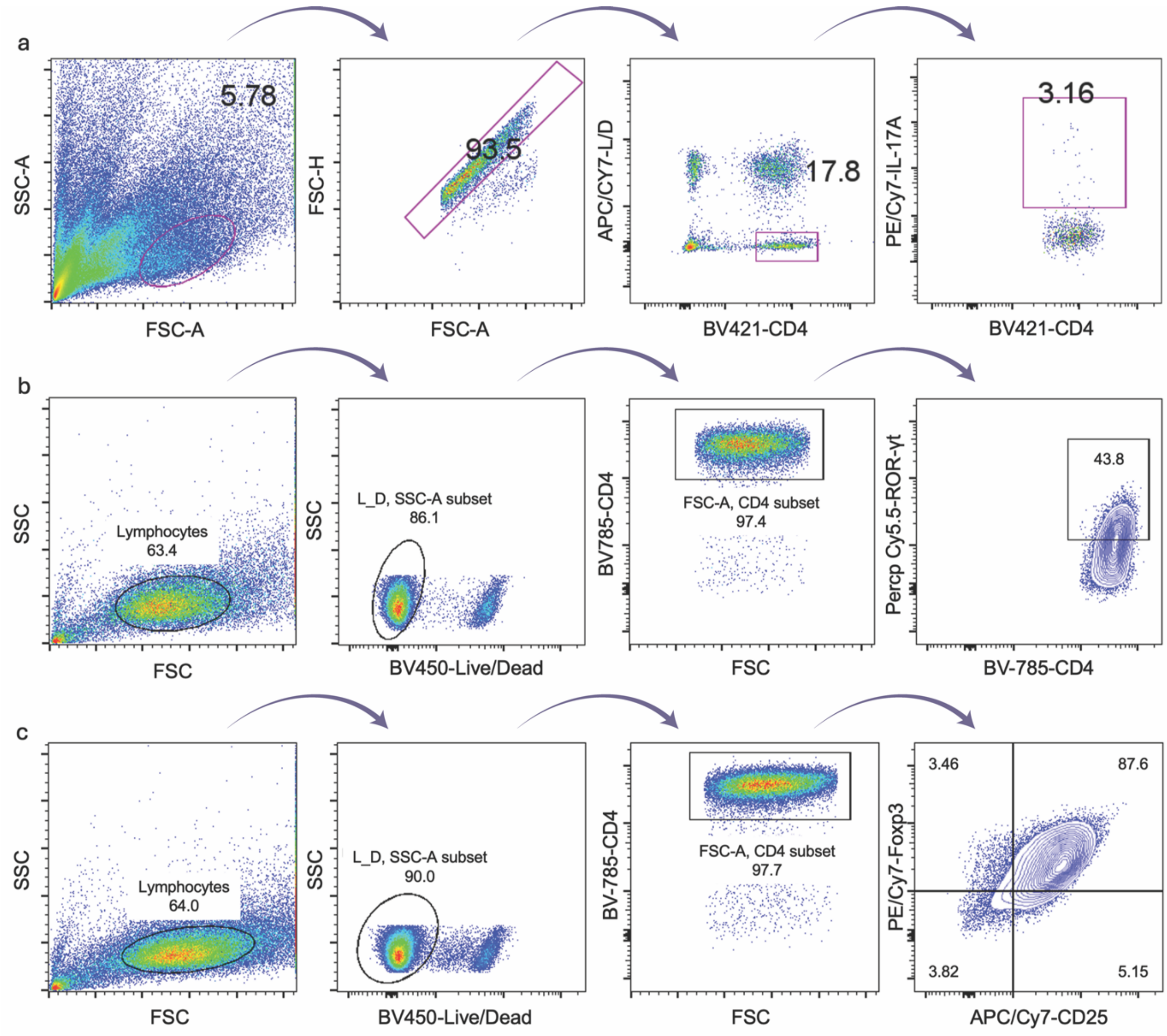

Supplementary Fig. 10: Gating strategy of the flow cytometry experiments. The gating strategy for **a**, IL-17A$^+$ CD4$^+$ T cells (Fig. 4f), **b**, ROR-γt$^+$ CD4$^+$ T cells (Fig. 5g), and **c**, CD25$^+$ Foxp3$^+$ cells (Fig. 5j) are shown. FSC, forward scatter; SSC, side scatter.
