# Supplementary material for: Spatial transcriptomics atlas of inflammatory bowel disease to guide implementation in research consortiums and clinical trials
Source: Nat Commun. 2026 Apr 28;17:5808. doi: 10.1038/s41467-026-72482-w (PMC13328602; doi:10.1038/s41467-026-72482-w)
Supplement: Supplementary file 2 — Description of Additional Supplementary Files [file 41467_2026_72482_MOESM2_ESM.pdf]

Description of Additional Supplementary Files

Supplementary Data 1: List of overlapping genes across different CosMx and Xenium panels. The compared panels include CosMx multi-tissue, Xenium multi-tissue, Xenium colon-specific, CosMx 6K, and Xenium 5K.

Supplementary Data 2: Q scores for Xenium across samples for multi-tissue and 5K panels. Q-score estimates are provided as means and medians for each panel for each sample.

Supplementary Data 3: The effect of platform on data quality. The effect of different platforms used (CosMx and Xenium platforms) on cell level quality metrics was analyzed using linear mixed models with sample key as the random effect. The estimated fixed effects and the number of observations used in each set of analyses are shown.

Supplementary Data 4: Before and after quality control cell counts and comparative performance for CosMx multi-tissue, Xenium multi-tissue, CosMx 6K, and Xenium 5K data. Cell-level quality control was performed based on the total gene counts detected within each cell.

Supplementary Data 5: Before and after quality control cell counts and comparative performance for CosMx and Xenium data segmented using different cell segmentation methods. Cell level quality control was performed based on the total gene counts detected within each cell.

Supplementary Data 6: Differential gene expression results in CosMx and Xenium data. The gene expressions in cells of one sample category (tissue type, disease type, and disease state) are compared with those in other cells using two-sided Wilcoxon rank sum tests. The average log fold changes, unadjusted p-values, and p values after adjusting for multiple testing are listed.

Supplementary Data 7: Per gene nuclear and non-nuclear detection for Xenium and CosMx, and per-gene Q scores for Xenium across samples for multi-tissue and 5K panels. Nuclear and non nuclear detection rate are reported as proportions. Q scores are reported as mean, median, min, max, standard deviations, and interquartile ranges per gene per sample.

Supplementary Data 8: Cell type specific Q-scores for PGK1 in Xenium 5K data. Q scores are reported as mean, median, min, max, per sample.

Supplementary Data 9: Seurat prediction scores across all panels, separated by major and minor cell types. Presented as means +/- standard deviations, for all panels with varying filtering and/or gating strategies.

Supplementary Data 10: Blinded GI pathologist morphology evaluation of biopsies. The biopsies corresponded to CosMx and Xenium runs.
