# Supplementary material for: Spatial transcriptomics atlas of inflammatory bowel disease to guide implementation in research consortiums and clinical trials
Source: Nat Commun. 2026 Apr 28;17:5808. doi: 10.1038/s41467-026-72482-w (PMC13328602; doi:10.1038/s41467-026-72482-w)
Supplement: Supplementary file 13 — Reporting Summary [file 41467_2026_72482_MOESM13_ESM.pdf]

nature portfolio

Corresponding author(s): Parambir Dulai and Yuan Luo

Last updated by author(s): Mar 16, 2026

Reporting Summary

Nature Portfolio wishes to improve the reproducibility of the work that we publish. This form provides structure for consistency and transparency in reporting. For further information on Nature Portfolio policies, see our Editorial Policies and the Editorial Policy Checklist.

Statistics

For all statistical analyses, confirm that the following items are present in the figure legend, table legend, main text, or Methods section.

n/a	Confirmed	
☐	☒	The exact sample size (*n*) for each experimental group/condition, given as a discrete number and unit of measurement
☐	☒	A statement on whether measurements were taken from distinct samples or whether the same sample was measured repeatedly
☐	☒	The statistical test(s) used AND whether they are one- or two-sided<br>*Only common tests should be described solely by name; describe more complex techniques in the Methods section.*
☐	☒	A description of all covariates tested
☐	☒	A description of any assumptions or corrections, such as tests of normality and adjustment for multiple comparisons
☐	☒	A full description of the statistical parameters including central tendency (e.g. means) or other basic estimates (e.g. regression coefficient) AND variation (e.g. standard deviation) or associated estimates of uncertainty (e.g. confidence intervals)
☐	☒	For null hypothesis testing, the test statistic (e.g. *F*, *t*, *r*) with confidence intervals, effect sizes, degrees of freedom and *P* value noted<br>*Give P values as exact values whenever suitable.*
☒	☐	For Bayesian analysis, information on the choice of priors and Markov chain Monte Carlo settings
☒	☐	For hierarchical and complex designs, identification of the appropriate level for tests and full reporting of outcomes
☐	☒	Estimates of effect sizes (e.g. Cohen's *d*, Pearson's *r*), indicating how they were calculated

Our web collection on statistics for biologists contains articles on many of the points above.

Software and code

Policy information about availability of computer code

Data collection	The comparative dataset generated and analyzed in this study have been deposited in NCBI's Gene Expression Omnibus database under accession codes GSE312415 (https://www.ncbi.nlm.nih.gov/geo/query/acc.cgi?acc=GSE312415) and GSE312420 (https://www.ncbi.nlm.nih.gov/geo/query/acc.cgi?acc=GSE312420). The Crohn's & Colitis Foundation data used in this study are available upon approved application to Crohn's & Colitis Foundation IBD Plexus (https://www.crohnscolitisfoundation.org/ibd-plexus). The CellScape protein expression data and the corresponding CosMx Seurat object are available via Figshare (https://doi.org/10.6084/m9.figshare.31595527). Source data are provided with this paper and available via Figshare (https://doi.org/10.6084/m9.figshare.31751848). The processed data presented in graphs are provided in the Source Data file. The scRNA-seq reference data used for cell type annotation in this study are available in the Gut Cell Atlas (https://www.gutcellatlas.org/), the Single Cell Portal under accession code SCP259 (https://singlecell.broadinstitute.org/single_cell/study/SCP259/intra-and-inter-cellular-rewiring-of-the-human-colon-during-ulcerative-colitis), and NCBI's Gene Expression Omnibus database under accession code GSE232217 (https://www.ncbi.nlm.nih.gov/geo/query/acc.cgi?acc=GSE232217).
Data analysis	Custom scripts for data processing and quality evaluation are available at https://zenodo.org/records/19006691.

For manuscripts utilizing custom algorithms or software that are central to the research but not yet described in published literature, software must be made available to editors and reviewers. We strongly encourage code deposition in a community repository (e.g. GitHub). See the Nature Portfolio guidelines for submitting code & software for further information.

Data

Policy information about availability of data

All manuscripts must include a data availability statement. This statement should provide the following information, where applicable:

- Accession codes, unique identifiers, or web links for publicly available datasets
- A description of any restrictions on data availability
- For clinical datasets or third party data, please ensure that the statement adheres to our policy

The comparative dataset generated and analyzed in this study have been deposited in NCBI's Gene Expression Omnibus database under accession codes GSE312415 (https://www.ncbi.nlm.nih.gov/geo/query/acc.cgi?acc=GSE312415) and GSE312420 (https://www.ncbi.nlm.nih.gov/geo/query/acc.cgi?acc=GSE312420). The Crohn's & Colitis Foundation data used in this study are available upon approved application to Crohn's & Colitis Foundation IBD Plexus (https://www.crohnscolitisfoundation.org/ibd-plexus). The CellScape protein expression data and the corresponding CosMx Seurat object are available via Figshare (https://doi.org/10.6084/m9.figshare.31595527). Source data are provided with this paper and available via Figshare (https://doi.org/10.6084/m9.figshare.31751848). The processed data presented in graphs are provided in the Source Data file. The scRNA-seq reference data used for cell type annotation in this study are available in the Gut Cell Atlas (https://www.gutcellatlas.org/), the Single Cell Portal under accession code SCP259 (https://singlecell.broadinstitute.org/single_cell/study/SCP259/intra-and-inter-cellular-rewiring-of-the-human-colon-during-ulcerative-colitis), and NCBI's Gene Expression Omnibus database under accession code GSE232217 (https://www.ncbi.nlm.nih.gov/geo/query/acc.cgi?acc=GSE232217).

Research involving human participants, their data, or biological material

Policy information about studies with human participants or human data. See also policy information about sex, gender (identity/presentation), and sexual orientation and race, ethnicity and racism.

Reporting on sex and gender	Due to patient confidentiality, all samples were fully de-identified and sex/gender are not reported. Sex and gender were not considered when selecting samples or recruitment of subjects.
Reporting on race, ethnicity, or other socially relevant groupings	Due to patient confidentiality, all samples were fully de-identified and race/ethnicity or other socially relevant groupings are not reported. race/ethnicity or other socially relevant groupings were not considered when selecting samples or recruitment of subjects.
Population characteristics	Adult IBD patients aged 18-85 years
Recruitment	Detailed in the methods section: The Northwestern DHF IBD Biorepository is a fully de-identified prospective cohort of IBD patients recruited at Northwestern Medicine, and samples were collected and processed prospectively in a systematic and uniform approach to maintain consistent sample quality and comparability. FFPE blocks were obtained through the Northwestern Digestive Health Foundation (DHF) IBD Biorepository (IRB: STU00203172; Supplementary Table 10). IBD patients are consented in clinic and/or endoscopy for the collection, use, and/or sharing of data or samples from the procedure. A standardized biopsy collection protocol is used with routine collection from the ileum, right colon (cecum/ascending), left colon (descending/sigmoid colon), and rectum. Biopsies are targeted for the most inflamed portion of each segment, and if no inflammation is present then they are taken from areas adjacent to where routine care biopsies are taken to most closely align with routine care histology assessments. Disease activity is measured using standardized endoscopic indices for Crohn's disease (Simple Endoscopic Score for Crohn's Disease) and ulcerative colitis (Mayo endoscopic subscore).<br>.<br>The CCF IBD Plexus program Biorepository is a multi-center collaboration, and the IBD Plexus program samples were obtained from the Study of a Prospective Adult Research Cohort with IBD (SPARC IBD), a component of the Crohn's & Colitis Foundation IBD Plexus program research platform. The samples were collected and processed prospectively as outlined in prior work. Given the multi-center nature of the study, FFPE blocks from the CCF IBD Plexus program Biorepository had natural variation in biopsy size, processing time, shipping, and/or fixation times. These samples therefore offered a representation of expected performance for platforms within multi-center consortiums or clinical trials. A range of tissue types and disease states were selected for the study (inflamed or non-inflamed ileum CD, inflamed rectum UC; n = 8 FFPE blocks, 1-2 biopsies per FFPE block).
Ethics oversight	Our study complies with all relevant ethical regulations. FFPE samples were obtained through the Northwestern Digestive Health Foundation (DHF) IBD Biorepository (Northwestern University IRB: STU00203172) as well as through the centrally stored CCF IBD Plexus program Biorepository (Project #1300564; Central IRB). All donors provided written informed consent for use of samples, sharing of data, and appropriate IRB approvals were obtained prior to conduct of experiments.

Note that full information on the approval of the study protocol must also be provided in the manuscript.

Field-specific reporting

Please select the one below that is the best fit for your research. If you are not sure, read the appropriate sections before making your selection.

☒ Life sciences ☐ Behavioural & social sciences ☐ Ecological, evolutionary & environmental sciences

For a reference copy of the document with all sections, see nature.com/documents/nr-reporting-summary-flat.pdf

Life sciences study design

All studies must disclose on these points even when the disclosure is negative.

Sample size	Chosen based on cohort size, disease severity ranges, and power calculations are reported
Data exclusions	none
Replication	Each patient had biological replicates in each FFPE block, 2 biopsies per block
Randomization	none; samples were provided at random to both groups conducting experiments on Xenium and CosMx
Blinding	none

Reporting for specific materials, systems and methods

We require information from authors about some types of materials, experimental systems and methods used in many studies. Here, indicate whether each material, system or method listed is relevant to your study. If you are not sure if a list item applies to your research, read the appropriate section before selecting a response.

Materials & experimental systems

n/a	Involved in the study	
☐	☒	Antibodies
☒	☐	Eukaryotic cell lines
☒	☐	Palaeontology and archaeology
☐	☒	Animals and other organisms
☒	☐	Clinical data
☒	☐	Dual use research of concern
☒	☐	Plants

Methods

n/a	Involved in the study	
☒	☐	ChIP-seq
☐	☒	Flow cytometry
☒	☐	MRI-based neuroimaging

Antibodies

Antibodies used	FACS antibodies: BV421-CD4(Clone# RM4-5; Cat# 100544, Biolegend); PE/Cy7-IL-17A (Clone# TC11-18H10.1, Cat# 506922, Biolegend), BV785-CD4 (Clone# GK1.5; Cat# 100453, Biolegend), APC/CY7-CD25 (Clone# PC61; Cat# 102026, Biolegend), PE-CY7-FoxP3 (Clone# FJK-16s; Cat# 25-5773-82, eBioscience) and PerCP-Cy5.5-ROR-γt (Clone# Q31-378; Cat# 562683, BD Pharmingen).
Validation	All antibodies were validated by manufacturer

Animals and other research organisms

Policy information about studies involving animals; ARRIVE guidelines recommended for reporting animal research, and Sex and Gender in Research

Laboratory animals	Wild-type C57BL/6J mice, B6.Cg-Foxp3tm2Tch/J mice.
Wild animals	This study did not involve wild animals
Reporting on sex	Mice in both genders were used. n=3 male, n=3 female
Field-collected samples	This study did not involve the use of the field-collected samples.
Ethics oversight	The animal use and care were in accordance with the institutional guidelines of NU, and all experiments were approved by the Institutional Animal Care and Use Committee of NU. IACUC protocol number: IS00026385

Note that full information on the approval of the study protocol must also be provided in the manuscript.

Plants

Seed stocks: n/a

Novel plant genotypes: n/a

Authentication: n/a

Flow Cytometry

Plots

Confirm that:

☒ The axis labels state the marker and fluorochrome used (e.g. CD4-FITC).

☒ The axis scales are clearly visible. Include numbers along axes only for bottom left plot of group (a 'group' is an analysis of identical markers).

☒ All plots are contour plots with outliers or pseudocolor plots.

☒ A numerical value for number of cells or percentage (with statistics) is provided.

Methodology

Sample preparation: 1. Bone marrow-derived mast cells were generated from the bone marrow of C57BL/6 wild-type (WT) mice[1]. Femurs were dissected, and the bone marrow was harvested by flushing the cavity using RPMI-1640 medium. Bone marrow cells were cultured with 15 ng/mL recombinant murine IL-3 in complete RPMI-1640 medium, supplemented with 10% fetal bovine serum (FBS), 1% penicillin-streptomycin, 2 mM L-glutamine, and 50 µM β-mercaptoethanol. Cells were cultured in a humidified incubator at 37°C with 5% CO_2, with fresh media and cytokines added every 5 days. Bone marrow-derived mast cells were harvested after 6-week culture.
2. Tregs were isolated from the spleens of C57BL/6 Foxp3-GFP reporter mice. Total CD4+ T cells were purified using BD anti-mouse CD4 Magnetic Particles. Subsequently, live Tregs were sorted by gating on propidium iodide-negative.
3. Cells were collected, washed twice with PBS, and centrifuged at 1,500 rpm for 5 min. Cells were then stained with Fixable Viability Dye 450 (Thermofisher Scientific, 65-0863-18) at 1:1000 in PBS for 10 min at 4 °C. After washing with FACS buffer (3 % FBS in PBS), cells were stained with surface markers, including CD4 (GK1.5) and CD25 (PC61) for 20 min at 4°C. For the detection of intracellular proteins, the FoxP3/Transcription Factor Staining Buffer Set (Thermofisher Scientific#00-5223-56) was used. Cells were stained according to the manufacturer's recommendation after the cell surface stain. Intracellular antibodies included FoxP3 (FJK-16s) and ROR-γt (Q31-378).

Instrument: BD BD FACSymphony™ A5 SE; BD LSRFortessa X-20; Sony SH800 cell

Software: BD FACS Diva software, Flowjo

Cell population abundance: reported in figures and methods

Gating strategy: reported and visualized in figures

☒ Tick this box to confirm that a figure exemplifying the gating strategy is provided in the Supplementary Information.
