# Supplementary material for: Spatial transcriptomics atlas of inflammatory bowel disease to guide implementation in research consortiums and clinical trials
Source: Nat Commun. 2026 Apr 28;17:5808. doi: 10.1038/s41467-026-72482-w (PMC13328602; doi:10.1038/s41467-026-72482-w)
Supplement: Supplementary file 14 — Transparent Peer Review file [file 41467_2026_72482_MOESM14_ESM.pdf]

nature portfolio

Peer Review File

Spatial transcriptomics atlas of inflammatory bowel disease guides implementation in research consortiums and clinical trials

Corresponding Author: Professor Parambir Dulai

This file contains all reviewer reports in order by version, followed by all author rebuttals in order by version.

Version 0:

Reviewer comments:

Reviewer #1

(Remarks to the Author)
The paper entitled "Spatial transcriptomics atlas of inflammatory bowel disease to guide implementation in research consortiums and clinical trials" by Yiming Li et al. presents a comprehensive comparison of two leading single-cell-level spatial transcriptomics (ST) platforms—CosMx and Xenium—using 66 intestinal biopsies from both healthy individuals and IBD patients across various disease states and locations. By generating a spatial atlas of 2.5 million intestinal cells, the authors demonstrate that CosMx consistently provides higher data quality and better biological resolution, including the unique identification of Treg-associated biology in ulcerative colitis and Crohn's disease, which was further validated in vitro. The findings underscore CosMx's robustness to sample handling variables, making it suitable for multi-center studies, and establish a valuable benchmark for future ST applications in IBD research.
Overall, this is a well-executed and timely study that addresses a critical need for platform validation in translational IBD research. These are technologies that are rapidly evolving and expensive. Their application to intestinal samples is very necessary because there is a relative paucity of such data in biopsy samples and where there are data, the number of samples is very small. We commend the authors for their rigorous approach and clear relevance to ongoing consortia efforts. We particularly appreciate investigating some of the biology of the mast cell-T Reg potential interaction. Nevertheless, we have a few questions and suggestions for clarification that could further strengthen the manuscript. In general, our comments involve re-analysis of the existing data—not doing additional samples and a more balanced perspective on the benefits of each approach given the inherent differences in gene panels and opportunities for varying the threshold for inclusion of samples or genes of interest.
Comments:
1. The study applies different QC thresholds across platforms (≥20 unique molecular identifiers (UMIs) for CosMx vs. ≥5 for Xenium; Methods, lines 547–557). While platform differences may justify this, the rationale is described only as based on "data visualization" (Supp. Fig. 5). Would the authors consider showing whether CosMx's reported superiority is robust to a uniform QC threshold across platforms? This could clarify how much of the observed advantage reflects filtering choices versus true performance.
2. The authors performed differential expression analyses using overlapping genes between CosMx and Xenium panels (e.g., 119 genes for the multi-tissue panels, Fig. 2b; 2,552 genes for the 6K/5K comparison, Fig. 3c, line 1020). However, PGK1—though present on both CosMx 6K and Xenium 5K panels—was detected only in CosMx. This discrepancy is noted (lines 221–224), but the Discussion (lines 304–310) primarily attributes platform performance differences to global technical metrics rather than addressing gene-specific detection failures. Could the authors clarify whether the absence of PGK1 detection in Xenium reflects platform sensitivity, expression thresholding, or other technical limitations?
3. The manuscript reports that CosMx uniquely identifies Treg–mast cell interactions in ileal Crohn's disease (Results, lines 162–200; Fig. 2c–e). FOXP3 is included in the 119 overlapping genes (Fig. 2b), but its upregulation is only detected in CosMx. Similarly, IL-6 signaling is discussed as a key axis (lines 185–200), but it is unclear whether IL6 or IL6R were present or detectable in the Xenium panels. This distinction is not clarified in the Discussion (lines 304–310 or 366–368). Additional detail on whether these genes were present but undetected—or entirely absent—would strengthen claims of platform-specific biological discovery.
4. The authors note that CosMx leverages PanCK/CD45 protein markers for improved cell type annotation (Methods, lines 572–589), and they mention in Supplementary Figure 1 that this approach yielded more consistent annotations than RNA-only methods. However, they do not show whether biological signals—such as Treg signatures—remain robust in CosMx

using RNA-only annotations. Would the authors consider providing a control analysis using RNA-only annotation to clarify the extent to which protein markers influence biological interpretations?
5. The authors appropriately address segmentation differences by normalizing transcript data per cell area (Methods, lines 507–509) and apply this in specific comparisons (e.g., Fig. 1c, Fig. 1g, Supp. Table 2). However, it is not always clear whether this normalization was applied consistently across all comparisons, especially in downstream biological findings (e.g., Fig. 2, Fig. 3). A supplemental table presenting both per-cell and per-area results side-by-side could help readers interpret the impact of segmentation differences.
6. Some other concerns stem from the interpretation of Supplementary Figure 1a. There are substantial differences in the cell-type annotation results between platforms. A discussion or interpretation is lacking. Well-matched scRNA-Seq references were used to perform label transfer. Assuming these references are a good approximation to overall tissue composition (albeit underrepresentation of specific cell types in dissociated scRNA-Seq is well established):
• There is a huge difference on the reported proportion of absorptive epithelial cells, with Xenium results much more similar to estimates from the reference data.
• Annotation of COSMx samples report very high proportion of stromal (particularly endothelial) cells as compared to Xenium and dissociated references.
• Overall, at least for well-represented cell types, the observed trend is that Xenium samples correlate better with proportions from scRNA-seq references (with some exceptions).
These observations are hard to interpret without a quantitative assessment of how the various gene panels balance the representation of both major cellular compartments (epithelial/mesenchymal/immune) and more fine-grained cell types. However, it is reasonable to expect that these imbalances affect many of the results and interpretations throughout the paper. This needs to be addressed before any differential analysis or assessment of performance is conducted.
To that end, we would suggest:
• Providing quantitative results on the label transfer analysis (e.g, prediction scores if the Seurat framework is being employed) and an assessment of how the different panels recapitulate unambiguous cell populations. This would set the stage on what is the biology captured by each of the platforms and panels employed. For small panels, a UMAP visualization of the data can also provide some insight into how each panel is able to distinguish cell compartments and types.
• On a related note: Can the authors provide an explanation as to why the cell-type specificity and granularity of unsupervised clustering results (Supplementary figures 6 and 8) seems to be much higher for Xenium, as compared to COSMx?
7. Figure 1b: it should be noted that global markers (EPCAM and PTPRC), are to various degrees downregulated in inflamed samples in both platforms.
• It needs to be clarified if the specific analytical choices made by the authors limit the scope of their differential expression results. Differential expression was performed at the level of whole tissue/sample, across disease groups, not at the cell-type level. In this respect, important analytical details are missing:
o Was the data normalized with SCTransform using only the intersection of genes in both panels (~100), or for all genes in the panel ~500 for Xenium, 1,000 for COSMx)?. Variance stabilization might be affected by overall sample signal for small numbers of genes, due to limited information to estimate the mean/variance distribution.
o Given that the tests are run in whole tissue/sample, the overall expression level and cell-type specificity of the non-intersecting genes (specific to each panel) is likely to impact that of the intersecting genes. So, all these results might be dependent on the specific biases of each panel, not only to platform-specific technical factors as the authors suggest. The effect sizes across different panels can hardly be compared to each other.
o For instance, although the two platforms were used on the same samples, the baseline differences in the panels (non-intersecting genes, a majority) results in an artificial compositional shift between the platforms. Downregulation of EPCAM in inflamed samples is expected due to lower percentages of epithelial cells in inflamed tissues, the downregulation of PTPRC is striking. How significant is the upregulation of genes like VWF which may be linked to a higher annotation rate of endothelial cells? Issues like this result in uncertain. Regardless, what seems to emerge here is that the differential expression analysis performed by the authors, and its association with cell type abundances, are unrelated with true transcriptional regulation. Unless a clear explanation or re-analysis (e.g. by cell type) is provided, the interpretation of differential expression results should avoid using terms like "up-regulation" and/or restrict the discussion to compositional changes or provide interpretation contingent on the gene composition of each panel.
o An example of these issues is actually apparent in Figure 1b. Even though the figures were truncated in the x axis, Supplementary Table 5 clearly shows that the most downregulated genes in the COSMx inflamed samples are all B-cell and epithelial constitutive genes (IGKC, IGHG1, IGHG2, IGHA1, MALAT1, S100A6, JCHAIN, B2M, PIGR, CD74, HSP90AA1, KRT19, KRT20, NEAT1, EPCAM), likely associated with differential composition/annotation results. For Xenium, the most downregulated genes in inflamed CD are also sets of epithelial (AQP8) and B-cell markers different to those in COSMx, due to different panel representation. Whether this affects differential expression across all other compartments is unclear but likely, similar to classical issues on the interpretation of Bulk RNA-Seq. Therefore, affirmations like "CosMx identified significant up-regulation of T cell markers in inflamed ileal CD and Xenium identified significant downregulation of T cell markers in inflamed ileal CD" are inaccurate and likely due to the differential representation of cell-specific genes across the different panels, not to the inability of any given platform to detect bona-fide transcriptional regulation.
8) The same concern applies to the discussion on FOXP3+ and Tregs: differential expression for FOXP3 is not even reported in the supp. tables for the Xenium tests. This is likely due to FOXP3 not passing arbitrary thresholds (genes detected in 1% of cells with effect size >0.1). For bigger panels, the thresholds employed are at least 10% detection, which is a rather stringent threshold and limits the differential expression tests to predominantly constitutively expressed genes. Given that the number of genes in the panels is small and differential expression is done at the sample level, it is unclear why the authors needed to impose those thresholds prior to running the tests.
9) Regarding differential expression in UC, the authors link the results on genes like CSF3 and PGK1 to the outperformance of COSMx. Why the authors focused on these genes is unclear. It is likely that PGK1 did not pass the detection threshold in

XENIUM. It could also be argued for example that: according to test results in XENIUM, the most upregulated gene in UC is DUOX2 (not shown if Figure 3b, which is truncated), a classical marker of inflammation in UC. This gene is not captured by COSMx, likely because it is not even present in the panels for this platform.
10) The most distinct advantage of spatial analysis is to provide insights into co-localization and niche composition associated with disease phenotypes. By itself, the results provided are relevant and informative, but due to the ambiguities highlighted above, all the cell-cell communication analyses are strongly influenced by the results of cell-type annotations, which require a more profound analysis of the effect that panel composition has on these analyses. In this respect, it can hardly be addressed which platform may provide a more informative account of changes in cell-cell interactions.
11) Finally, the authors claim that previous comparative analyses have indicated an outperformance of Xenium as compared to COSMx. The authors emphasize that these studies are in pre-print format, which is not surprising being an emerging field. It should not prevent the authors to provide a more neutral assessment. A recent preprint on FFPE mucosal biopsies, which is also referenced by the authors (Meninillo et al), provides compelling support for the outperformance of the Xenium platform in terms of cell-type annotations (including granulocytes) and niches associated with differential therapy response. This preprint analyzes and discusses the effect of segmentation, downplay its impact to a limited number of cells, and provides distinct biological insights.
Regardless, there are numerous comparative studies both published and in pre-print format that have evaluated the pros and cons of each platform, and multiple examples exist in support of each. Here, the authors aspire to provide recommendations for the adoption of a specific spatial technology in a very specific setting, that is screening FFPE samples in multi-center studies in Inflammatory Bowel Disease. And to this end:
• Assessment of the technical performance of different platforms, particularly related with the effect of cell segmentation on downstream analysis, is highly dependent on the context, implementation and computational methods that are in continuous development. A definite recommendation in this regard is not possible.
• Even using high plex panels, most users of spatial technologies recognize the need for panel customization. In the specific case of IBD, the small plex gene panels provided by COSMX and Xenium (even the colon-specific panels) miss a significant number of disease-relevant and cell-type specific genes. In our experience, even the high plex ~5K panels do not include a good number of relevant genes. Therefore, in terms of biological performance, it is hard to believe that large-scale, consortium-like studies on a specific disease and tissue will rely on manufacturer, generic gene panels, like the ones employed in this work. Given that different disease phenotypes will be evaluated using these approaches (e.g. response to therapy, fibrotic complications, and so on), there is a need to design custom targeted panels. Therefore, a more informative recommendation in the context of IBD will require a detailed evaluation using appropriate panels that better represent the molecular pathways involved in disease pathogenesis. The authors could inform the field by highlighting the genes or pathways particularly lacking in each of the “off the shelf” xenium vs COSMX approaches
Minor comments
The last sentence in the introduction says: “We anticipate that our work will set the standard for IBD consortiums as well as other gastrointestinal conditions in this evolving space.” This may be too bold a statement given how quickly this field is moving including improvements in analysis pipelines

Figure 1c is unreadable and hard to interpret.
Double check some of the references that have the wrong number (e.g. the neutrophil reference).

(Remarks on code availability)

Reviewer #2

(Remarks to the Author)
Spatial transcriptomics provide support in uncovering novel pathways and cell-cell interactions, however, the available data are limited. Here, 66 intestinal biopsies (FFPE) from non-diseased controls and IBD patients of varying disease locations were evaluated using ST platforms (CosMx; Xenium). A spatial atlas of 2.5 million intestinal cells is provided. Quality and quantitative differences were observed between the two platforms, with more advantages for the CosMx platform. The authors state that the study establishes a technical and biological benchmark for ST in IBD.

Overall, this is a technical paper analyzing the performance of both systems and other factors which is important for any future studies. The majority of the manuscripts is about the technical details (probably better judged by a person into those techniques).

In a second step, they analyzed ileal samples with contradicting results. For me, it is not clear which one is right. One possibility would be to add immunohistochemistry to prove one or the other. Given this revealed contradiction, how do we validate these results? Here, a path (based on the data provided) should be indicated (which will be important for any future analysis. The approach of going into the mouse system and evaluating splenocytes is not convincing when discussing ileal inflammation. Furthermore, the reasoning provided is questionable since anti-IL-6 in clinical trials did not result in an improvement but rather in more complications (Danese S et al. Gut 2019; 68(1):40-48).

The number of cells is derived from different sites and the patients represent a broad spectrum, leading to the question of how many cells from one region to make a statement, how many patients from one subtype to be sure about a finding? What is the necessary quality control? This appears important since only then will these datasets be of use to the community. This has not been addressed adequately

Thus, while significant for the technical advance of the field, the points above should be addressed, only then is this technical paper of value for future analysis.

(Remarks on code availability)
this is outside my area of expertise

Reviewer #3

(Remarks to the Author)
Stated goal of the manuscript is laudable – to compare leading ST platforms in a systematic way. The methodology also seems to be a strength – sequentially cut samples and doing a direct comparison of sequentially cut samples using Xenium vs. CosMx. Weaknesses include: uniformly poorer performance of one platform relative to the other, irrespective of factors that should affect data quality of all ST platforms (e.g. block quality, panel size, fixation time, sectioning timing); poorer data metrics for Xenium (e.g. transcripts per cell) reported by this manuscript compared to previously published studies; and functional experiments that neither validate nor refute observations made in the manuscript.

Specific points:

1. All data metrics observed in this study were poorer for Xenium compared to CosMx and this poor performance was independent of factors that should affect data quality of all ST platforms, e.g. block quality, panel size, fixation time, sectioning timing. This discrepancy raises the possibility that there may have been systematic issues in data generation and/or analyses in this study.
2. The authors report that 32.78% of Xenium cells had low transcript counts (< 5 transcripts per cell) (line 85-87). This seems to be much lower than the transcript counts per cell reported in the published and pre-print literature (which includes ~41 published papers that have used Xenium and ~26 pre-prints that have reported Xenium, from a quick and non-exhaustive Pubmed search). As two examples, (1) Figure 2b from PMID 40082609 (Marco Salas et al, Nat Methods 2025) shows an average of ~500 transcripts per cell and (2) Figure 3e from PMID 38114474 (Janesick et al, Nat Communications 2023) shows a median of 166 transcripts per cell. Though this metric will vary depending on the study and tissue analyzed, this discrepancy again raises the possibility that there may have been systematic problems in data generation and/or analyses in this study.
3. The authors appropriately cite four pre-prints that aimed to systematically compare Xenium and CosMx, all of which came to a different conclusion than the current study. The authors state that the reason for their discrepant conclusions was a "lack of consideration for cell segmentation variability across platforms, potential impacts of increasing plex size" in the other studies. However, the authors tested these variables here and found that accounting for these variables did not result in improved performance of Xenium. Moreover, the authors state that "Xenium's expanding boundary detection model results in significantly larger cells, which increases the surface area available for transcript assignment" and therefore should result in higher per cell transcript counts for Xenium. However, even when accounting for this variable, they did not see improved performance for Xenium on both a transcript per cell area and transcript per cell basis. Hence, the variables that the authors discuss do not seem to explain the discrepant conclusions of their study and the other four pre-prints (as well as previously published papers).
4. The level of IL-17A expression shown in Figure 2E-G is extremely low (0.83 – 3.16%) and it is unclear whether this represents signal or noise. It is also unclear whether a change from 0.83% to 3.16% of Treg cells expressing IL17A is a biologically meaningful difference. It might be more informative to test Treg function, such as suppressive ability.
5. The data shown in Figure 3g-I do not seem to support the authors' conclusion. The authors first show that PGK1 is differentially expressed in UC vs healthy rectum. The authors then show that PGK1 is upregulated in Th1, Th2, Th17, and iTreg compared to Th0 (Fig. 3f) (statistically significant between Th0 vs Treg because the Treg group has somewhat less variability than Th2 and particularly Th17). In vitro treatment with a PGK1 inhibitor results in increased MFI of both RORgt in Th17 cells and increased MFI of Foxp3 in iTreg cells, which would imply that increased PGK1 expression in UC leads to both decreased Th17 function and Treg function. It is unclear how the combination of decreased Th7 and decreased Treg function would impact inflammation.

(Remarks on code availability)

Reviewer #4

(Remarks to the Author)
The manuscript by Yiming Li et al with the title: "Spatial transcriptomics atlas of inflammatory bowel disease to guide implementation in research consortiums and clinical trials" is a very well written paper, dealing with the comparison of two major spatial transcriptomics platforms for patient samples with inflammatory bowel disease (IBD). They come to the conclusion that the CosMx platform outperforms the Xenium platform, but more importantly the CosMX platform enables biological discoveries that were not identified by Xenium.
As the overall manuscript is especially important for the IBD community, the spatial transcriptomics field may also benefit from the results. Some very important aspect of the manuscript includes:
-Block quality and fixation assessment
-Reanalysing Xenium data with better segmentation approaches
-Consecutive analysis of tissue sections with 2 methods
-Confirmation of cell interactions by in vitro experiments and mouse models
Nevertheless, there are critical parameters and results which need to be addressed.

- Suppl Figure 1
As scRNA-seq is used as a “reference” for cell type abundance, I wonder which platform represents the cell type abundance more closely? It appears there are often stark differences between what scRNA-seq found, and what Xenium or CosMx detected. Which is reflecting the true biology better? For instance, absorptive epithelium ranges from ~5-25%, depending on the technology. What does the corresponding tissue section show? This should be evaluated by a pathologist, and can be compared with IHC, or the CosMx protein-gated approach. And similar with IHC of Xenium section (consecutive or same section). Otherwise, it is not possible to decide which method is more accurate in depicting biology.
-Results:
Line 152+: A major impact on the comparison of the two technologies has the filter selection. The simple cut of of eg. 50 transcripts per cell to remove low quality cells seems arbitrary. As I understand, this threshold was selected for the Xenium 5k and CosMx 6k, although the technology for RNA detection is different. To set such a singular threshold, you would need to show that for instances cells with <50 transcripts using Xenium cannot reliably profile cells, such as epithelium, vessel endothelium or immune cells. Generally, a ground truth, supported by a pathologist and IHC would be essential. Having lower number of signals per cell does not mean a cell is of low quality or wrongly phenotyped.
The result of this threshold seems dramatic: in one sample 91% of cells were excluded in Xenium vs 2% only in CosMx (suppl table 3). This could be from too tissue quality, but this could also be from too stringent filtering.
A preprint comparing CosMx and Xenium and come to other conclusions https://doi.org/10.1101/2024.12.23.630033). As it is a non peer reviewed preprint, they need still further evaluation to judge the quality of the preprint. Nevertheless, similar to the here mentioned preprint, it would be important to include antibody based ground truth. Then you could evaluate at which threshold setting the technologies fail to detect major cell types .
Further stressing the filter setting, all downstream comparisons such as the detection of CSF3+ positive cells is affected (Fig 3). As you state in your manuscript, that Xenium 5k panel and multi-tissue had lower sensitivity than CosMx 6k panel in detecting this important expression marker, I wonder if this is a filter artefact, or biological truth.
Similar holds true for the detection of FoxP3+ Tregs co-localization with mast cells and T cell marker up regulation detected by CosMx, but downregulation in Xenium. Does it hold true if the filter settings are changed?

Line 100: Instead of re-segmentation, investigating nuclear transcripts rather than all transcripts, would circumvent segmentation issues, as suggested in https://doi.org/10.1038/s41592-025-02617-2 . This could be interesting to assess.
Line 279: The change to an alternative reference atlas which contains neutrophils makes sense. Discussing the aspect of reference atlases, irrespective from CosMx or Xenium would be important to include.
Line 366+: For spatial biology discovery, potentially whole transcriptome platforms would be more beneficial due their untargeted nature.
Discussion: Time and costs of the technologies would be interesting to add to the discussion.

Minor comments:
Line82: The list in Supplementary Table 1 is incomplete
Supplemental table 5 + 6: The p values are on the next pages, and it is not possible to identify the corresponding gene

(Remarks on code availability)
The code is available including a readme file, but I am not qualified in evaluating the code.

Reviewer #5

(Remarks to the Author)
The authors systematically compared two spatial transcriptomics platforms using intestinal biopsies from IBD patients and healthy controls. They provided benchmarking of the data to guide the integration of spatial transcriptomics in multicenter studies. They also validated platform-specific findings through targeted in vitro experiments. Overall, this is a valuable comparative study with direct relevance for the IBD research community and broader spatial biology field.

• The authors used Baysor for re-segmentation, but did not sufficiently clarify how differences in segmentation directly affect biological outcomes or interpretations. How did you ensure that the performance difference between CosMx and Xenium was not significantly influenced by cell segmentation methods, and how this influenced the downstream biological interpretation

• Could you clarify why platform-specific QC thresholds (20 UMIs for CosMx, 5 UMIs for Xenium) were chosen, and whether a unified threshold would influence the comparative conclusions? Did the authors test a standardized method for a more unbiased comparison?

• Can you explain why key genes (e.g., FOXP3, PGK1) show differential expression patterns specifically between platforms? Could this reflect differences in probe chemistry or hybridization efficiency? There is a significant drop in Xenium platform performance with increasing panel size and critical differences observed between platforms regarding negative probe counts and RNA quality sensitivity, the authors need to test different normalization and QC methods across platforms to ensure the accuracy of the downstream biological interpretation

• Could the authors provide more details on the number of independent biological replicates and the reproducibility of their in vitro experiments validating key genes (FOXP3, PGK1)? Did variability exist across replicates?

• In spatial cell-cell interaction analyses, did the authors assess how robust and reproducible these interactions were across multiple samples, particularly in biopsies from heterogeneous disease states?

• Some figure legends (particularly Figures 2 and 3) are overly dense or lack clarity on replicate numbers and statistical tests.

• Currently, limitations are discussed in broad terms without explicitly addressing technical biases or specific limitations of spatial transcriptomics technologies.

• Abbreviations and terminology (e.g., "multi-tissue" vs. "MT", "segmentation" vs. "seg.") sometimes vary across figures and text.

• The authors could try to clearly define axes, labels, and legends with concise text, increasing font size and readability of the figures

(Remarks on code availability)

Version 1:

Reviewer comments:

Reviewer #1

(Remarks to the Author)
In response to this and other reviewers' concerns, the authors now provide:

* Additional assessment and discussion on how QC metrics can affect the evaluation of Xenium performance in the samples analyzed
* A small multi-omic spatial dataset on the COSMx/CellScape platforms.

Although the authors are leaders in a field that is not yet crowded, these technologies are rapidly evolving. It seems unlikely that one platform is significantly better than the others - instead, the best platform will depend on the question being asked.

Unfortunately, I am not satisfied with the author's responses to most of my major concerns.

My major comments are as follows:

1. The authors continue to leverage observations on a few genes to overstate the performance of the COSMx platform compared to Xenium in clinical applications. The authors refer to these hand-picked examples rather than providing a direct and convincing response to most of my original questions.

2. In terms of technical performance, I am still surprised that the Xenium platform is reported to perform so poorly in this manuscript. Most of the authors' assessments are based on diversity and detection, yet it is unclear how these metrics are normalized. I provide more specific examples below. Here, although the authors cite at least 7 studies where Xenium shows superior performance, they argue that those evaluations are not relevant due to limited sample size and other factors like technical differences and biological insights. In my opinion, this is a biased and arbitrary assessment of the literature. The results herein contradict those from leading experts in spatial transcriptomics (e.g., https://www.biorxiv.org/content/10.1101/2023.12.13.571385v1.full.pdf; please note that I am not related in any way to this reference), who have reported that the COSMx platform has a concerningly low dynamic range, low signal/noise ratio and inflates the signal of low abundance genes. This last factor alone could explain a significant proportion of the differences observed by the authors between the two platforms (e.g., in diversity).

3. Finally, the authors repeatedly refer to the relevance of the results presented here in the context of setting the standard for applying ST technologies in the NIDDK and in other IBD consortia. This manuscript constitutes a significant technical effort. However, the choice of metrics to evaluate performance is narrow and contradicts many previous studies, and it is difficult to draw conclusions about depth, generalizability, and biological relevance from a few hand-picked examples. In my opinion, the results presented here don't constitute an unbiased assessment, nor do they prove that one platform displays superior performance that would justify its promotion as the best choice for large-scale efforts.

Specific comments follow:

Authors' response R1A4-5: The authors’ response to all my original concerns is limited to highlighting their Foxp3/Tregs findings, ignoring other important cell types or compartments. However, some of the concerning features of their COSMx cell composition results (overestimation of endothelial cells, underestimation of absorptive epithelium) cannot be justified by merely referring to unexplained, potential biases of dissociated scRNA-seq studies.

The authors do not address my concerns that the supplementary heatmaps highlight the superior specificity of cell-type calls in Xenium compared to COSMx.

Authors' response R1A7: I strongly disagree. As a user of ST platforms, I don't think that cohort and clinical studies that apply ST technology will be conducted using whole-tissue differential expression. The major advantage of ST data is to provide spatial context for cell-cell interactions and to validate relevant cell states, both of which require high specificity at the single-cell level. From this perspective, the authors' results instead demonstrate that the Xenium platform exhibits better overall performance across all cell compartments.

A careful examination of the Supplementary Data provided by the authors (there is no visualization or summarization of these results) reveals that the reliability of cell annotations is superior in Xenium compared to COSMx. This improvement in performance is quite substantial and is consistent across the board and for all combinations of QC metrics. Notably, even Treg annotation is more reliable in Xenium compared to COSMx. The annotation of Tregs is a critical element of any single-cell genomics experiment, and on in its own, would drive many users to favor Xenium technology.

Authors' response R1A8: Again, the authors' response to all my original concerns is limited to highlighting their Foxp3/Tregs findings. My concerns about how whole-tissue differential expression was assessed and interpreted remain.

The authors should make an additional effort to provide a comprehensive interpretation of their results, instead of focusing on just a couple of hand-picked genes. Using their logic, one could argue that Xenium is better because DUOX2 and HK2 are highly relevant genes in IBD that are only detected (and found differential) by this platform. There are numerous examples that I could refer to which raise questions, including the finding (via COSMx) of highly significant upregulation of several X chromosome genes (SPANXB1, SSX4) in inflamed samples, though the relevance and/or relationship of these genes in intestinal samples remains unclear.

I don't understand the argument that the percent of cells expressing CSF3 is informative. Which cells express CSF3 in their samples? The authors seem to value this metric given the importance of granulocytes for NIDDK IBD consortiums (although their reasoning is unclear). However, we already know that CSF3 is expressed by inflammatory fibroblasts and other cell types in UC tissues (https://www.nature.com/articles/s41467-023-40156-6, https://pubmed.ncbi.nlm.nih.gov/36720220/). Without a more in-depth assessment, the interpretation of the value of this metric seems superficial.

If the percent of cells expressing a gene is a meaningful metric, the authors could highlight in the text that their supplementary bar plots show that the Xenium samples have a higher percent of cells expressing CD14, CD2, CD3E, CD79A, IRF8, MKI67, KLRB1, etc.

Authors' response R1A9: To address this concern, the authors briefly refer to the addition of new proteomic data, which is not satisfactory. The new data, as presented in the manuscript (Figure 5, snapshots of a few hand-picked tissue sections showing the genes they have previously selected), is not a rigorous validation of the RNA analysis. My comments regarding the superior performance of Xenium in cell annotation, and therefore in any niche analysis, again apply here.

Authors' response R1A10: Regarding the evaluation by Meninillo et al., it should be noted that the authors' results are in agreement: Xenium exhibits superior performance in cell annotation, which is a critical element of ST analysis.

Other minor concerns:
* There are multiple references throughout the paper about the ability of COSMx to identify pathways of interest, but no pathway analysis was performed (that I could find), and there was no mention of pathway analysis in the methodology. Other genes (e.g., OSM) are only found in supplementary tables and aren't significantly ranked in the overall results. This pattern indicates some arbitrariness in the authors' interpretation of their results, leading to qualitative, unsupported remarks. I recommend that the authors refrain from upgrading some circumstantial evidence to the level of significant biological findings.
* "Spatial cell-cell interaction analyses of inflamed ileal CD in our CosMx data observed multiple interactions between FOXP3+ Tregs and other immune cells, including monocytes, macrophages, and mast cells, which were not seen in inflamed rectum CD or inflamed UC samples." What do the authors mean by spatial cell-cell interaction analysis? Where and how is this quantified? The authors later refer to "visual inspection"—if this is the case, please refrain from using the word "analysis" unless a quantitative analysis is performed and the methods are properly reported.
* Spatial proteomics, Figure 5: How are high/low Treg levels defined? What are the error bars in Figure 5g? How significant is the change of ~15% to ~25% of putative Tregs subsets expressing TGFB3, in the authors' view? How was TGFB3 selected over other genes for this analysis?
* In the same section, the authors claim that "CosMx multi-omics (64-plex protein panel + 6K RNA panel) of new inflamed ileal CD samples confirmed spatial co-localization of Tregs with macrophages using spatial proteomic annotations, and cell-cell interactions for pathways observed from our comparative analysis using spatial transcriptomics expression. (Fig. 5a)". Where is the quantitative analysis that supports this claim? Where is the cell-cell interaction and pathway analysis shown or described? Is the co-localization confirmation based exclusively on the two snapshots of a handful of cells shown in Figure 5e/5f?
* Unless I am misinterpreting the power calculations, the calculations shown to support the clinical application of ST platforms are based on the power to address transcript diversity. This choice needs to be justified, as it is my opinion that any power assessment in the context of genomics studies should be focused on the power to detect biologically/clinically meaningful variables, not transcript/gene diversity. In transcriptomics, for instance, one must estimate the number of replicates necessary to detect differentially expressed genes at a given false discovery rate. Instead, the power estimates provided here focus on transcript diversity, which could be misleading for readers that are concerned about well-powered transcriptomics studies.

(Remarks on code availability)

Reviewer #2

(Remarks to the Author)
no further comments

(Remarks on code availability)

Reviewer #3

(Remarks to the Author)
My original Critique 4: “The level of IL-17A expression shown in Figure 2E-G is extremely low (0.83 – 3.16%) and it is unclear whether this represents signal or noise. It is also unclear whether a change from 0.83% to 3.16% of Treg cells expressing IL17A is a biologically meaningful difference. It might be more informative to test Treg function, such as suppressive ability.”

Reviewer response “R3A4: We thank the reviewer for this point. We would like to clarify that the y axis is proportion of IL-17+ CD4+ T cells, not IL-17 expression levels, across the 3 groups. This was done in triplicate, and also the experiment was then repeated again with triplicates which re-confirmed the significant differences between groups. This is highlighted better in the figure legend now.”

This critique was not adequately addressed. The point is that the proportion of CD4+ T cells expressing IL17A is quite low (0.83-3.16%) and it is unclear whether a chance from 0.83% to 3.15% of Treg cells expressing IL17A is a biologically meaningful difference. I am not questioning whether the difference is statistically significant. I am questioning whether the difference is biologically meaningful. This is why I suggested testing Treg function instead.

However, this point can be addressed by revising the sentences in lines 234-239 to the following: “Tregs co-cultured with mast cells exhibited a statistically significantly higher level of IL-17A (3.16%) compared to Tregs cultured alone (1.01%). In addition, treatment of anti-IL-6 significantly reduced IL-17A levels in Tregs during co-culture with mast cells (0.83% vs. 3.16%, Fig. 2e), indicating a possible critical role of the IL-6 produced by mast cells. Additional functional experiments are warranted to confirm the biological importance of this finding. Recent clinical trial evidence…”

I’m not convinced that this manuscript has provided any new biologically meaningful discoveries, but perhaps an attempt to compare leading ST platforms in a systematic way is sufficient for publication. Moreover, I strongly agree with Reviewer 1’s critique 11: “Assessment of the technical performance of different platforms, particularly related with the effect of cell segmentation on downstream analysis, is highly dependent on the context, implementation and computational methods that are in continuous development. A definite recommendation in this regard is not possible.” However, I think that the authors have made an effort not to make overly definitive statements given frequent changes in technological and computational advances in this rapidly evolving field. I also think that they have tried their best to address the concerns of reviewers.

(Remarks on code availability)

Reviewer #4

(Remarks to the Author)
Overall, the authors have thoroughly addressed the concerns regarding quality control thresholds and filtering steps. They added several supplementary tables (4 and 5), revised the main figures, and included new data. Importantly, when appropriate quality parameters, such as standard Q-score filtering are applied, the authors now observe comparable biological insights from both the CosMx and Xenium platforms. This results in similar findings between the two platforms, particularly with respect to the role of FOXP3. Some statements and headlines seem overstated, which are pointed out below together with additional comments.

Critique 1: (Suppl Figure 1) As scRNA-seq is used as a “reference” for cell type abundance, I wonder which platform represents the cell type abundance more closely? It appears there are often stark differences between what scRNA-seq found, and what Xenium or CosMx detected. Which is reflecting the true biology better? For instance, absorptive epithelium ranges from ~5-25%, depending on the technology. What does the corresponding tissue section show? This should be evaluated by a pathologist, and can be compared with IHC, or the CosMx protein-gated approach. And similar with IHC of Xenium section (consecutive or same section). Otherwise, it is not possible to decide which method is more accurate in depicting biology.

R4A1: We thank the reviewer for this point which highlights that we must begin to consider what "ground truth" represents when looking at spatial technology. The non-destructive nature and limited tissue input required for these spatial technologies as compared to traditional dissociative scRNA-seq will result in variation in cellular population prevalence across technologies. To this effect, we have provided spatial proteomic based confirmation of cell type interactions and biology in tissue on the same slides where spatial transcriptomics was done to better guide how the two align. We feel spatial proteomics offers the most accurate estimate of cellular proportions for comparison to spatial RNA based annotation.
Comment by Reviewer 4:
I fully agree that the ground truth need to be further discussed in spatial technologies, but not necessarily in this manuscript. I agree that spatial proteomics can offer the most accurate estimate of cellular proportions compared to RNA based annotation. The authors added figure 5, which now compares one of the platform (GeoMx) with antibody based staining's.

Critique 2: (Results) Line 152+: A major impact on the comparison of the two technologies has the filter selection. The simple cut of of eg. 50 transcripts per cell to remove low quality cells seems arbitrary. As I understand, this threshold was selected for the Xenium 5k and CosMx 6k, although the technology for RNA detection is different. To set such a singular threshold, you would need to show that for instances cells with <50 transcripts using Xenium cannot reliably profile cells, such as epithelium, vessel endothelium or immune cells. Generally, a ground truth, supported by a pathologist and IHC would be essential. Having lower number of signals per cell does not mean a cell is of low quality or wrongly phenotyped. The result of this threshold seems dramatic: in one sample 91% of cells were excluded in Xenium vs 2% only in CosMx (suppl table 3). This could be from too tissue quality, but this could also be from too stringent filtering.
R4A2: We thank the reviewer for this point, and we have provided detailed metrics for both platforms when applying no QC filter versus when applying QC filters and different segmentation approaches we have used. (Supplementary Tables 4-5) The choice of QC filter was chosen based on proportional relationship to panel size (50/5,000) and direct visualization of data distribution as noted in Supplementary Figure 5. As noted in this figure for the Xenium 5K versus CosMx 6K panels we looked at how varying thresholds of QC cut-offs would influence comparative performance of platforms. As noted by the reviewer, even at a threshold of 50, some Xenium samples exhibited large numbers of cells being filtered. In Supplementary Figure 5, we see that increasing QC thresholds preferentially benefit Xenium metrics but the per sample comparisons still do not reach superiority relative to CosMx which stable up to a QC filter count of 200 UMI. We do agree that further work is needed to optimally define QC thresholds using various approaches including impact on cellular annotation, however, this is beyond the scope of this manuscript. We have highlighted the need for this in the manuscript revised text.
Revised Text: (Page 20) Along these lines, further work is needed to define optimal approaches to data quality control and filtering with consideration for probe specificity, probe gene detection confidence, and impacts on cellular annotations and cellular interactions. A detailed and biologically driven methodology for data filtering is yet to be developed for uniform application in spatial imaging-based technologies.
Comment by Reviewer 4:
The authors have addressed the critique 2 and added now data in supplementary figure 5, as well as in supplementary tables 4 and 5. The revised text is appropriate.

Critique 3: A preprint comparing CosMx and Xenium and come to other conclusions https://doi.org/10.1101/2024.12.23.630033). As it is a non peer reviewed preprint, they need still further evaluation to judge the quality of the preprint. Nevertheless, similar to the here mentioned preprint, it would be important to include antibody based ground truth. Then you could evaluate at which threshold setting the technologies fail to detect major cell types.
R4A3: We thank the reviewer for this point and agree that antibody-based ground truth is needed. In this regard, we provide spatial multi-omics data including a platform that uses primary conjugated antibodies (Cellscape). This multiomics validation of cellular interactions and spatial quantification of cellular proportions helps further strengthen the observations made in our study.
Comment by Reviewer 4:
The authors included Cellscape proteomics data, which is reassuring that the RNA spatial data is comparative to protein staining. Nevertheless, Fig 5 a is a bit unclear. The box in the upper panel shows single cells by proteomics. The lower panel highlights the RNA expression, such as FOXP3 etc: Two questions: What is the green stain in the lower panel? It looks like several stained cells. Second question: In the upper panel, is a single dot a single transcript? Please clarify. Although the antibody staining is now included, the authors did not show at which threshold setting the GeoMx technology is still able to detect major cell types, or when it start to fail. For Xenium, such data is not included.

Critique 4: Further stressing the filter setting, all downstream comparisons such as the detection of CSF3+ positive cells is affected (Fig 3). As you state in your manuscript, that Xenium 5k panel and multi-tissue had lower sensitivity than CosMx 6k panel in detecting this important expression marker, I wonder if this is a filter artefact, or biological truth.
R4A4: We thank the reviewer for this point. To address it, we explored gene specific probe detection confidence for Xenium 5K and observed that all samples had high confidence in probe detection. Meaning, whichever probes were detected for CSF3 in the panels was felt to be high confidence and filtering of low confidence genes was not a source of differences. We have included this in-depth analysis in supplementary table 7.
Revised Text: (Pages 10-11) In an inflamed UC sample, the Xenium multi-tissue panel detected 6-fold more cells expressing CSF3 (colony-stimulating factor 3) than Xenium 5K. However, both Xenium multi-tissue and Xenium 5K had substantially lower CSF3 expression than CosMx 6K in the same spatial regions (Fig. 3b), despite having high confidence in probe detection across panels (Supplementary Table 7).
Comment by Reviewer 4:
CSF3 has also often lower Q-scores (<20) in supplementary table 7. From my understanding, the standard Xenium pipeline would now remove this transcript due too low quality. This would lead to a similar situation as with FOXP3. Then it would be necessary to evaluated what will happen if the Q-Score filtering is adapted as you described in the revised manuscript starting line 202.

In line 243 the statement “CosMx uniquely identifies Treg biology in ulcerative colitis” seems overstated. As the important UC pathology marker CSF3 is detectable with both platforms GeoMx and Xenium, although potentially with less sensitivity. Similar: The headline at line 182: “CosMx uniquely identifies Treg-Mast cell interaction in ileal Crohn’s disease” needs to be adjusted, as this is Q-score filtering issue in the Xenium platform, as discussed by the authors

Critique 5: Similar holds true for the detection of FoxP3+ Tregs co-localization with mast cells and T cell marker up regulation detected by CosMx, but downregulation in Xenium. Does it hold true if the filter settings are changed?
R4A5: As noted in our overall response and detailed response to Reviewer 1, the differential detection of FOXP3 for CosMx and Xenium was due to low gene specific probe detection confidence for Xenium primarily in inflamed tissue. Upon removing the Q-score filter recommended by 10x, the FOXP3 gene does emerge as a differentially expressed gene, but it becomes difficult to ignore this filter because it is recommended by the company for removing low confidence probe detection and would introduce tremendous noise. Notably, this lower gene specific probe detection confidence for FOXP3 with Xenium was seen most predominately in inflamed IBD tissue highlighting the tissue specific variation for the platform and its influence on biological discovery/insights.
Comment by Reviewer 4:
This critique 5 is already well addressed to reviewer 1 and added in the revised manuscript.

Critique 6: (Results) Line 100: Instead of re-segmentation, investigating nuclear transcripts rather than all transcripts, would circumvent segmentation issues, as suggested in https://doi.org/10.1038/s41592-025-02617-2 . This could be interesting to assess.
R4A6: We thank the reviewer for this point and agree that assessment of nuclear versus non-nuclear transcripts would be of interest. We have added this detailed assessment in supplementary table 7 and note that CosMx remains superior to Xenium even when limited to per cell nuclear detection.
Comment by Reviewer 4:
Additional quantitative data was added by the authors. But does this for instance improve the detection of highly relevant genes like FOXP3, CSF3 and others?

Critique 7: (Results) Line 279: The change to an alternative reference atlas which contains neutrophils makes sense. Discussing the aspect of reference atlases, irrespective from CosMx or Xenium would be important to include.
R4A7: We thank the reviewer for highlighting the need to consider the reference atlas for annotation. We have added a new annotation section and also added a specific sentence highlighting the need to be mindful of the reference atlas.
Revised Text: (Page 14) This re-analysis with an alternative single-cell RNA sequencing reference atlas highlights the importance of defining optimal strategies for annotation to guide accurate biological discovery.
Comment by Reviewer 4:
Critique 7 is well addressed

Critique 8: Line 366+: For spatial biology discovery, potentially whole transcriptome platforms would be more beneficial due their untargeted nature.
R4A8: We thank the reviewer for this point and do agree whole transcriptome platforms may be more beneficial in their untargeted nature. We have not added a discussion to this effect in the manuscript because we have not yet fully evaluated CosMx WTX versus 6K, and would not want to prematurely suggest enhanced benefit without evaluating impacts to sensitivity with the panel size increase.
Comment by Reviewer 4:
Agree, manuscript is also already a complex topic.

Critique 9: Time and costs of the technologies would be interesting to add to the discussion.
R4A9: We thank the reviewer for pointing out this out and have added several sentences throughout on cost/time as a factor, particularly when considering customization.
Revised Text: (Page 19) Taken together, CosMx offers the best balance of feasibility for implementation, consistency in data quality, and detection efficiency, which makes it ideal for use in large multi-center collaborations and clinical trial programs for IBD given the cost and time intensive nature of these experiments.
Comment by Reviewer 4:
As a reader I do not see the costs or time invested in each technology to acquire and analyze the data. Simply stating that spatial technologies are time intensive by nature, is not very informative. It remains still interesting to know the time needed for each technology.

Critique 10: The list in Supplementary Table 1 is incomplete.
R4A10: We apologize for the confusion we may have created in our original submission on this table. This is only the overlapping genes between platforms, not the full panels for each platform.
Comment by Reviewer 4:
Critique 10 is well addressed

Critique 11: Supplemental table 5 + 6: The p values are on the next pages, and it is not possible to identify the corresponding gene.
R4A11: We thank the reviewer for pointing this out. The DGE analyses now have the p value and adjusted p value on the same sheet as the gene information for CosMx and Xenium separately.
Comment by Reviewer 4:
Critique 11 is well addressed

(Remarks on code availability)

Reviewer #5

(Remarks to the Author)
The authors have substantially addressed the major concerns raised in the initial review, and the revised manuscript represents a clear improvement in methodological rigor and interpretive balance. The added analyses examining results with and without QC filtering, together with run- and gene-level Q-score assessments, convincingly demonstrate that several previously noted inter-platform discrepancies reflect technical sensitivity rather than fundamental biological disagreement, including the clarified case of FOXP3. The inclusion of quantitative label-transfer confidence metrics and orthogonal spatial proteomic/multi-omic validation strengthens confidence in cell-type annotation, while the added power considerations and discussion of platform version effects appropriately temper cohort-level claims. One remaining minor suggestion is to further ensure that the Abstract and concluding statements consistently emphasize robustness trends and methodological insight rather than platform equivalence or broad generalizability. Overall, the substantive reviewer concerns have been adequately addressed, and the remaining issues are largely editorial.

(Remarks on code availability)

Version 2:

Reviewer comments:

Reviewer #1

(Remarks to the Author)
The authors have done a thorough job of responding to our critiques. The data and analytic strategies presented will inform the field (at least for the time being) in terms of using spatial transcriptomics to better understand IBD pathogenesis and the effect of interventions at a more granular level

(Remarks on code availability)

Reviewer #3

(Remarks to the Author)
The theme of the comments from all 5 reviewers is that this manuscript tends to overstate claims and exaggerate the broad generalizability of their findings. The 5 reviewers all suggested that the authors thoroughly revise their manuscript emphasize robustness trends and methodological insight rather than make broad conclusions about universal platform superiority vs. inferiority and the generalizability of their findings.

The authors have appropriately made revisions, but should review their manuscript carefully and continue to revise the manuscript, particularly the Discussion section, where they continue to overstate their conclusions.

(Remarks on code availability)

Reviewer #4

(Remarks to the Author)
Overall, the authors have responded well to all my comments raised. They have reduced the overstatement regarding the superiority of one platform over the other, by focusing on their IBD cohort and addressing filtering issues. The manuscript is now more balanced.

(Remarks on code availability)

Reviewer #5

(Remarks to the Author)
The revisions have addressed the major concerns raised in the initial review.

(Remarks on code availability)

Open Access This Peer Review File is licensed under a Creative Commons Attribution 4.0 International License, which permits use, sharing, adaptation, distribution and reproduction in any medium or format, as long as you give appropriate credit to the original author(s) and the source, provide a link to the Creative Commons license, and indicate if changes were made.
In cases where reviewers are anonymous, credit should be given to 'Anonymous Referee' and the source.
The images or other third party material in this Peer Review File are included in the article's Creative Commons license, unless indicated otherwise in a credit line to the material. If material is not included in the article's Creative Commons license and your intended use is not permitted by statutory regulation or exceeds the permitted use, you will need to obtain permission directly from the copyright holder.
To view a copy of this license, visit https://creativecommons.org/licenses/by/4.0/

<u>NCOMMS-25-36839 – Response to the Reviewers</u>

Introduction to Resubmission

We thank the reviewers for their constructive comments. Below we provide a broad summary of the substantive changes and additional analyses added to address concerns about quality control (QC), Xenium data quality, and cell type annotation, and where these results are presented in the revised manuscript and Supplementary Materials. All revisions in the manuscript are outlined in red text, and we have provided 2 sections for this response to reviewers: 1) a general overview of edits made to help orient all reviewers to broader revisions, and 2) a point-by-point response to reach reviewers individual critiques to help ensure we have addressed all points fully. We thank the reviewers for helping to substantially strengthen the manuscript quality and rigor.

Quality control thresholds and their impact on comparative performance

Reviewers highlighted the need to justify the QC thresholds used, and whether these QC thresholds may have influenced interpretation of comparative performance due to the variable degree of filtering across samples seen for Xenium. We agree that QC thresholds are not yet standardized for imaging-based spatial transcriptomics data and therefore must be chosen based on platform panel size/composition and data quality. The reviewers will note in the revised submission that we now include comparative results with no QC applied and across various segmentation approaches in Supplementary Tables 4 and 5.

With no QC applied, the comparative performance is more in favor of CosMx with higher median diversity in 15 out of 16 multi-tissue samples and higher median transcript counts in 12 out of 16 samples (Supplementary Table 4). Applying a minimal QC threshold (5 UMI) for Xenium filtered out low quality Xenium cells and resulted in substantial improvement in Xenium's relative performance, whereas QC for CosMx resulted in minimal filtering and therefore little improvement in its comparative performance. Therefore, applying the QC thresholds (Xenium – 5, CosMx – 20) used in our main comparative analyses shifted the comparison in favor of Xenium because a greater fraction of low-quality Xenium cells has been removed. In this regard, applying the current QC thresholds bias the comparative performance in favor of Xenium, but we still adopted the current approach to ensure inclusion of only higher quality Xenium cells in cohort analyses. Increasing the QC thresholds of Xenium to a more stringent level resulted in extreme cell loss and made comparative results difficult to interpret. Additionally, we found that QC filtering had minimal impact on comparative performance across different segmentation approaches used for Xenium (Supplementary Table 5). We have now added text to Methods and Results to explain the rationale behind QC filtering, sensitivity to threshold choice, and how thresholding affects analysis results interpretation.

Xenium data quality and probe detection confidence (Q-scores)

Reviewers brought up whether global and/or per gene probe detection efficiency may have influenced comparative performance and biological insight differences between platforms. We would like to thank the reviewers for highlighting this point as it allowed us to further explore the concept and the observations have strengthened our paper significantly.

In response to the reviewers' comments, we now report per-sample global Q-scores for Xenium runs in Supplementary Table 2. This metric quantifies probe detection confidence and is recommended by 10x Genomics as a measure of run quality. It was also used by the Xenium specialist employed by 10x Genomics, as part of the review for data generated from NUSeq Core at Northwestern University (an independent Core facility certified by 10x Genomics to provide high quality data), to ensure high quality runs prior to inclusion in the manuscript. As shown in Supplementary Table 2, all Xenium runs included in the manuscript exceeded the Q-score threshold recommended by 10x Genomics (Q-score > 20). We subsequently reviewed the per-gene Q-scores for all the samples separately (Supplementary Table 7) and found that most genes showed high quality Q-scores greater than 20 (for example, 347 out of 377 genes in the Xenium multi-tissue data used for comparative analysis).

When examining the per sample variability in per-gene Q-scores, we identified a meaningful exception for FOXP3, a gene known to be highly relevant to inflammatory bowel disease (IBD) and a key gene of interest based on our differential expression analysis results and Treg biology case study. Although FOXP3 had a high aggregated (median) Q-score across all the samples, its per-sample Q-scores were highly variable. Specifically, the inflamed samples showed reduced and less consistent detection confidence for this gene while other genes in the same inflamed tissue runs had high Q-scores that were more consistent. Xenium's data export pipeline may automatically filter out individual genes with low Q-scores (Q-score < 20), and this raised a question regarding whether this Q-score filtering step diminished FOXP3 signal when comparing inflamed with non-inflamed in differential gene expression analysis. Indeed, when we re-analyzed Xenium data without per-gene Q-score filtering, FOXP3 was found to be differentially expressed in the same comparison, which is consistent with our results on CosMx data.

Our per-sample and per-gene Q-score analyses therefore support three conclusions: (1) the Xenium runs were of high quality, (2) a vast majority of panel genes had consistently high detection confidence across samples, but (3) per-gene, per-sample Xenium performance variability in inflamed IBD samples resulted in low detection confidence for a highly biologically relevant gene (FOXP3) which affected cohort-level differential analysis results, whereas the consistency in probe detection efficiency in CosMx data allowed us to identify unique biology that was missed by Xenium. Since our main study aim is to guide cohort studies and clinical studies, we recognize that the consistency in probe detection efficiency across different genes and samples is critical for reliable downstream analyses like differential gene expression analysis in cohort studies. We have revised our manuscript to reflect this observation and update the main figures and supplementary materials accordingly.

Cell type annotation confidence and spatial proteomics integration

Reviewers highlighted the importance of cell type annotation, understanding annotation confidence across platforms, and orthogonal protein confirmation of spatial cell-cell interactions. We have therefore performed the following additional analyses which *includes new data generation*: (1) analysis of Seurat label transfer prediction scores to understand the relative confidence in annotation approaches across different platforms, and (2) integration and analysis of spatial multi-omics data, including spatial proteomics data across two platforms (CosMx and Cellscape) and same-slide spatial transcriptomics (CosMx), to confirm RNA-based biological insights regarding cellular interactions.

To quantify annotation confidence across platforms, we computed the Seurat label transfer prediction scores and reported these systematically in Supplementary Table 9. Xenium had higher overall confidence in cell type annotation for major cell types, but presented wider variability for fine-grained annotations. In particular, Tregs were noted to have lower cell type annotation confidence in both platforms. This and the fact that our protein-gating strategy for CosMx annotation improved cell type annotation confidence, further justified the need for protein-based annotation and spatial multi-omics integration, as noted by the reviewers.

Our multi-omics spatial data confirmed RNA-based biological insights from CosMx data regarding Treg spatial interactions and cell-cell interactions. In the manuscript, we now highlight the value for integrating spatial proteomics (CosMx) with spatial transcriptomics (CosMx) data when resolving cellular heterogeneity. Additional work is needed to develop new methodology and foundation models for RNA-based and multi-omics/multi-modal-based cell type annotation of spatial imaging-based data, given the data distribution differences compared to traditional scRNA-seq data. In response to the reviewers' comments, we have now added discussion about this in the manuscript to highlight the benefits and need for such work.

Reviewer #1

Overall, this is a well-executed and timely study that addresses a critical need for platform validation in translational IBD research. These are technologies that are rapidly evolving and expensive. Their application to intestinal samples is very necessary because there is a relative paucity of such data in biopsy samples and where there are data, the number of samples is very small. We commend the authors for their rigorous approach and clear relevance to ongoing consortia efforts. We particularly appreciate investigating some of the biology of the mast cell-T Reg potential interaction. Nevertheless, we have a few questions and suggestions for clarification that could further strengthen the manuscript. In general, our comments involve re-analysis of the existing data—not doing additional samples and a more balanced perspective on the benefits of each approach given the inherent differences in gene panels and opportunities for varying the threshold for inclusion of samples or genes of interest.

R1A0: We thank the reviewer for noting the strengths and value of our study, and sincerely appreciate the thoughtful comments provided which we have worked to address with both *additional analyses* ***and*** *new data generation*. We also agree with the reviewer that a more balanced tone to the manuscript is needed, and we have attempted to achieve that balance by highlighting relative strengths of each platform where appropriate.

Critique 1: The study applies different QC thresholds across platforms (≥20 unique molecular identifiers (UMIs) for CosMx vs. ≥5 for Xenium; Methods, lines 547–557). While platform differences may justify this, the rationale is described only as based on "data visualization" (Supp. Fig. 5). Would the authors consider showing whether CosMx's reported superiority is robust to a uniform QC threshold across platforms? This could clarify how much of the observed advantage reflects filtering choices versus true performance.

R1A1: R1A1: We thank the reviewer for highlighting this point and providing an opportunity to further clarify the rationale used for our QC thresholds. There are no widely recommended or universally adopted QC thresholds, and prior studies have not reported this step in detail, making it difficult to understand how QC thresholds may have influenced comparability across published or pre-print literature. We chose our threshold based on panel size and data distribution/visualization and found a cut-off of 5 for Xenium and 20 for CosMx reasonable. We recognized that different QC threshold choices may influence comparative results, and highlighted this point in the manuscript. In Supplementary Table 4, we provide comparative results with no QC filter applied. When no QC filtering is applied to Xenium or CosMx data, CosMx had better quality in 15 out of 16 samples for multi-tissue data and all four samples for Xenium 5K versus CosMx 6K comparisons. Applying a 20 UMI QC filter to CosMx data resulted in a low number of filtered cells and did not substantially affect the post-QC quality metric magnitudes across samples. On the other hand, applying a 5 UMI QC filter to Xenium data greatly improved its post-QC data quality, and several samples with higher CosMx data quality pre-QC now have higher Xenium data quality post-QC. In this regard, the application of the current QC thresholds biases the comparative performance towards Xenium by removing a higher proportion of low-quality cells relative to CosMx. We also attempted to apply universal QC thresholds to data from both platforms, but the Xenium data became excessively filtered when increasing the threshold beyond 5 UMI. Even when comparing CosMx data with no QC filtering (before QC

in Supplementary Table 4) to post-QC Xenium data with only higher quality cells (after QC in Supplementary Table 4) CosMx still outperforms Xenium in most samples on a per sample basis.

Critique 2: The manuscript reports that CosMx uniquely identifies Treg–mast cell interactions in ileal Crohn's disease (Results, lines 162–200; Fig. 2c–e). FOXP3 is included in the 119 overlapping genes (Fig. 2b), but its upregulation is only detected in CosMx. Similarly, IL-6 signaling is discussed as a key axis (lines 185–200), but it is unclear whether IL6 or IL6R were present or detectable in the Xenium panels. This distinction is not clarified in the Discussion (lines 304–310 or 366–368). Additional detail on whether these genes were present but undetected—or entirely absent—would strengthen claims of platform-specific biological discovery.

R1A2: We are extremely grateful for this comment from the reviewer because it pushed us to delve further into the Xenium probe quality metrics and we feel that it substantially strengthened the manuscript as well as the claims of platform-specific biological discovery. As noted in our Introduction to Resubmission section in this response letter, we have made some key observations in relation to why CosMx but not Xenium identified FOXP3 as a differentially expressed gene for inflamed ileal CD. FOXP3 was observed to have a low Q-score (less than 20) for Xenium in 5 out of the 16 samples used in the multi-tissue comparative analysis. This means the gene was detected in those 5 samples, but the platform assigned a low confidence score in detection and filtered it out for downstream analyses. These 5 samples were inflamed ileal CD (n = 2; samples I0294 ITDF00A and I0275 ITDF00A), inflamed rectum UC (n = 2; samples I0321 RSDF00A and I0286 RSDF00A), and non-inflamed ileal UC (n = 1; sample I0321 ITDF00A). Notably, not all the inflamed samples had low FOXP3 Q-scores for Xenium, and samples I0262 RSDF00A (inflamed rectum CD), I0303 RSDF00A (inflamed rectum CD), I0284 RSDF00A (inflamed rectum UC), and I0277 RSDF00A (inflamed rectum UC) all had Q-scores of 20 or higher for FOXP3. In contrast, most non-inflamed samples had high quality Q-scores for FOXP3. This inconsistency in Xenium probe detection confidence for FOXP3 between inflamed and non-inflamed samples is the source of the discordant DGE results between CosMx and Xenium. We demonstrate this through a sensitivity analysis where we no longer filter out genes with low Q-scores in Xenium (referred to as no QV filter in supplementary materials). When including the full data without any Q-score filtering, we now observe FOXP3 to be one of the differentially expressed genes in Xenium inflamed ileal CD data. This sensitivity analysis confirms that platform specific technical performance variability for Xenium resulted in missed biologically meaningful signals which were identified by CosMx. This also highlights that when using Xenium for cohort studies and clinical trials, researchers should review per-gene performance and detection confidence variance across groups or disease states being compared, and we cannot rely solely on the tissue-level Q-score summary statistics. If there exists systematic artifacts in terms of gene detection confidence variation across disease states, the reliability of downstream analysis results will be greatly affected. Our findings highlight the importance of in-depth data quality review for imaging-based spatial transcriptomics data in clinical research.

Revised Text: (Page 9) As noted in Supplementary Table 2, all Xenium multi-tissue panel runs were of high quality meeting the recommended probe detection confidence threshold (sample-level Q-score > 20). At the per-gene level, 347

out of 377 panel genes also showed Q-scores > 20 across samples, and the 30 genes with lower universal Q-scores were of limited biological relevance to IBD (Supplementary Table 7). Notably, although FOXP3 had a Q-score above 20 aggregating across samples suggesting high probe detection confidence, per-sample examination revealed reduced FOXP3 Q-scores specifically in a subset of inflamed ileal CD and UC samples relative to other sample types. To understand whether this sample-wise variability in probe detection confidence (inflamed versus non-inflamed) influenced cohort-level DGE results, we re-analyzed Xenium data without Q-score filtering and recovered FOXP3 as a significant differentially expressed gene in inflamed ileal CD (Fig. 2c). This demonstrates that Xenium's performance variability for FOXP3 detection across samples, in particular, probe detection confidence decreases in inflamed tissues, was the underlying reason why FOXP3 was detected as a significant differentially expressed gene in inflamed ileal CD by CosMx but not by Xenium with standard Q-score filtering.

Critique 3: The authors performed differential expression analyses using overlapping genes between CosMx and Xenium panels (e.g., 119 genes for the multi-tissue panels, Fig. 2b; 2,552 genes for the 6K/5K comparison, Fig. 3c, line 1020). However, PGK1—though present on both CosMx 6K and Xenium 5K panels—was detected only in CosMx. This discrepancy is noted (lines 221–224), but the Discussion (lines 304–310) primarily attributes platform performance differences to global technical metrics rather than addressing gene-specific detection failures. Could the authors clarify whether the absence of PGK1 detection in Xenium reflects platform sensitivity, expression thresholding, or other technical limitations?

R1A3: We thank the reviewer for this comment which expands on the comment related to FOXP3 in ileal CD. As noted in response R1A2, the ability for CosMx but not Xenium to detect FOXP3 as a differentially expressed gene was observed to be a result of variability in Xenium probe detection confidence on inflamed tissue. We therefore similarly evaluated PGK1 to see if probe detection confidence was a source of this observation in differential gene expression. PGK1 was observed to have high Q-scores (> 20) across all 4 samples separately in Xenium 5K. We observed, however, that the range in Q-scores for PGK1 was wider in inflamed UC (Q-score range 9-40) compared to non-inflamed UC (Q-score range 17-40). This suggested that in addition to sample level variance in probe detection confidence, cell-specific variance for probe detection confidence within each sample might also be a contributing factor for discordant DGE results for Xenium. In exploring the cell type specific Q-scores for PGK1 we observed the lowest Q-scores for inflamed UC samples in T cell sub-sets compared to non-inflamed UC or healthy rectum. T cell specific differential gene expression analyses for Xenium 5K, however, again demonstrated a lack of identification for PGK1 as a differentially expressed gene even after including low quality probe detections. Review of absolute detection for this gene identified that CosMx 6K detected PGK1 2-fold more in inflamed UC samples relative to Xenium 5K (15% of cells in CosMx versus 6-7% of cells in Xenium with or without QC/Q-score filtering). T-cell specific expression of PGK1 in inflamed UC samples was 5% for CosMx and less than 2% for Xenium even after including low Q-score gene detections. This demonstrates that like other genes (CSF3) highlighted in our biological case studies, Xenium 5K had lower sensitivity for detection of PGK1 and this was the primary reason for discordant DGE results between Xenium and CosMx.

Revised Text: (Page 11) *PGK1* (phosphoglycerate kinase 1) was uniquely identified by CosMx, but not Xenium, as a differentially expressed gene in UC. All 4 samples separately demonstrated high average Q-scores (> 30) for *PGK1* across cells in the Xenium 5K panels, however the range of Q-scores for *PGK1* varied substantially within each sample (Supplementary Table 7). A wider range in probe detection confidence was observed among cells for *PGK1* in the inflamed UC samples (Q-score range 9-40) compared to the non-inflamed UC sample (Q score range 17-40). Further review of cell-type specific variance in Q-scores for *PGK1* revealed that CD8+ T cells, Naïve/Memory T cells, and Tregs, had a wider range in *PGK1* Q-scores in inflamed UC samples compared to non-inflamed UC or healthy rectum samples, and the lowest Q-scores for *PGK1* in T cells was seen in inflamed UC samples (Supplementary Table 8). CosMx 6K detected *PGK1* in 2-fold higher T-cells compared to Xenium 5K in inflamed UC (5% vs. 2%) even after including low Q-score gene detections for Xenium. T-cell specific DGE for Xenium 5K did not identify *PGK1* as a significantly differentially expressed gene due to this low overall detection and sensitivity for *PGK1* in inflamed UC.

Critique 4: The authors note that CosMx leverages PanCK/CD45 protein markers for improved cell type annotation (Methods, lines 572–589), and they mention in Supplementary Figure 1 that this approach yielded more consistent annotations than RNA-only methods. However, they do not show whether biological signals—such as Treg signatures—remain robust in CosMx using RNA-only annotations. Would the authors consider providing a control analysis using RNA-only annotation to clarify the extent to which protein markers influence biological interpretations?

Critique 5: Some other concerns stem from the interpretation of Supplementary Figure 1a. There are substantial differences in the cell-type annotation results between platforms. A discussion or interpretation is lacking. Well-matched scRNA-Seq references were used to perform label transfer. Assuming these references are a good approximation to overall tissue composition (albeit underrepresentation of specific cell types in dissociated scRNA-Seq is well established):

- There is a huge difference on the reported proportion of absorptive epithelial cells, with Xenium results much more similar to estimates from the reference data.
- Annotation of CosMx samples report very high proportion of stromal (particularly endothelial) cells as compared to Xenium and dissociated references.
- Overall, at least for well-represented cell types, the observed trend is that Xenium samples correlate better with proportions from scRNA-seq references (with some exceptions).

These observations are hard to interpret without a quantitative assessment of how the various gene panels balance the representation of both major cellular compartments (epithelial/mesenchymal/immune) and more fine-grained cell types. However, it is reasonable to expect that these imbalances affect many of the results and interpretations throughout the paper. This needs to be addressed before any differential analysis or assessment of performance is conducted.

To that end, we would suggest:

- Providing quantitative results on the label transfer analysis (e.g, prediction scores if the Seurat framework is being employed) and an assessment of how the different panels recapitulate unambiguous cell populations. This would set the stage on what is the biology captured by each of the platforms and panels employed. For small panels, a UMAP visualization of the data can also provide some insight into how each panel is able to distinguish cell compartments and types.
- On a related note: Can the authors provide an explanation as to why the cell-type specificity and granularity of unsupervised clustering results (Supplementary figures 6 and 8) seems to be much higher for Xenium, as compared to CosMx?

R1A4-5: We thank the reviewer for these thoughtful comments related to annotation, variability in annotation across platforms, impacts of protein gating strategies, and potential influences on downstream interpretation of results. We have combined Critique 4 and 5 together when responding as they are both touching upon this broader thematic concept of cell type annotation, and we have leveraged the RNA-only annotation suggested by the reviewer in Critique 4 throughout the additional analyses to address Critique 5.

As suggested by the reviewer, we have provided quantitative results on the label transfer analysis in our revised manuscript in Supplementary Table 9 and we have provided this for major and minor cell types. For CosMx, we have further provided this for both the protein-gated strategy which leverages panCK/CD45 as well as RNA alone strategy to understand how our protein-gated strategy influenced confidence in cell type annotation across platforms. In general, Xenium had higher confidence in annotation for major cell types but the protein gating strategy for CosMx did improve confidence in annotation for major cell types. Minor cell types, however, were quite variable for both platforms and both platforms demonstrated lower confidence in annotation of Tregs. We recognize the point made by the reviewer regarding concordance of cellular proportions with reference scRNA-seq datasets to understand which platform may be closer to ground truth and how these confidence scores in annotation should be interpreted, however, for the reasons highlighted by the reviewer that dissociated scRNA-seq has known biases in cellular proportions due to sample prep/cellular viability we feel this is likely not an accurate method for defining accuracy in cellular annotations. This led us instead to perform spatial multi-omics for orthogonal confirmation of biology to ensure we had accurate Treg annotation using protein expression from the same slide/sample which would be the ideal ground truth given it uses the same sample/same cell and protein-based annotation is known to be more accurate than RNA based annotation. Indeed, our spatial multi-omics confirmed biology observed by CosMx for Tregs, despite the low confidence in annotation for Tregs with Seurat annotation metrics on RNA data. This suggests that perhaps alternative approaches are needed to determine confidence in RNA annotation. In the revised manuscript we have added a dedicated section discussion annotation results and multi-omics, with an additional figure 5. We thank the reviewer for this point as we feel it pushed us to generate new data that substantially strengthened the overall value of the manuscript and may help the field begin to consider how best to define annotation confidence for spatial transcriptomics.

Revised Text: (Page 15, Cell type annotation and spatial multi-omics) The overall confidence in RNA-based annotation with Seurat label transfer of major cell types was higher for Xenium (Supplementary Table 9). RNA-based annotation

confidence for minor cell-types, however, was quite variable and lower for Tregs on both platforms across commercial panels (Supplementary Table 9). The use of a protein-gating strategy did improve confidence in annotation for CosMx (Supplementary Table 9), suggesting that multi-omics-based annotation approaches may ultimately prove most ideal. The low overall confidence in annotation of Tregs across platforms warranted further protein-based confirmation of biological observations. CosMx multi-omics (64-plex protein panel + 6K RNA panel) of new inflamed ileal CD samples confirmed spatial co-localization of Tregs with macrophages using spatial proteomic annotations, and cell-cell interactions for pathways observed from our comparative analysis using spatial transcriptomics expression (Fig. 5a). This orthogonal confirmation in new samples provides further confidence in our biological insights derived from the comparative benchmarking and supports the value of multi-omics.

(Pages 15-16, Quantitative spatial multi-omics and transcriptional heterogeneity) When performing spatial multi-omics in clinical research, customization of protein markers will be of tremendous importance so panels can be fit-for-purpose based on biological insights and/or intended use. Ideally, spatial multi-omics could leverage complementary platforms run sequentially on the same slide for optimal data alignment. To test the feasibility of this, we performed CellScape, a multiplex quantitative immunofluorescent panel capable of using off the shelf primary conjugated antibodies, followed by CosMx on the same slide. We were able to successfully align the data and achieve CellScape and CosMx multi-omics with high quality data from both platforms (Fig. 5b-e). These samples allowed for further exploration regarding the relationship between protein expression and transcriptional heterogeneity. As noted in our work, and prior work in the field using dissociated scRNA-seq, there appear to be both anti-inflammatory and pro-inflammatory *FOXP3*$^{+}$ Tregs in IBD. Given the well-established role of FOXP3 as a master regulator of Treg function, we hypothesized that the relative quantitative protein expression of FOXP3 may identify Treg transcriptional sub-types. Indeed, CellScape was able to spatially identify FOXP3^{high} Tregs, FOXP3^{medium} Tregs, and FOXP3^{low} Tregs (Fig. 5d-e). These Treg sub-types were transcriptionally distinct with a higher proportion of FOXP3^{low} Tregs expressing TGFβ3 compared to FOXP3^{medium} Tregs and FOXP3^{high} Tregs (Fig. 5f). TGFβ3 has known pro-inflammatory effects and the combination of IL-23 dependent TGFβ3 production in combination IL-6 induces highly pathogenic Th17 cells compared to TGFβ1 + IL-6 induced Th17 cells in autoimmune disease. This demonstrates the need for multi-omics and the potential ability to use quantitative proteomic multi-omics to build and/or train new annotation models that can be used to not only define cell-types but also potentially define cellular transcriptional heterogeneity.

<u>Critique 6</u>: The authors appropriately address segmentation differences by normalizing transcript data per cell area (Methods, lines 507–509) and apply this in specific comparisons (e.g., Fig. 1c, Fig. 1g, Supp. Table 2). However, it is not always clear whether this normalization was applied consistently across all comparisons, especially in downstream biological findings (e.g., Fig. 2, Fig. 3). A supplemental table presenting both per-cell and per-area results side-by-side could help readers interpret the impact of segmentation differences.

<u>R1A6</u>: We thank the reviewer for this comment. Supplementary Table 5 provides per cell comparisons stratified by whether QC was performed, across full or overlapping panels, and segmentation approaches. This will aid the readers in

understanding how different segmentation approaches and the use of QC thresholds together influence per cell comparisons between platforms. This was done as a sensitivity analysis, and all other analyses are primarily focused on per cell area.

Critique 7: Figure 1b: it should be noted that global markers (EPCAM and PTPRC), are to various degrees downregulated in inflamed samples in both platforms. It needs to be clarified if the specific analytical choices made by the authors limit the scope of their differential expression results. Differential expression was performed at the level of whole tissue/sample, across disease groups, not at the cell-type level. In this respect, important analytical details are missing: Was the data normalized with SCTransform using only the intersection of genes in both panels (~100), or for all genes in the panel ~500 for Xenium, 1,000 for COSMx)?. Variance stabilization might be affected by overall sample signal for small numbers of genes, due to limited information to estimate the mean/variance distribution.

R1A7: As our study was intended to guide cohort studies and use of spatial technology in clinical research, we approached the data analysis in a manner consistent with how it would be used in those studies. Accordingly, the comparisons were done at the tissue level, across disease groups, and each panel was normalized for the entire panel because in clinical research it would be intended that initial comparisons would focus on differences between disease groups or pre-post intervention tissue samples and the entirety of the panel would be used for the platform chosen to maximize discovery potential. We have clarified this in the methods. Our evaluation of overlapping genes from these data provides an opportunity to gauge how current off-the-shelf panels for each might perform in cohort studies relative to each other.

Revised Text: (Page 35, Methods) SCTransform was performed for all genes in each platforms panels separately as this is how the panels would be intended for use in cohort studies and clinical trials where the entirety of the panel is used. We restricted the analysis to genes detected in at least 10% of cells in either compared condition and with at least 0.1 log fold difference between groups, and we present results for overlapping genes between panels to guide interpretation of comparative detection of differential genes between platforms.

Critique 8: Given that the tests are run in whole tissue/sample, the overall expression level and cell-type specificity of the non-intersecting genes (specific to each panel) is likely to impact that of the intersecting genes. So, all these results might be dependent on the specific biases of each panel, not only to platform-specific technical factors as the authors suggest. The effect sizes across different panels can hardly be compared to each other.

- For instance, although the two platforms were used on the same samples, the baseline differences in the panels (non-intersecting genes, a majority) results in an artificial compositional shift between the platforms. Downregulation of EPCAM in inflamed samples is expected due to lower percentages of epithelial cells in inflamed tissues, the downregulation of PTPRC is striking. How significant is the upregulation of genes like VWF which may be linked to a higher annotation rate of endothelial cells?

Issues like this result in uncertain. Regardless, what seems to emerge here is that the differential expression analysis performed by the authors, and its association with cell type abundances, are unrelated with true transcriptional regulation. Unless a clear explanation or re-analysis (e.g. by cell type) is provided, the interpretation of differential expression results should avoid using terms like "up-regulation" and/or restrict the discussion to compositional changes or provide interpretation contingent on the gene composition of each panel.

- An example of these issues is actually apparent in Figure 1b. Even though the figures were truncated in the x axis, Supplementary Table 5 clearly shows that the most downregulated genes in the COSMx inflamed samples are all B-cell and epithelial constitutive genes (IGKC, IGHG1, IGHG2, IGHA1, MALAT1, S100A6, JCHAIN, B2M, PIGR, CD74, HSP90AA1, KRT19, KRT20, NEAT1, EPCAM), likely associated with differential composition/annotation results. For Xenium, the most downregulated genes in inflamed CD are also sets of epithelial (AQP8) and B-cell markers different to those in COSMx, due to different panel representation. Whether this affects differential expression across all other compartments is unclear but likely, similar to classical issues on the interpretation of Bulk RNA-Seq. Therefore, affirmations like "CosMx identified significant up-regulation of T cell markers in inflamed ileal CD and Xenium identified significant downregulation of T cell markers in inflamed ileal CD" are inaccurate and likely due to the differential representation of cell-specific genes across the different panels, not to the inability of any given platform to detect bona-fide transcriptional regulation.
- The same concern applies to the discussion on FOXP3+ and Tregs: differential expression for FOXP3 is not even reported in the supp. tables for the Xenium tests. This is likely due to FOXP3 not passing arbitrary thresholds (genes detected in 1% of cells with effect size >0.1). For bigger panels, the thresholds employed are at least 10% detection, which is a rather stringent threshold and limits the differential expression tests to predominantly constitutively expressed genes. Given that the number of genes in the panels is small and differential expression is done at the sample level, it is unclear why the authors needed to impose those thresholds prior to running the tests.
- Regarding differential expression in UC, the authors link the results on genes like CSF3 and PGK1 to the outperformance of COSMx. Why the authors focused on these genes is unclear. It is likely that PGK1 did not pass the detection threshold in XENIUM. It could also be argued for example that: according to test results in XENIUM, the most upregulated gene in UC is DUOX2 (not shown if Figure 3b, which is truncated), a classical marker of inflammation in UC. This gene is not captured by COSMx, likely because it is not even present in the panels for this platform.

<u>R1A8:</u> We recognize the point highlighted by the reviewer that panel specific variation in composition and tissue/disease specific variance in expression for genes which may or may not be overlapping across panels will have some influence on intersecting gene differential expression results. However, short of making custom panels for both platforms that use the exact same gene panel we feel this is the best way to fairly compare both platforms for use in clinical research given this is how these platforms are currently being used. It could be also viewed that if a platform does not identify known biology of importance (i.e. FOXP3 in IBD) with an off-the-shelf panel then perhaps that panel composition isn't ideal for that

disease. The reviewer highlighted the FOXP3 and PGK1 observations as examples of why panel variance may have influenced comparative differential gene expression results. As noted in our earlier response, when including low probe detection confidence calls for FOXP3 in inflamed UC samples we observe that FOXP3 differential expression is now significant and consistent with results from CosMx using the same panel normalization technique (SCTransform for the entire panel), thresholds (genes detected in 1% of cells with effect size > 0.1), and methodology used originally. We feel this clearly demonstrates that probe detection confidence for Xenium, and not panel normalization techniques, variance in panel composition, or differential gene expression thresholds, are a primary source of discordant results for differential gene expression between platforms. For PGK1 we demonstrate that even after accounting for low probe detection confidence, and T cell specific DGE analyses, the Xenium 5K panel did not identify PGK1 as a significant differentially expressed gene. This was due to the low overall sensitivity in identifying this gene in inflamed UC samples overall and in T-cells specifically. This is consistent with the reduced sensitivity in gene detection for Xenium 5K we observed for other key biological markers (CSF3).

<u>Critique 9</u>: The most distinct advantage of spatial analysis is to provide insights into co-localization and niche composition associated with disease phenotypes. By itself, the results provided are relevant and informative, but due to the ambiguities highlighted above, all the cell-cell communication analyses are strongly influenced by the results of cell-type annotations, which require a more profound analysis of the effect that panel composition has on these analyses. In this respect, it can hardly be addressed which platform may provide a more informative account of changes in cell-cell interactions.

<u>R1A9</u>: We thank the reviewer for this comment and agree that cell-cell interaction, co-localization, and niche composition are valuable advantages of spatial analyses. As noted in the Seurat label transfer prediction scores, both platforms performed comparably in annotation confidence for Tregs despite panel variances in composition. Protein based annotation would be considered ground truth and in this regard, we performed spatial multi-omics with spatial proteomics combined with CosMx spatial transcriptomics. This orthogonal single-cell multi-omics spatial analysis confirmed spatial interactions and cell-cell interactions observed from the RNA-alone spatial analyses we performed in our original analysis for CosMx. We feel this helps to further support the validity of our findings and case studies.

<u>Critique 10:</u> Finally, the authors claim that previous comparative analyses have indicated an outperformance of Xenium as compared to COSMx. The authors emphasize that these studies are in pre-print format, which is not surprising being an emerging field. It should not prevent the authors to provide a more neutral assessment. A recent preprint on FFPE mucosal biopsies, which is also referenced by the authors (Meninillo et al), provides compelling support for the outperformance of the Xenium platform in terms of cell-type annotations (including granulocytes) and niches associated with differential therapy response. This preprint analyzes and discusses the effect of segmentation, downplay its impact to a limited number of cells, and provides distinct biological insights.

R1A10: We thank the reviewer for this comment and have attempted to take a more neutral tone to our interpretation where appropriate while maintaining statements that are now well-supported by the additional analyses requested by the reviewers. We appreciate the reviewer highlighting the comparative study by Meninillo et al. Detailed review of the methodology for that manuscript identified that the authors created a custom panel for Xenium which specifically removed high expressing genes to reduce potential limitations on gene detection by other probes. A similar customization was not done for CosMx where high expressing genes were kept in the panel. This hardly feels like a fair comparison between platforms where genes are pre-selected to optimize performance for one platform but not the other.

Critique 11: Regardless, there are numerous comparative studies both published and in pre-print format that have evaluated the pros and cons of each platform, and multiple examples exist in support of each. Here, the authors aspire to provide recommendations for the adoption of a specific spatial technology in a very specific setting, that is screening FFPE samples in multi-center studies in Inflammatory Bowel Disease. And to this end:

- Assessment of the technical performance of different platforms, particularly related with the effect of cell segmentation on downstream analysis, is highly dependent on the context, implementation and computational methods that are in continuous development. A definite recommendation in this regard is not possible.
- Even using high plex panels, most users of spatial technologies recognize the need for panel customization. In the specific case of IBD, the small plex gene panels provided by COSMX and Xenium (even the colon-specific panels) miss a significant number of disease-relevant and cell-type specific genes. In our experience, even the high plex ~5K panels do not include a good number of relevant genes. Therefore, in terms of biological performance, it is hard to believe that large-scale, consortium-like studies on a specific disease and tissue will rely on manufacturer, generic gene panels, like the ones employed in this work. Given that different disease phenotypes will be evaluated using these approaches (e.g. response to therapy, fibrotic complications, and so on), there is a need to design custom targeted panels. Therefore, a more informative recommendation in the context of IBD will require a detailed evaluation using appropriate panels that better represent the molecular pathways involved in disease pathogenesis. The authors could inform the field by highlighting the genes or pathways particularly lacking in each of the "off the shelf" xenium vs COSMX approaches.

R1A11: We appreciate this point highlighted by the reviewer and do agree that ultimately customization may be needed which is disease and context specific with consideration for the intended use. For example, if being used to study a novel therapeutic intervention then the panel will need to incorporate pathway genes of biological relevance for that drug/target. In our study we do highlight performance variance for each platform with increasing panel size. Given the recent launch of spatial single-cell whole transcriptome for CosMx, and the ability of this platform to scale panel size with limited impact on performance, as compared to the observed performance impact for Xenium when going from colon/multi-tissue panels to 5K, where financially feasible we anticipate the spatial whole transcriptome panel will offer opportunities to overcome customization needs. We have discussed panel customization in the limitations section given we did not

specifically study this and do highlight that Xenium may be ideal for this scenario given the ability to build small custom panels for a discrete number of slides/samples thereby providing cost-effectiveness in spatial validation.

Revised Text: (Page 20, Discussion) The intent of our study was to define the ideal platform for spatial biology discovery, and we therefore cannot comment as to which platform is ideal for small plex or custom experimental designs. In this regard, Xenium may offer some advantages through custom panel creation and small panel designs for discrete experimental questions given the observed performance for its colon specific panel in comparison to larger panels. It is important to note that all 3 platforms have distinct approaches to custom probe design and recent data suggests Xenium panels may have off-target probe binding. Similar evaluations for CosMx were not feasible because the probe sequences are not publicly available, however, our in-vitro validation and multi-omics orthogonal confirmation of biological observations made by CosMx provides some confidence that the superiority in comparisons was not driven by false positive detections. Custom probe development and comparisons across platforms will need to consider this potential risk, and further work is needed for all spatial platforms in this regard.

Critique 12: (Minor) The last sentence in the introduction says: "We anticipate that our work will set the standard for IBD consortiums as well as other gastrointestinal conditions in this evolving space." This may be too bold a statement given how quickly this field is moving including improvements in analysis pipelines.

R1A12: We thank the reviewer for this comment and have removed this sentence.

Critique 13: (Minor) Figure 1c is unreadable and hard to interpret.

R1A13: We thank the reviewer for highlighting the importance of figure readability. We have now enhanced the readability of all the figures by increasing the in-figure text font size and improving figure legends and captions.

Critique 14: (Minor) Double check some of the references that have the wrong number (e.g. the neutrophil reference).

R1A14: We thank the reviewer for the detailed review of references and have double checked them throughout.

Reviewer #2

Spatial transcriptomics provide support in uncovering novel pathways and cell-cell interactions, however, the available data are limited. Here, 66 intestinal biopsies (FFPE) from non-diseased controls and IBD patients of varying disease locations were evaluated using ST platforms (CosMx; Xenium). A spatial atlas of 2.5 million intestinal cells is provided. Quality and quantitative differences were observed between the two platforms, with more advantages for the CosMx platform. The authors state that the study establishes a technical and biological benchmark for ST in IBD. Overall, this is a technical paper analyzing the performance of both systems and other factors which is important for any future studies. The majority of the manuscripts is about the technical details (probably better judged by a person into those techniques). In a second step, they analyzed ileal samples with contradicting results. For me, it is not clear which one is right. One possibility would be to add immunohistochemistry to prove one or the other. Given this revealed contradiction, how do we validate these results? Here, a path (based on the data provided) should be indicated (which will be important for any future analysis. The approach of going into the mouse system and evaluating splenocytes is not convincing when discussing ileal inflammation. Furthermore, the reasoning provided is questionable since anti-IL-6 in clinical trials did not result in an improvement but rather in more complications (Danese S et al. Gut 2019; 68(1):40-48).

<u>R2A1</u>: We thank the reviewer for this comment and in the revised manuscript we have added multi-omic confirmation of cellular proximity and cell type specific spatial interactions which provides added confidence in observations made. Mast cell markers were not available for spatial proteomics panel and therefore we could not include these in the panel, but the ability to verify alternative spatial interactions for macrophages along with our lab-based experimental evidence offer support for our spatial interactions. The in-vitro validation was intended to confirm the effects of specific cellular interactions and co-culture experiments, identified by our spatial cell-cell interaction results. We agree this may not be specific to ileal CD given the experimental design, but the observation related to mast cell interactions with Tregs was not identified in alternative disease locations (inflamed rectum UC or CD). Therefore, the spatial data does help provide location specific context to the potential importance of the biological observations for cellular interactions confirmed in the lab. The IL-6 clinical trial (Danese S. et al. Gut 2019) demonstrated significantly higher rates clinical response and remission with the anti-IL6 therapy versus placebo and met its primary clinical endpoint. This population was refractory to other biologics (anti-TNF therapy) and therefore high-risk for complications. Small bowel complications in Crohns trials have been seen including a warning for JAK inhibitors due to small bowel perforation, but JAK inhibitors are now one of our most widely prescribed medications with tremendous effectiveness in clinical practice. These safety components do not negate the biological value of targeting IL-6, and emerging technologies that limit systemic toxicity through localized delivery mechanisms and/or gut restricted targeting may help overcome limitations with traditional large molecule biologic drug development approaches.

<u>Revised Text:</u> (Page 15) CosMx multi-omics (64-plex protein panel + 6K RNA panel) of new inflamed ileal CD samples confirmed spatial co-localization of Tregs with macrophages using spatial proteomic annotations, and cell-cell interactions for pathways observed from our comparative analysis using spatial transcriptomics expression (Fig. 5a).

Critique 2: The number of cells is derived from different sites and the patients represent a broad spectrum, leading to the question of how many cells from one region to make a statement, how many patients from one subtype to be sure about a finding? What is the necessary quality control? This appears important since only then will these datasets be of use to the community. This has not been addressed adequately. Thus, while significant for the technical advance of the field, the points above should be addressed, only then is this technical paper of value for future analysis.

R2A2: We thank the reviewer for highlighting the need to take into consideration power calculations when considering comparative studies and cohort studies. To that effect we have added a section that outlines power for our study and how evolutions in technology/bioinformatics may further influence power considerations for the application of spatial transcriptions in clinical trials.

Revised Text: (Pages 16-17) An important consideration when implementing any technology into cohort studies or clinical trials is defining the potential impact of sample variance and evolutions in technology on power calculations. Given the rapidity of evolution in instruments and bioinformatics, we evaluated this potential impact by re-running samples on a recently upgraded CosMx platform (CosMx 2.0) to understand how instrument and bioinformatic upgrades may change data quality and how this might influence interpretability of comparative studies or future design of clinical studies. We observed our original comparative analysis of CosMx 1.0 versus Xenium to be sufficiently powered for the comparison of transcript diversity (unique genes), but not transcript counts (Fig. 5g). Comparative studies leveraging CosMx 1.0 would need a minimum of 10 samples for comparison of gene diversity (unique genes detection) and 30 samples for comparison of transcript counts (counts per gene detected) across platforms and all these samples should be from a single disease (e.g., IBD) and tissue type (e.g., gastrointestinal) to be sufficiently powered. Ideally, these samples should include various disease states for the tissue type to account for variability in performance of platforms in inflamed tissue as we have demonstrated. Notably, CosMx 2.0 yielded improved data quality which resulted in substantial improvements in power calculations (Fig. 5h-i). This highlights that when performing studies or comparing across studies, investigators should account for instrument and software versions to avoid introduction of batch effects from technical and/or bioinformatic upgrades.

Reviewer #3

Stated goal of the manuscript is laudable – to compare leading ST platforms in a systematic way. The methodology also seems to be a strength – sequentially cut samples and doing a direct comparison of sequentially cut samples using Xenium vs. CosMx. Weaknesses include: uniformly poorer performance of one platform relative to the other, irrespective of factors that should affect data quality of all ST platforms (e.g. block quality, panel size, fixation time, sectioning timing); poorer data metrics for Xenium (e.g. transcripts per cell) reported by this manuscript compared to previously published studies; and functional experiments that neither validate nor refute observations made in the manuscript.

R3A0: We thank the reviewer for recognizing the main contributions of our paper and the strength of methodology for comparative considerations. We appreciate the points highlighted as potential weaknesses which we have aimed to address during revision.

Critique 1: All data metrics observed in this study were poorer for Xenium compared to CosMx and this poor performance was independent of factors that should affect data quality of all ST platforms, e.g. block quality, panel size, fixation time, sectioning timing. This discrepancy raises the possibility that there may have been systematic issues in data generation and/or analyses in this study.

R3A1: We thank the reviewer for this consideration. We would like to note several points when considering Xenium data quality. First, a 10x Xenium specialist reviewed the data quality after generation to ensure it passed QC recommendations. Second, the samples were run by a 10x certified vendor (NuSeq) which is certified to provide data quality comparable to 10x Genomics running the samples themselves. Third, Q-scores for all the tissue samples were well above the recommended threshold demonstrating overall high confidence in probe detection and this was true for the vast majority of genes on a per gene analysis as well. Fourth, we re-segmented the tissue as recommended by independent scientific publications using best practices which had no influence on comparative performance. Finally, even when applying QC thresholds for Xenium that favor enhanced data metrics and comparing this to CosMx data without QC which results in inclusion of low quality cells, CosMx still outperforms Xenium in our comparative study. We would like to highlight that other comparative studies have leveraged custom panels for Xenium but not CosMx, have not reported data filtering in detail, and they have not certified how CosMx was run and whether expertise was present for the CosMx as the alternative platform. For these reasons we feel our data quality is as ideal as possible for both platforms.

We agree that fixation, block quality, and sectioning, don't appear to be primary determinants of comparative performance. Panel size does indeed have an influence as demonstrated by our Xenium 5K comparative analysis relative to the multi-tissue panels run on the same blocks and magnitude of differences. We have also provided more in-depth exploration of our key biological case studies for FOXP3 and PGK1 which explain that the key reason for performance differences was gene specific variance in probe detection confidence and gene detection sensitivity for Xenium in inflamed tissue specifically relative to non-inflamed tissue which is why this technical performance variance influenced DGE results. In this regard, it does appear that inflamed/diseased tissue state is a major determinant of Xenium data

quality as suggested by the reviewer. Prior studies have leveraged varying tissue locations, disease states, and sample numbers, and may therefore have been underpowered to identify this insight. This is supported by our power calculations provided in the revised manuscript.

Critique 2: The authors report that 32.78% of Xenium cells had low transcript counts (< 5 transcripts per cell) (line 85-87). This seems to be much lower than the transcript counts per cell reported in the published and pre-print literature (which includes ~41 published papers that have used Xenium and ~26 pre-prints that have reported Xenium, from a quick and non-exhaustive Pubmed search). As two examples, (1) Figure 2b from PMID 40082609 (Marco Salas et al, Nat Methods 2025) shows an average of ~500 transcripts per cell and (2) Figure 3e from PMID 38114474 (Janesick et al, Nat Communications 2023) shows a median of 166 transcripts per cell. Though this metric will vary depending on the study and tissue analyzed, this discrepancy again raises the possibility that there may have been systematic problems in data generation and/or analyses in this study.

R3A2: We thank the reviewer for highlighting the other studies in the space. It should be noted that those studies leveraged per cell analyses without reporting on average cell sizes which makes it difficult to compare across studies. They also did not provide detailed information in QC filtering of low-quality cells and they were performed on a wide range of tissue types which are known to have variability in performance, including the use of fresh frozen tissue as opposed to FFPE tissue. For the reasons highlighted in the above response, we do feel our data quality for Xenium is not a concern when making comparative analyses. We have also provided detailed metrics and reporting for results when modifying QC thresholds and/or segmentation approaches which will allow for readers to fully explore how these metrics influenced results.

Critique 3: The authors appropriately cite four pre-prints that aimed to systematically compare Xenium and CosMx, all of which came to a different conclusion than the current study. The authors state that the reason for their discrepant conclusions was a "lack of consideration for cell segmentation variability across platforms, potential impacts of increasing plex size" in the other studies. However, the authors tested these variables here and found that accounting for these variables did not result in improved performance of Xenium. Moreover, the authors state that "Xenium's expanding boundary detection model results in significantly larger cells, which increases the surface area available for transcript assignment" and therefore should result in higher per cell transcript counts for Xenium. However, even when accounting for this variable, they did not see improved performance for Xenium on both a transcript per cell area and transcript per cell basis. Hence, the variables that the authors discuss do not seem to explain the discrepant conclusions of their study and the other four pre-prints (as well as previously published papers).

R3A3: We thank the reviewer for this point and agree that in our original submission we had not provided a clear justification for why Xenium performance variance resulted in differential biology that was identified by CosMx after testing for the points highlighted. In this revised manuscript, with deeper exploration of gene specific probe detection

confidence, we do observe that the lower gene specific probe detection confidence for Xenium in relation to FOXP3 in inflamed tissue was a clear source of comparative differences between platforms. In this regard, we thank the reviewer as the comment pushed us to explore the data in more depth and provide a more justified explanation of our results.

Revised Text: (Pages 18-19) Previous comparative studies have suggested that Xenium may be technically superior to CosMx. These studies have limited sample sizes, along with technical and methodological differences that make it difficult to extrapolate observations to all tissue types and disease states. Notably, they lack a direct comparison of platforms within a clinical cohort study design to understand how performance variability influences biological discovery. After accounting for data quality, filtering, and variations in cell segmentation approaches, we observed that variability in probe detection confidence and sensitivity for Xenium in inflamed tissue was the primary source for discordant results between platforms when performing cohort level analyses. Our observations related to *FOXP3* and *PGK1* highlight that greater attention is needed for gene-level probe detection confidence for Xenium for each sample and cell type, and researchers will need to account for this potential variance across disease states when conducting cohort level studies with this platform. Similar probe detection confidence scores are not available for CosMx, however, the in-vitro and spatial multi-omics validation of observations made by CosMx provide confidence in results and implementation of this technology within clinical research programs. Future work is needed to define the optimal quality control measures for both platforms to deliver robust data analysis results.

Critique 4: The level of IL-17A expression shown in Figure 2E-G is extremely low (0.83 – 3.16%) and it is unclear whether this represents signal or noise. It is also unclear whether a change from 0.83% to 3.16% of Treg cells expressing IL17A is a biologically meaningful difference. It might be more informative to test Treg function, such as suppressive ability.

R3A4: We thank the reviewer for this point. We would like to clarify that the y axis is proportion of IL-17+ CD4+ T cells, not IL-17 expression levels, across the 3 groups. This was done in triplicate, and also the experiment was then repeated again with triplicates which re-confirmed the significant differences between groups. This is highlighted better in the figure legend now.

Revised Figure Legend Text: e, Schematic diagram of the experimental workflow. f, Representative flow cytometry profile of IL-17A^{+} CD4^{+} T cells. g, Dot plots of the frequency of IL-17^{+} CD4^{+} T cells (n = 3 per group, three groups in total). Representative data visualized, with confirmation from two independent experiments. One-way ANOVA with Dunnett's multiple comparisons test was performed.

Critique 5: The data shown in Figure 3g-I do not seem to support the authors' conclusion. The authors first show that PGK1 is differentially expressed in UC vs healthy rectum. The authors then show that PGK1 is upregulated in Th1, Th2, Th17, and iTreg compared to Th0 (Fig. 3f) (statistically significant between Th0 vs Treg because the Treg group has somewhat less variability than Th2 and particularly Th17). In vitro treatment with a PGK1 inhibitor results in increased

MFI of both RORgt in Th17 cells and increased MFI of Foxp3 in iTreg cells, which would imply that increased PGK1 expression in UC leads to both decreased Th17 function and Treg function. It is unclear how the combination of decreased Th7 and decreased Treg function would impact inflammation.

R3A5: We thank the reviewer for their thoughtful observation regarding the possibility that PGK1 expression might represent a feedback response rather than a primary driver of inflammatory signaling. Indeed, our data suggest that PGK1 expression is elevated in the inflamed mucosa of UC patients and upregulated in multiple T helper subsets (Fig. 3f), consistent with a heightened metabolic state associated with chronic immune activation. The in vitro findings (Fig. 3g–i) show that PGK1 inhibition increases the expression of both RORγt and Foxp3 in Th17 and iTreg cells, respectively. We agree that these findings could be interpreted to suggest that elevated PGK1 might act to restrain effector differentiation programs in both lineages, possibly as part of a metabolic negative feedback loop that limits excessive T cell activation under inflammatory conditions. We have added a component to the interpretation to highlight this further.

Revised Text: (Page 12) PGK1 upregulation may represent a compensatory response to inflammatory stress, limiting overactivation of both Th17 and Treg programs. The paradoxical effect on both lineages underscores the complexity of metabolic control in T cell fate and supports the idea of PGK1 as a rheostat rather than a binary switch. Future studies are needed to dissect whether PGK1 influences T cell lineage stability or plasticity under chronic inflammatory settings.

Reviewer #4

The manuscript by Yiming Li et al with the title: "Spatial transcriptomics atlas of inflammatory bowel disease to guide implementation in research consortiums and clinical trials" is a very well written paper, dealing with the comparison of two major spatial transcriptomics platforms for patient samples with inflammatory bowel disease (IBD). They come to the conclusion that the CosMx platform outperforms the Xenium platform, but more importantly the CosMX platform enables biological discoveries that were not identified by Xenium. As the overall manuscript is especially important for the IBD community, the spatial transcriptomics field may also benefit from the results. Some very important aspect of the manuscript includes:

- Block quality and fixation assessment
- Reanalysing Xenium data with better segmentation approaches
- Consecutive analysis of tissue sections with 2 methods
- Confirmation of cell interactions by in vitro experiments and mouse models

R4A0: We thank the reviewer for recognizing the quality of our study and the importance of our observations. We hope the additional in-depth analyses provided and additional data for multi-omics help to further bolster the reviewer's enthusiasm for the work and potential impact on the field.

Critique 1: (Suppl Figure 1) As scRNA-seq is used as a "reference" for cell type abundance, I wonder which platform represents the cell type abundance more closely? It appears there are often stark differences between what scRNA-seq found, and what Xenium or CosMx detected. Which is reflecting the true biology better? For instance, absorptive epithelium ranges from ~5-25%, depending on the technology. What does the corresponding tissue section show? This should be evaluated by a pathologist, and can be compared with IHC, or the CosMx protein-gated approach. And similar with IHC of Xenium section (consecutive or same section). Otherwise, it is not possible to decide which method is more accurate in depicting biology.

R4A1: We thank the reviewer for this point which highlights that we must begin to consider what "ground truth" represents when looking at spatial technology. The non-destructive nature and limited tissue input required for these spatial technologies as compared to traditional dissociative scRNA-seq will result in variation in cellular population prevalence across technologies. To this effect, we have provided spatial proteomic based confirmation of cell type interactions and biology in tissue on the same slides where spatial transcriptomics was done to better guide how the two align. We feel spatial proteomics offers the most accurate estimate of cellular proportions for comparison to spatial RNA based annotation.

Critique 2: (Results) Line 152+: A major impact on the comparison of the two technologies has the filter selection. The simple cut of of eg. 50 transcripts per cell to remove low quality cells seems arbitrary. As I understand, this threshold was selected for the Xenium 5k and CosMx 6k, although the technology for RNA detection is different. To set such a singular

threshold, you would need to show that for instances cells with <50 transcripts using Xenium cannot reliably profile cells, such as epithelium, vessel endothelium or immune cells. Generally, a ground truth, supported by a pathologist and IHC would be essential. Having lower number of signals per cell does not mean a cell is of low quality or wrongly phenotyped. The result of this threshold seems dramatic: in one sample 91% of cells were excluded in Xenium vs 2% only in CosMx (suppl table 3). This could be from too tissue quality, but this could also be from too stringent filtering.

R4A2**:** We thank the reviewer for this point, and we have provided detailed metrics for both platforms when applying no QC filter versus when applying QC filters and different segmentation approaches we have used. (Supplementary Tables 4-5) The choice of QC filter was chosen based on proportional relationship to panel size (50/5,000) and direct visualization of data distribution as noted in Supplementary Figure 5. As noted in this figure for the Xenium 5K versus CosMx 6K panels we looked at how varying thresholds of QC cut-offs would influence comparative performance of platforms. As noted by the reviewer, even at a threshold of 50, some Xenium samples exhibited large numbers of cells being filtered. In Supplementary Figure 5, we see that increasing QC thresholds preferentially benefit Xenium metrics but the per sample comparisons still do not reach superiority relative to CosMx which stable up to a QC filter count of 200 UMI. We do agree that further work is needed to optimally define QC thresholds using various approaches including impact on cellular annotation, however, this is beyond the scope of this manuscript. We have highlighted the need for this in the manuscript revised text.

Revised Text: (Page 20) Along these lines, further work is needed to define optimal approaches to data quality control and filtering with consideration for probe specificity, probe gene detection confidence, and impacts on cellular annotations and cellular interactions. A detailed and biologically driven methodology for data filtering is yet to be developed for uniform application in spatial imaging-based technologies.

Critique 3: A preprint comparing CosMx and Xenium and come to other conclusions https://doi.org/10.1101/2024.12.23.630033). As it is a non peer reviewed preprint, they need still further evaluation to judge the quality of the preprint. Nevertheless, similar to the here mentioned preprint, it would be important to include antibody based ground truth. Then you could evaluate at which threshold setting the technologies fail to detect major cell types.

R4A3: We thank the reviewer for this point and agree that antibody-based ground truth is needed. In this regard, we provide spatial multi-omics data including a platform that uses primary conjugated antibodies (Cellscape). This multi-omics validation of cellular interactions and spatial quantification of cellular proportions helps further strengthen the observations made in our study.

Critique 4: Further stressing the filter setting, all downstream comparisons such as the detection of CSF3+ positive cells is affected (Fig 3). As you state in your manuscript, that Xenium 5k panel and multi-tissue had lower sensitivity than CosMx 6k panel in detecting this important expression marker, I wonder if this is a filter artefact, or biological truth.

R4A4: We thank the reviewer for this point. To address it, we explored gene specific probe detection confidence for Xenium 5K and observed that all samples had high confidence in probe detection. Meaning, whichever probes were detected for CSF3 in the panels was felt to be high confidence and filtering of low confidence genes was not a source of differences. We have included this in-depth analysis in supplementary table 7.

Revised Text: (Pages 10-11) In an inflamed UC sample, the Xenium multi-tissue panel detected 6-fold more cells expressing *CSF3* (colony-stimulating factor 3) than Xenium 5K. However, both Xenium multi-tissue and Xenium 5K had substantially lower *CSF3* expression than CosMx 6K in the same spatial regions (Fig. 3b), despite having high confidence in probe detection across panels (Supplementary Table 7).

Critique 5: Similar holds true for the detection of FoxP3+ Tregs co-localization with mast cells and T cell marker up regulation detected by CosMx, but downregulation in Xenium. Does it hold true if the filter settings are changed?

R4A5: As noted in our overall response and detailed response to Reviewer 1, the differential detection of FOXP3 for CosMx and Xenium was due to low gene specific probe detection confidence for Xenium primarily in inflamed tissue. Upon removing the Q-score filter recommended by 10x, the FOXP3 gene does emerge as a differentially expressed gene, but it becomes difficult to ignore this filter because it is recommended by the company for removing low confidence probe detection and would introduce tremendous noise. Notably, this lower gene specific probe detection confidence for FOXP3 with Xenium was seen most predominately in inflamed IBD tissue highlighting the tissue specific variation for the platform and its influence on biological discovery/insights.

Critique 6: (Results) Line 100: Instead of re-segmentation, investigating nuclear transcripts rather than all transcripts, would circumvent segmentation issues, as suggested in https://doi.org/10.1038/s41592-025-02617-2 . This could be interesting to assess.

R4A6: We thank the reviewer for this point and agree that assessment of nuclear versus non-nuclear transcripts would be of interest. We have added this detailed assessment in supplementary table 7 and note that CosMx remains superior to Xenium even when limited to per cell nuclear detection.

Critique 7: (Results) Line 279: The change to an alternative reference atlas which contains neutrophils makes sense. Discussing the aspect of reference atlases, irrespective from CosMx or Xenium would be important to include.

R4A7: We thank the reviewer for highlighting the need to consider the reference atlas for annotation. We have added a new annotation section and also added a specific sentence highlighting the need to be mindful of the reference atlas.

Revised Text: (Page 14) This re-analysis with an alternative single-cell RNA sequencing reference atlas highlights the importance of defining optimal strategies for annotation to guide accurate biological discovery.

(Page 15, Cell type annotation and spatial multi-omics) The overall confidence in RNA-based annotation with Seurat label transfer of major cell types was higher for Xenium (Supplementary Table 9). RNA-based annotation confidence for minor cell-types, however, was quite variable and lower for Tregs on both platforms across commercial panels (Supplementary Table 9). The use of a protein-gating strategy did improve confidence in annotation for CosMx (Supplementary Table 9), suggesting that multi-omics-based annotation approaches may ultimately prove most ideal. The low overall confidence in annotation of Tregs across platforms warranted further protein-based confirmation of biological observations. CosMx multi-omics (64-plex protein panel + 6K RNA panel) of new inflamed ileal CD samples confirmed spatial co-localization of Tregs with macrophages using spatial proteomic annotations, and cell-cell interactions for pathways observed from our comparative analysis using spatial transcriptomics expression (Fig. 5a). This orthogonal confirmation in new samples provides further confidence in our biological insights derived from the comparative benchmarking and supports the value of multi-omics.

(Pages 15-16, Quantitative spatial multi-omics and transcriptional heterogeneity) When performing spatial multi-omics in clinical research, customization of protein markers will be of tremendous importance so panels can be fit-for-purpose based on biological insights and/or intended use. Ideally, spatial multi-omics could leverage complementary platforms run sequentially on the same slide for optimal data alignment. To test the feasibility of this, we performed CellScape, a multiplex quantitative immunofluorescent panel capable of using off the shelf primary conjugated antibodies, followed by CosMx on the same slide. We were able to successfully align the data and achieve CellScape and CosMx multi-omics with high quality data from both platforms (Fig. 5b-e). These samples allowed for further exploration regarding the relationship between protein expression and transcriptional heterogeneity. As noted in our work, and prior work in the field using dissociated scRNA-seq, there appear to be both anti-inflammatory and pro-inflammatory *FOXP3*$^{+}$ Tregs in IBD. Given the well-established role of FOXP3 as a master regulator of Treg function, we hypothesized that the relative quantitative protein expression of FOXP3 may identify Treg transcriptional sub-types. Indeed, CellScape was able to spatially identify FOXP3^{high} Tregs, FOXP3^{medium} Tregs, and FOXP3^{low} Tregs (Fig. 5d-e). These Treg sub-types were transcriptionally distinct with a higher proportion of FOXP3^{low} Tregs expressing TGFβ3 compared to FOXP3^{medium} Tregs and FOXP3^{high} Tregs (Fig. 5f). TGFβ3 has known pro-inflammatory effects and the combination of IL-23 dependent TGFβ3 production in combination IL-6 induces highly pathogenic Th17 cells compared to TGFβ1 + IL-6 induced Th17 cells in autoimmune disease. This demonstrates the need for multi-omics and the potential ability to use quantitative proteomic multi-omics to build and/or train new annotation models that can be used to not only define cell-types but also potentially define cellular transcriptional heterogeneity.

Critique 8: Line 366+: For spatial biology discovery, potentially whole transcriptome platforms would be more beneficial due their untargeted nature.

R4A8: We thank the reviewer for this point and do agree whole transcriptome platforms may be more beneficial in their untargeted nature. We have not added a discussion to this effect in the manuscript because we have not yet fully evaluated CosMx WTX versus 6K, and would not want to prematurely suggest enhanced benefit without evaluating impacts to sensitivity with the panel size increase.

Critique 9: Time and costs of the technologies would be interesting to add to the discussion.

R4A9: We thank the reviewer for pointing out this out and have added several sentences throughout on cost/time as a factor, particularly when considering customization.

Revised Text: (Page 19) Taken together, CosMx offers the best balance of feasibility for implementation, consistency in data quality, and detection efficiency, which makes it ideal for use in large multi-center collaborations and clinical trial programs for IBD given the cost and time intensive nature of these experiments.

Critique 10: The list in Supplementary Table 1 is incomplete.

R4A10: We apologize for the confusion we may have created in our original submission on this table. This is only the overlapping genes between platforms, not the full panels for each platform.

Critique 11: Supplemental table 5 + 6: The p values are on the next pages, and it is not possible to identify the corresponding gene.

R4A11: We thank the reviewer for pointing this out. The DGE analyses now have the p value and adjusted p value on the same sheet as the gene information for CosMx and Xenium separately.

Reviewer #5

The authors systematically compared two spatial transcriptomics platforms using intestinal biopsies from IBD patients and healthy controls. They provided benchmarking of the data to guide the integration of spatial transcriptomics in multicenter studies. They also validated platform-specific findings through targeted in vitro experiments. Overall, this is a valuable comparative study with direct relevance for the IBD research community and broader spatial biology field.

R5A0: We thank the reviewer for recognizing the main contributions of our paper, and the potential value it holds for the larger spatial biology field.

Critique 1: The authors used Baysor for re-segmentation, but did not sufficiently clarify how differences in segmentation directly affect biological outcomes or interpretations. How did you ensure that the performance difference between CosMx and Xenium was not significantly influenced by cell segmentation methods, and how this influenced the downstream biological interpretation.

R5A1: We thank the reviewer for this point, and it touches upon identifying why the two platforms identified differential biology and providing a clear and demonstratable reason for how technical performance differences influenced differential biology discovery/interpretation. In this regard, we highlight the FOXP3 and PGK1 observations and how probe detection confidence, particularly in inflamed tissue, was a primary determinant of differential downstream biological interpretation between platforms. As noted in response to reviewer 4, we also explored nuclear versus non-nuclear detection at a per gene/per sample level. The nuclear detection would be unaffected by variation in cell segmentation and even here we can see the differences between platforms.

Critique 2: Could you clarify why platform-specific QC thresholds (20 UMIs for CosMx, 5 UMIs for Xenium) were chosen, and whether a unified threshold would influence the comparative conclusions? Did the authors test a standardized method for a more unbiased comparison?

R5A2: We thank the reviewer for this point, and we have addressed this in our overall response to reviewers as several reviewers raised this point.

Critique 3: Can you explain why key genes (e.g., FOXP3, PGK1) show differential expression patterns specifically between platforms? Could this reflect differences in probe chemistry or hybridization efficiency? There is a significant drop in Xenium platform performance with increasing panel size and critical differences observed between platforms regarding negative probe counts and RNA quality sensitivity, the authors need to test different normalization and QC methods across platforms to ensure the accuracy of the downstream biological interpretation.

R5A3: We thank the reviewer for this point which is consistent with other reviewers as well. We have worked to explain the variance in detection of FOXP3 and PGK1 and differentially expressed genes between platforms, but we are unable to accurately comment on why Xenium 5K had lower sensitivity. It was not due to probe detection confidence as the per gene Q scores were quite good on the 5K panel. Notably, the Xenium 5K and CosMx 6K were standardized for QC thresholds/methods and therefore the results provided are using a standard approach for both platforms. We can only assume that the reduced sensitivity of Xenium 5K is due to amplification, change in chemistry, and/or optimal limitations but this is an assumption that is difficult to test, and we have noted this in the manuscript.

Critique 4: Could the authors provide more details on the number of independent biological replicates and the reproducibility of their in vitro experiments validating key genes (FOXP3, PGK1)? Did variability exist across replicates?

R5A4: We thank the reviewer for this point. In the main figure, each dot for each bar plot represents a replicate and the centricity of the dots helps define the within experiment variation. For the second experiment where this was repeated to ensure consistency, we have provided those results below for the reviewer.

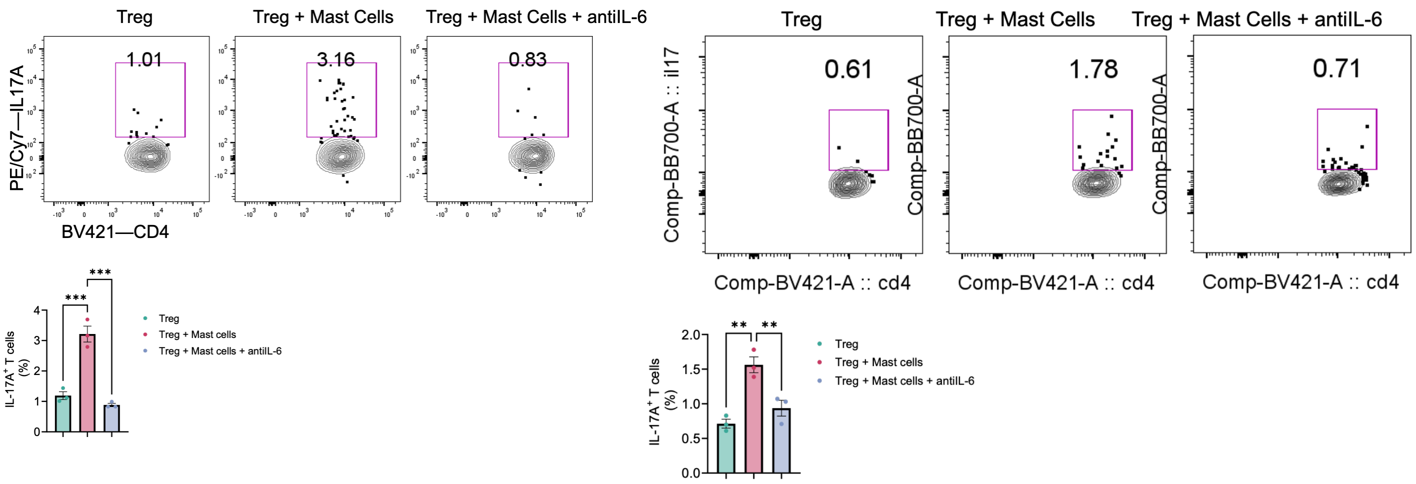

Critique 5: In spatial cell-cell interaction analyses, did the authors assess how robust and reproducible these interactions were across multiple samples, particularly in biopsies from heterogeneous disease states?

R5A5: This is a very interesting and thoughtful comment which we don't believe the field has fully considered yet for its potential impact on results. The CCF Plexus Biorepository, an independent cohort of samples, offers some validation of key biological/cell-cell interactions as being consistent which we feel helps support the reproducibility component. With regards to understanding how inter-sample variability in biology (cellular proportions, cellular proximity, disease states, treatment exposures, etc) influence accuracy in cell-cell interactions – we are underpowered to make those conclusions or assessments, but we have added this to the manuscript as a point within the power calculation work we added. We are looking forward to working on this concept as our datasets grow from the consortiums and thank you for the very thoughtful insight.

Revised Text: (Page 17) Further work is needed to understand how within patient and pre-post intervention variance may further influence power calculations to accurately guide sample size estimates in clinical research. This is particularly relevant when considering the accuracy of cell-cell interactions after accounting for cellular proportion variance, cellular proximity variance due to sectioning variance, disease states, treatment exposures, and patient characteristics, and future work is needed to define these influences on precision for cell-cell interactions in power calculations.

Critique 6: Some figure legends (particularly Figures 2 and 3) are overly dense or lack clarity on replicate numbers and statistical tests.

R5A6: We thank the reviewer for this point and have worked to make figures more readable with clarity in manuscripts on replicates and statistical testing.

Critique 7: Currently, limitations are discussed in broad terms without explicitly addressing technical biases or specific limitations of spatial transcriptomics technologies.

R5A7: We have substantially expanded the limitations section and agree that a more balanced and detailed discussion was needed.

Revised Text: (Page 20) Although our study was conducted with rigor, it does have certain limitations. We did not evaluate the other available imaging based single-cell spatial transcriptomics platform (MERSCOPE) because the plex size is limited compared to CosMx and Xenium. The intent of our study was to define the ideal platform for spatial biology discovery, and we therefore cannot comment as to which platform is ideal for small plex or custom experimental designs. In this regard, Xenium may offer some advantages through custom panel creation and small panel designs for discrete experimental questions given the observed performance for its colon specific panel in comparison to larger panels. It is important to note that all 3 platforms have distinct approaches to custom probe design and recent data suggests Xenium panels may have off-target probe binding. Similar evaluations for CosMx were not feasible because the probe sequences are not publicly available, however, our in-vitro validation and multi-omics orthogonal confirmation of biological observations made by CosMx provides some confidence that the superiority in comparisons was not driven by false positive detections. Custom probe development and comparisons across platforms will need to consider this potential risk, and further work is needed for all spatial platforms in this regard. Along these lines, further work is needed to define optimal approaches to data quality control and filtering with consideration for probe specificity, probe gene detection confidence, and impacts on cellular annotations and cellular interactions. A detailed and biologically driven methodology for data filtering is yet to be developed for uniform application in spatial imaging-based technologies.

Critique 8: Abbreviations and terminology (e.g., "multi-tissue" vs. "MT", "segmentation" vs. "seg.") sometimes vary across figures and text.

R5A8: We thank the reviewer for this point and have worked to harmonize terminology throughout.

Critique 9: The authors could try to clearly define axes, labels, and legends with concise text, increasing font size and readability of the figures.

R5A9: We thank the reviewer for this point and have worked to make the figures more readable throughout.

<u>NCOMMS-25-36839 – Response to the Reviewers</u>

We would like to thank the reviewers for the thoughtful critiques and continued support in optimizing the quality, value, and balance of our work and its presentation. We have worked to address the remaining critiques and look forward to the remainder of the review process.

Reviewer #1: In response to this and other reviewers' concerns, the authors now provide:
* Additional assessment and discussion on how QC metrics can affect the evaluation of Xenium performance in the samples analyzed
* A small multi-omic spatial dataset on the COSMx/CellScape platforms.

Although the authors are leaders in a field that is not yet crowded, these technologies are rapidly evolving. It seems unlikely that one platform is significantly better than the others - instead, the best platform will depend on the question being asked. Unfortunately, I am not satisfied with the author's responses to most of my major concerns.

<u>R1A0:</u> We thank the reviewer for the thoughtful critiques provided previously during our original submission and during our revision. We also appreciate the reviewer highlighting the additional work which includes:

- Additional QC metric-based analyses which confirmed consistency in results across all QC thresholds
- Additional QV metric-based analyses which confirmed that differential gene results for FOXP3 were due to low confidence scores with Xenium in inflamed UC samples, and not due to panel composition, our normalization approach, whole-tissue DEG analysis, or other bioinformatic considerations
- Additional CosMx data generation with newer software/instrument version
- Detailed data on annotation confidence scores across platforms
- Additional multi-omics datasets for 2 spatial proteomic platforms combined with CosMx RNA profiling, and a highlighted discussion on the importance of protein-based annotation
- Power calculations for comparative studies
- Extensive revisions throughout to better capture points highlighted by reviewers

<u>Critique 1</u>*: The authors continue to leverage observations on a few genes to overstate the performance of the COSMx platform compared to Xenium in clinical applications. The authors refer to these hand-picked examples rather than providing a direct and convincing response to most of my original questions.*

<u>R1A1</u>: We thank the reviewer for the detailed critiques during the original submission and our revision. We would like to clarify that our comparative analysis was done for all overlapping genes, not a select subset of genes, and we appreciate the reviewer previously stating that this approach was "a well-executed and timely study that addresses a critical need for platform validation in translational IBD research" which was done with rigor as the reviewer previously noted "We commend the authors for their rigorous approach and clear

relevance to ongoing consortia efforts. We particularly appreciate investigating some of the biology of the mast cell-T Reg potential interaction." We highlighted several genes as case studies including CSF3, PGK1, and FOXP3, and they represent three distinct and biologically relevant scenarios through which platform sensitivity and probe confidence influenced inference. We have revised the manuscript to further emphasize that these genes are illustrative case studies, rather than sole basis for our conclusions, and to more clearly frame our findings around global sensitivity and robustness patterns.

As requested by the reviewer in the prior revision we further analyzed the FOXP3 DEG disparity between platforms which was done to address the critique from the reviewer regarding "Additional detail on whether these genes were present but undetected—or entirely absent—would strengthen claims of platform-specific biological discovery." FOXP3 emerged as one of several sentinel genes illustrating inflammation-dependent probe confidence variability that materially altered cohort-level differential expression results, highlighted by CosMx and Xenium identifying T cell markers to be differentially expressed but in opposite directions. This substantial discordance in identifying T cell biology was what drove the decision to explore Tregs as case studies throughout the manuscript. This identified that the FOXP3 disparity between platforms was due to the low detection confidence of Xenium for this gene in inflamed UC and hence, as the reviewer noted, has strengthened the claims of platform-specific biological discovery. This has substantial importance for readers and the field as no prior comparative study to our knowledge has investigated in detail how Xenium probe detection confidence influences comparative performance or biological discovery performance.

<u>Critique 2</u>: *In terms of technical performance, I am still surprised that the Xenium platform is reported to perform so poorly in this manuscript. Most of the authors' assessments are based on diversity and detection, yet it is unclear how these metrics are normalized. I provide more specific examples below. Here, although the authors cite at least 7 studies where Xenium shows superior performance, they argue that those evaluations are not relevant due to limited sample size and other factors like technical differences and biological insights. In my opinion, this is a biased and arbitrary assessment of the literature. The results herein contradict those from leading experts in spatial transcriptomics (e.g., https://www.biorxiv.org/content/10.1101/2023.12.13.571385v1.full.pdf; please note that I am not related in any way to this reference), who have reported that the COSMx platform has a concerningly low dynamic range, low signal/noise ratio and inflates the signal of low abundance genes. This last factor alone could explain a significant proportion of the differences observed by the authors between the two platforms (e.g., in diversity).*

<u>R1A2:</u> We thank the reviewer for highlighting the disparity in the literature on performance of platforms and providing a reference to a preprint manuscript for consideration. We agree that prior studies, including those reporting superior performance for Xenium, are valuable and informative within their respective experimental contexts. Our intent is not to dismiss these studies, but to highlight that platform performance is strongly dependent on tissue state, RNA integrity, panel size, and analytical filtering assumptions and in this regard we

have focused our discussion on highlighting what we feel are key strengths of our study which includes the technical consistency in how samples were prepared, the expertise of the groups preparing samples, the extensive evaluation of how block quality and slide preparation may influence data quality, the large and diverse sample size focused on a single disease, and exhaustive evaluation of how data quality influences interpretation particularly in relation to Q-scores. For these reasons, we have framed our work to be specific to guiding IBD research and are not making any claims that CosMx is in general superior to Xenium for all tissue types or scenarios which is consistent with the reviewers point that these comparisons are "highly dependent on the context, implementation and computational methods that are in continuous development." We do not know whether blocks used in prior studies were pre-screened for DV200 which would have resulted in better Xenium data quality or how CosMx was prepared/run which may have influenced CosMx data quality, and clearly there are tissue and disease specific variations for platform performance. For these reasons, we have refrained from extensive comparisons between the comparative studies and we limited our discussion of those studies to highlight key differences in ours, and framed our results as being focused on how our study may shape use of these platforms for IBD research.

Like other comparative studies, our study leverages comparative performance for transcript counts and unique genes detected, but we do also report on background 'noise' through negative probes, differential gene expression analysis differences, differences in annotation confidence, impacts of probe detection confidence, impact of increasing panel size, and impacts of various factors on comparative performance such as DV200, QuBit, sectioning, preparation, and features important when integrating this technology into clinical research. For these reasons we believe our approach complements prior studies by emphasizing cohort-level biological inference under clinically relevant conditions. Our conclusions are not limited to solely differences in diversity and detection, and as noted by the reviewer previously, "a well-executed and timely study" which was done with rigor.

<u>Critique 3</u>: *Finally, the authors repeatedly refer to the relevance of the results presented here in the context of setting the standard for applying ST technologies in the NIDDK and in other IBD consortia. This manuscript constitutes a significant technical effort. However, the choice of metrics to evaluate performance is narrow and contradicts many previous studies, and it is difficult to draw conclusions about depth, generalizability, and biological relevance from a few hand-picked examples. In my opinion, the results presented here don't constitute an unbiased assessment, nor do they prove that one platform displays superior performance that would justify its promotion as the best choice for large-scale efforts.*

<u>R1A3:</u> We thank the reviewer for the feedback and guidance provided throughout the revision process. As noted by this reviewer previously, and we also agree, that our study is well executed and has "clear relevance to ongoing consortia efforts." We have further revised the manuscript to clarify that our findings are not intended to establish a universally 'best' platform, but rather to inform platform selection for large-scale IBD

cohort studies where tissue inflammation, variable RNA quality, and operational feasibility are central considerations. We have also tempered language regarding standards-setting and now frame our study as providing a disease- and context-specific benchmark rather than a prescriptive recommendation. We also note the comments by Reviewers 3, 4, and 5 in response to our revisions, who stated: “I think that the authors have made an effort not to make overly definitive statements given frequent changes in technological and computational advances in this rapidly evolving field. I also think that they have tried their best to address the concerns of reviewers” (Reviewer 3), “the authors have thoroughly addressed the concerns regarding quality control thresholds and filtering steps” (Reviewer 4), and “the authors have substantially addressed the major concerns raised in the initial review, and the revised manuscript represents a clear improvement in methodological rigor and interpretive balance” (Reviewer 5).

<u>Critique 4</u>*: Authors' response R1A4-5: The authors’ response to all my original concerns is limited to highlighting their Foxp3/Tregs findings, ignoring other important cell types or compartments. However, some of the concerning features of their COSMx cell composition results (overestimation of endothelial cells, underestimation of absorptive epithelium) cannot be justified by merely referring to unexplained, potential biases of dissociated scRNA-seq studies. The authors do not address my concerns that the supplementary heatmaps highlight the superior specificity of cell-type calls in Xenium compared to COSMx.*

<u>R1A4:</u> We thank the reviewer for highlighting the important differences between platforms in annotation confidence or cell type calls, which we also acknowledged in our manuscript: (prior revised text are provided here: “The overall confidence in RNA-based annotation with Seurat label transfer of major cell types was higher for Xenium.”) We agree that RNA-based annotation confidence is an important practical consideration for users. However, we respectfully note that higher label-transfer prediction scores reflect algorithmic confidence relative to a reference, rather than validated biological accuracy. In the absence of protein-based ground truth on the same cells, we have therefore reported annotation confidence descriptively rather than as a definitive performance endpoint. We have highlighted this in our previous revisions under the section “Cell type annotation and spatial multi-omics” and other reviewers have also acknowledged the revisions made throughout the manuscript to note that further and more detailed work, which is beyond the scope of this manuscript, are needed to understand optimal approaches for annotation and influence of data quality on annotation accuracy.

<u>Critique 5</u>*: Authors' response R1A7: I strongly disagree. As a user of ST platforms, I don't think that cohort and clinical studies that apply ST technology will be conducted using whole-tissue differential expression. The major advantage of ST data is to provide spatial context for cell-cell interactions and to validate relevant cell states, both of which require high specificity at the single-cell level. From this perspective, the authors’ results instead demonstrate that the Xenium platform exhibits better overall performance across all cell compartments.*

R1A5: We thank the reviewer for this important perspective and for helping us understand how other users intend to leverage the platforms. In practice, many biological and clinical questions are formulated at the tissue level (e.g., tissue condition, disease state, treatment group assignment, adjustment for placebo cohorts). ST enables more interpretable DGE analysis than bulk RNA-seq and scRNA-seq by allowing cell grouping by spatial compartments and spatial validation of DGE results derived from the same sample. Tissue level DGE analyses are routinely used in current ST studies, particularly in clinical research. Since our aim is to evaluate platform performance across different analytical scenarios, DGE-related evaluation is important, as different applications may prioritize different performance characteristics. In the prior revision, as requested by the reviewer, we did also complete cell type specific DEGs which were consistent with our overall DEG analysis results. As the PIs for two IBD clinical trial NIDDK U01s (Dulai and Lewis), the Crohn's and Colitis Foundation Biorepository (Lewis) which is a multi-industry/academic/patient advocacy partnership, and multiple phase 2 and 3 industry trial translational programs for IBD (Dulai), we do intend to leverage whole-tissue differential expression in addition to cell-type specific differential expression and spatial analyses as we have done in this study. As noted in our response to other critiques, we cannot state that any platform has demonstrated superiority in cell type annotation without a ground truth protein-based annotation of the same cells.

Critique 6: A careful examination of the Supplementary Data provided by the authors (there is no visualization or summarization of these results) reveals that the reliability of cell annotations is superior in Xenium compared to COSMx. This improvement in performance is quite substantial and is consistent across the board and for all combinations of QC metrics. Notably, even Treg annotation is more reliable in Xenium compared to COSMx. The annotation of Tregs is a critical element of any single-cell genomics experiment, and on in its own, would drive many users to favor Xenium technology.

R1A6: We thank the reviewer for this note. We would like to highlight that Seurat label transfer prediction scores do not equate to reliability or accuracy in annotation. As noted in the Seurat label transfer tutorials and methodology, this prediction score "quantifies how confident the algorithm is in assigning a cell type from a reference to a query cell, often ranging from 0 to 1." This does not constitute how close a measure is to the true value, but instead constitutes how close repeated measurements are to each other. Reliability in cell annotation would require a ground truth reference (protein-based annotation). Treg confidence scores for both platforms are below what Seurat defines as ideal (>0.5), and this further validates that protein-based annotation is needed in spatial biology and the added value of multi-omics data in our study. It is also worth highlighting that Xenium had, as noted by the reviewer, numerically higher Treg annotation prediction scores than CosMx even though Xenium filtered out FOXP3 predominately in inflamed UC samples. FOXP3 is the classic Treg marker most often used in the field for annotation of Tregs, so it's unclear how Xenium achieved these annotation prediction scores for Tregs after filtering out the key gene used to define a Treg and what exactly it was labeling as Tregs in Xenium data.

<u>Critique 7:</u> Authors' response R1A8: Again, the authors' response to all my original concerns is limited to highlighting their Foxp3/Tregs findings. My concerns about how whole-tissue differential expression was assessed and interpreted remain.

<u>R1A7:</u> We thank the reviewer for highlighting this point. The reviewer had previously highlighted that panel composition and/or cellular composition/annotation may have influenced why CosMx identified FOXP3 as a significant DEG but Xenium did not which is why we used this case study to help address this concern. The choice to highlight FOXP3 was further supported by the fact that Xenium identified T cell markers to be differentially expressed in the opposite direction as CosMx and such start differences were worth exploring as case studies. In response to this critique and others in the prior revision, we also conducted cell-type specific DEGs which confirmed the results of the whole-tissue DEG, and subsequently identified that the FOXP3 detection confidence predominately in inflamed UC samples was the primary source of discordance DEG results. The cell type DEG takes into consideration panel composition, tissue cellular composition, and it would have identified whether entire panel-based analyses influenced the results of cell type specific marker DEGs.

<u>Critique 8</u>: The authors should make an additional effort to provide a comprehensive interpretation of their results, instead of focusing on just a couple of hand-picked genes. Using their logic, one could argue that Xenium is better because DUOX2 and HK2 are highly relevant genes in IBD that are only detected (and found differential) by this platform. There are numerous examples that I could refer to which raise questions, including the finding (via COSMx) of highly significant upregulation of several X chromosome genes (SPANXB1, SSX4) in inflamed samples, though the relevance and/or relationship of these genes in intestinal samples remains unclear.

<u>R1A8</u>: We thank the reviewer for this point and would like to again highlight that our conclusions are not based on results from a small sub-set of genes. We used an extensive array of analyses to conclude that we will be implementing CosMx as our spatial platform in our NIDDK programs, CCF Biorepository, and industry translational programs for IBD. These include: comparative performance for transcript counts, gene detection, background noise (negative probes), consistency in performance across tissue types (inflamed/non-inflamed), block quality (DV200, QuBit), feasibility for using larger panels without substantial reductions in performance, and feasibility for implementation including implementation with multi-omic approaches. We do recognize and understand the reviewers' points that the DEGs have discordance, and we have added text to this effect in the discussion. T cell DEGs were however the most striking as they were identified to be up-regulated by one platform and down-regulated by another platform in the same comparisons, which is why we focused on FOXP3 and Tregs as case studies throughout. In-depth exploration of every DEG from both platforms is beyond the scope of this manuscript and in this regard the reviewer does further highlight the value of our

study and that the “accompanying spatial atlas serves as a foundational resource for benchmarking, annotation, and hypothesis generation in IBD research and establishes a framework that can be extended to emerging multi-organ spatial datasets.” as noted in the final paragraph of the discussion.

Revised Text (Limitations section of discussion): In this regard, Xenium did identify differentially expressed genes which were not captured by CosMx and Xenium may offer some advantages through custom panel creation and small panel designs for discrete experimental questions given the observed performance for its colon specific panel in comparison to larger panels. It will be important to ensure the custom panel genes have high confidence in detection across tissue types

Critique 9: I don't understand the argument that the percent of cells expressing CSF3 is informative. Which cells express CSF3 in their samples? The authors seem to value this metric given the importance of granulocytes for NIDDK IBD consortiums (although their reasoning is unclear). However, we already know that CSF3 is expressed by inflammatory fibroblasts and other cell types in UC tissues (https://www.nature.com/articles/s41467-023-40156-6, https://pubmed.ncbi.nlm.nih.gov/36720220/). Without a more in-depth assessment, the interpretation of the value of this metric seems superficial. If the percent of cells expressing a gene is a meaningful metric, the authors could highlight in the text that their supplementary bar plots show that the Xenium samples have a higher percent of cells expressing CD14, CD2, CD3E, CD79A, IRF8, MKI67, KLRB1, etc.

R1A9: The cellular source of CSF3 was primarily in neutrophils for the inflamed UC samples, and in our prior revisions we added text on the importance of CSF3R+ neutrophils in the context of our other observations. Prior translational work that led to the funding of our U01 for hyperbaric oxygen therapy in ulcerative colitis identified neutrophils as a key cellular mediator of treatment effect (PMCID 9117812), and prior scRNA-seq based analyses by our group identified neutrophils as key mediators of IBD related dysplasia (PMCID: 10378177; 10977994) making this of relevance to the other U01 on dysplasia screening which will also be using CosMx for spatial analyses. We did not use percent of cells expressing a gene as a meaningful metric when comparing platforms, but as we note in the results this is a case study to highlight the importance of gene sensitivity.

Critique 10: Authors' response R1A9: To address this concern, the authors briefly refer to the addition of new proteomic data, which is not satisfactory. The new data, as presented in the manuscript (Figure 5, snapshots of a few hand-picked tissue sections showing the genes they have previously selected), is not a rigorous validation of the RNA analysis. My comments regarding the superior performance of Xenium in cell annotation, and therefore in any niche analysis, again apply here.

R1A10: We thank the reviewer for this comment. As we noted earlier, we cannot claim superior performance for any platform in cell annotation without a protein-based ground truth. Detailed validation of annotation accuracy across both platforms is not feasible in this study due to the limited current ability for performing multi-omics with Xenium. We have added this to the limitations section.

Revised Text: further work is needed to define optimal approaches to data quality control and filtering with consideration for probe specificity, probe gene detection confidence, and impacts on cellular annotations and cellular interactions, ideally leveraging protein-based annotation as ground truth through multi-omics.

Critique 11: Authors' response R1A10: Regarding the evaluation by Meninillo et al., it should be noted that the authors' results are in agreement: Xenium exhibits superior performance in cell annotation, which is a critical element of ST analysis.

R1A11: We thank the reviewer for highlighting the pre-print by Meninillo and we again note that we do not feel it is feasible or scientifically justified to make claims regarding superiority in annotation without ground-truth annotation of cell types on the same slide using protein-based annotation.

Critique 12: There are multiple references throughout the paper about the ability of COSMx to identify pathways of interest, but no pathway analysis was performed (that I could find), and there was no mention of pathway analysis in the methodology. Other genes (e.g., OSM) are only found in supplementary tables and aren't significantly ranked in the overall results. This pattern indicates some arbitrariness in the authors' interpretation of their results, leading to qualitative, unsupported remarks. I recommend that the authors refrain from upgrading some circumstantial evidence to the level of significant biological findings.

R1A12: We thank the reviewer for this point. We used CellChat for cell-cell interaction analysis, and CellChat is pathway-based since it leverages a curated database of known ligand-receptor interactions organized into biologically defined signaling pathways. We have now revised the Methods section to better clarify this. We do agree with the reviewer that we should refrain from upgrading circumstantial evidence to the level of significance biological findings and have therefore tempered statements in this regard.

Revised Text:

- (Methods) To investigate the interactions among different cell types, we performed pathway-based spatial cell-cell interaction analyses on the CosMx multi-tissue data of ileal inflamed CD samples (in-house: I0294 ITDF00A and I0275 ITDF00A; CCF: 7771, 772A, and 720A) and rectal inflamed UC samples (in-house: I0321 RSDF00A, I0286 RSDF00A, I0284 RSDF00A, and I0277 RSDF00A; CCF: 877A and 3774) using CellChat. CellChat infers spatially proximal cell-cell interactions between cell

groups on the signaling pathway level based on spatial transcriptomics data by summarizing the communication probabilities of all ligand-receptor interactions associated with each signaling pathway, enabling insights into localized cell interactions specific to the inflamed environment. To characterize the roles of different cell types in different cell-cell interaction networks, we also calculated network centrality metrics including weighted out degrees (sender role), weighted in degrees (receiver role), flow betweenness score (mediator role), and information centrality score (influencer role) using CellChat. In our cell-cell interaction analyses, pathway-level results were used as the primary unit of interpretation, while individual ligand or receptor genes were examined in the context of their contribution to broader signaling pathways rather than as standalone findings. This framework reduces arbitrariness in interpretation and ensures that the analytical results are supported by existing literature.

- Having established technical reproducibility, we next qualitatively evaluated whether biological signals detected in our benchmark dataset were consistently recapitulated in the centrally processed IBD Plexus program samples.
- Pathway analyses for cell-cell interactions from the CCF IBD Plexus program inflamed ileal CD samples yielded some qualitative overlap to pathways of known biological importance in CD identified in our cell-cell interaction analyses (i.e. *GALECTIN, CCL, TNF*).
- We identified *FOXP3*$^{+}$ Tregs, macrophages, and monocytes as central contributors to the *IL-16* signaling pathway cell-cell interaction network in the inflamed ileal CD samples from the CCF IBD Plexus program which may have been enabled by the larger number of cells present in this dataset
- Pathway analyses for cell-cell interactions from the CCF IBD Plexus program inflamed UC samples similarly yielded some qualitative overlap for multiple pathways identified in our data (i.e. *OSM, VEGF, SPP1, CXCL, ICAM*).

Critique 13: "Spatial cell-cell interaction analyses of inflamed ileal CD in our CosMx data observed multiple interactions between FOXP3+ Tregs and other immune cells, including monocytes, macrophages, and mast cells, which were not seen in inflamed rectum CD or inflamed UC samples." What do the authors mean by spatial cell-cell interaction analysis? Where and how is this quantified? The authors later refer to "visual inspection"—if this is the case, please refrain from using the word "analysis" unless a quantitative analysis is performed and the methods are properly reported.

R1A13: We thank the reviewer for this point. In the methods we outline the use of CellChat and as noted above, we have tempered many of our statements around this with revisions to avoid overly declarative statements related spatial observations.

Critique 14: Spatial proteomics, Figure 5: How are high/low Treg levels defined? What are the error bars in Figure 5g? How significant is the change of ~15% to ~25% of putative Tregs subsets expressing TGFB3, in the authors' view? How was TGFB3 selected over other genes for this analysis?

R1A14: The methods section notes that the cut-offs for Tregs with CellScape were defined using the Otsu algorithm (text in methods: Marker expression features were extracted at the cell level, and automatic thresholding was performed using the Otsu algorithm. Both two-level (negative/positive) and three-level (negative/low/high) thresholds were computed independently for each tissue region.). We have revised the figure legend to clarify why TGFB3 was chosen, given its correlation with FOXP3 protein expression at the single-cell resolution. Figure 5g is proportions and in the figure legend we have outlined exact number of cells with expression of TGFB3 RNA based on FOXP3 protein expression of low, medium, or high. We have better clarified this in the figure legend now and appreciate the reviewer identifying this point. In our view, the ability to identify a ~10% absolute difference in expression for TGFB3 between Treg subsets that all express FOXP3 at some level is quite meaningful. Traditionally, all these cells would have been grouped into Tregs given they had some FOXP3 protein expression, and this would have skewed comparative analyses and spatial analyses considerably. To be able to provide further fine-grain details on transcriptional heterogeneity linked to relative differences in protein expression for a marker of known biological significance for Tregs (i.e. FOXP3 protein expression) is quite interesting and could guide the future on how we integrate spatial proteomics by benchmarking thresholds for protein expression based on transcriptional heterogeneity.

Revised Text: (Figure legend) TGFB3 RNA was found to be correlated with FOXP3 protein expression at the single-cell resolution. Bar plot showing the proportion of TGFB3 RNA positive cells among $CD4^{+}$ $FOXP3^{low}$, $CD4^{+}$ $FOXP3^{medium}$, and $CD4^{+}$ $FOXP3^{high}$ T cell groups (n = 189 $FOXP3^{high}$ cells, 14.3% positive for TGFB3; n = 310 $FOXP3^{medium}$ cells, 22.9% for TGFB3; n = 427 $FOXP3^{low}$ cells, 28.3% positive for TGFB3; n = 4 FFPE blocks, 8 biopsies).

Critique 15: In the same section, the authors claim that "CosMx multi-omics (64-plex protein panel + 6K RNA panel) of new inflamed ileal CD samples confirmed spatial co-localization of Tregs with macrophages using spatial proteomic annotations, and cell-cell interactions for pathways observed from our comparative analysis using spatial transcriptomics expression. (Fig. 5a)". Where is the quantitative analysis that supports this claim? Where is the cell-cell interaction and pathway analysis shown or described? Is the co-localization confirmation based exclusively on the two snapshots of a handful of cells shown in Figure 5e/5f?

R1A15: We thank the reviewer for this point and have toned down the text to avoid overly assertive claims. Instead, we have highlighted the evaluation performed, the demonstration of spatial co-localization, and exploration of pathways observed in our comparative analysis. We have removed comments regarding validation or quantitative analysis type statements for the points highlighted.

<u>Revised Text</u>: CosMx multi-omics (64-plex protein panel + 6K RNA panel) of new inflamed ileal CD samples demonstrated spatial co-localization of Tregs with macrophages using spatial proteomic marker-based annotations. We explored cell-cell interactions observed from our comparative analyses in this multi-omics dataset, and observed consistency using spatial transcriptomics expression. (**Fig. 5a**) This orthogonal assessment in new samples provides further confidence in our biological insights derived from the comparative benchmarking and supports the value of multi-omics.

<u>Critique 16:</u> Unless I am misinterpreting the power calculations, the calculations shown to support the clinical application of ST platforms are based on the power to address transcript diversity. This choice needs to be justified, as it is my opinion that any power assessment in the context of genomics studies should be focused on the power to detect biologically/clinically meaningful variables, not transcript/gene diversity. In transcriptomics, for instance, one must estimate the number of replicates necessary to detect differentially expressed genes at a given false discovery rate. Instead, the power estimates provided here focus on transcript diversity, which could be misleading for readers that are concerned about well-powered transcriptomics studies.

<u>R1A16:</u> We thank the reviewer for this point and note that the power calculation was done to understand if our study was well powered, and we do outline that these power calculations are for transcript diversity and counts. We agree that ultimately, power analyses will need to consider power in detection of biology, but that would require pre-post intervention samples in a large clinical trial dataset to understand what the minimum number of samples and replicates were that could have produced the same results and how to use variance in data and biology temporally to power future studies in that condition. We have taken note of this suggestion and thank the reviewer as we will look to study this further when CosMx is implemented into the NIDDK programs and consortia efforts. We have also noted this more clearly in the results section to avoid confusion for the reader.

<u>Revised Text</u>: Ideally, future power calculations will focus on minimum replicates and samples needed to identify biology and clinically meaningful variables.

Reviewer #2

No further comments

<u>R2A0</u>: We sincerely appreciate the reviewers feedback and are glad that our revisions adequately addressed prior critiques. The feedback and critiques provided helped strengthen the clinical interpretation and relevance of our work.

Reviewer #3

<u>Critique 1:</u> *My original Critique 4: "The level of IL-17A expression shown in Figure 2E-G is extremely low (0.83 – 3.16%) and it is unclear whether this represents signal or noise. It is also unclear whether a change from 0.83% to 3.16% of Treg cells expressing IL17A is a biologically meaningful difference. It might be more informative to test Treg function, such as suppressive ability."*

Reviewer response "R3A4: We thank the reviewer for this point. We would like to clarify that the y axis is proportion of IL-17+ CD4+ T cells, not IL-17 expression levels, across the 3 groups. This was done in triplicate, and also the experiment was then repeated again with triplicates which re-confirmed the significant differences between groups. This is highlighted better in the figure legend now."

This critique was not adequately addressed. The point is that the proportion of CD4+ T cells expressing IL17A is quite low (0.83-3.16%) and it is unclear whether a chance from 0.83% to 3.15% of Treg cells expressing IL17A is a biologically meaningful difference. I am not questioning whether the difference is statistically significant. I am questioning whether the difference is biologically meaningful. This is why I suggested testing Treg function instead.

However, this point can be addressed by revising the sentences in lines 234-239 to the following: "Tregs co-cultured with mast cells exhibited a statistically significantly higher level of IL-17A (3.16%) compared to Tregs cultured alone (1.01%). In addition, treatment of anti-IL-6 significantly reduced IL-17A levels in Tregs during co-culture with mast cells (0.83% vs. 3.16%, Fig. 2e), indicating a possible critical role of the IL-6 produced by mast cells. Additional functional experiments are warranted to confirm the biological importance of this finding. Recent clinical trial evidence…"

<u>R3A1:</u> We thank the reviewer for this detailed explanation and apologize for not adequately addressing it previously. We have modified the sentence structure as suggested, verbatim.

<u>Critique 2:</u> *I'm not convinced that this manuscript has provided any new biologically meaningful discoveries, but perhaps an attempt to compare leading ST platforms in a systematic way is sufficient for publication. Moreover, I strongly agree with Reviewer 1's critique 11: "Assessment of the technical performance of different platforms, particularly related with the effect of cell segmentation on downstream analysis, is highly dependent on the context, implementation and computational methods that are in continuous development. A definite recommendation in this regard is not possible." However, I think that the authors have made an effort not to make overly definitive statements given frequent changes in technological and computational advances in this rapidly evolving field. I also think that they have tried their best to address the concerns of reviewers.*

R3A2: We thank the reviewer for this point and appreciate the acknowledgement that we have made an effort to not make overly definite statements and we have made our best effort to address concerns of reviewers.

Reviewer #4

Overall, the authors have thoroughly addressed the concerns regarding quality control thresholds and filtering steps. They added several supplementary tables (4 and 5), revised the main figures, and included new data. Importantly, when appropriate quality parameters, such as standard Q-score filtering are applied, the authors now observe comparable biological insights from both the CosMx and Xenium platforms. This results in similar findings between the two platforms, particularly with respect to the role of FOXP3. Some statements and headlines seem overstated, which are pointed out below together with additional comments.

R4A0: We sincerely thank the reviewer for the time, effort, and thoughtfulness of the critiques and feedback. The revision process has substantially improved the quality of the work, and we appreciate your acknowledgement of the added work and effort. We also appreciate your help in ensuring we do not overstate any findings, and have worked to address those below with the other critiques.

Critique 1: (Suppl Figure 1) As scRNA-seq is used as a "reference" for cell type abundance, I wonder which platform represents the cell type abundance more closely? It appears there are often stark differences between what scRNA-seq found, and what Xenium or CosMx detected. Which is reflecting the true biology better? For instance, absorptive epithelium ranges from ~5-25%, depending on the technology. What does the corresponding tissue section show? This should be evaluated by a pathologist, and can be compared with IHC, or the CosMx protein-gated approach. And similar with IHC of Xenium section (consecutive or same section). Otherwise, it is not possible to decide which method is more accurate in depicting biology.
R4A1: We thank the reviewer for this point which highlights that we must begin to consider what "ground truth" represents when looking at spatial technology. The non-destructive nature and limited tissue input required for these spatial technologies as compared to traditional dissociative scRNA-seq will result in variation in cellular population prevalence across technologies. To this effect, we have provided spatial proteomic based confirmation of cell type interactions and biology in tissue on the same slides where spatial transcriptomics was done to better guide how the two align. We feel spatial proteomics offers the most accurate estimate of cellular proportions for comparison to spatial RNA based annotation.
Comment by Reviewer 4:
I fully agree that the ground truth need to be further discussed in spatial technologies, but not necessarily in this manuscript. I agree that spatial proteomics can offer the most accurate estimate of cellular proportions compared to RNA based annotation. The authors added figure 5, which now compares one of the platform (GeoMx) with antibody based staining's.

R4A1: We thank the reviewer for this point and for your support in highlighting that a tremendous amount of work is still needed to guide annotation of spatial RNA based platforms, and the importance of needing ground-truth in spatial technologies which we have attempted to highlight in our multi-omics section.

Critique 2: (Results) Line 152+: A major impact on the comparison of the two technologies has the filter selection. The simple cut of of eg. 50 transcripts per cell to remove low quality cells seems arbitrary. As I understand, this threshold was selected for the Xenium 5k and CosMx 6k, although the technology for RNA detection is different. To set such a singular threshold, you would need to show that for instances cells with <50 transcripts using Xenium cannot reliably profile cells, such as epithelium, vessel endothelium or immune cells. Generally, a ground truth, supported by a pathologist and IHC would be essential. Having lower number of signals per cell does not mean a cell is of low quality or wrongly phenotyped. The result of this threshold seems dramatic: in one sample 91% of cells were excluded in Xenium vs 2% only in CosMx (suppl table 3). This could be from too tissue quality, but this could also be from too stringent filtering.

R4A2: We thank the reviewer for this point, and we have provided detailed metrics for both platforms when applying no QC filter versus when applying QC filters and different segmentation approaches we have used. (Supplementary Tables 4-5) The choice of QC filter was chosen based on proportional relationship to panel size (50/5,000) and direct visualization of data distribution as noted in Supplementary Figure 5. As noted in this figure for the Xenium 5K versus CosMx 6K panels we looked at how varying thresholds of QC cut-offs would influence comparative performance of platforms. As noted by the reviewer, even at a threshold of 50, some Xenium samples exhibited large numbers of cells being filtered. In Supplementary Figure 5, we see that increasing QC thresholds preferentially benefit Xenium metrics but the per sample comparisons still do not reach superiority relative to CosMx which stable up to a QC filter count of 200 UMI. We do agree that further work is needed to optimally define QC thresholds using various approaches including impact on cellular annotation, however, this is beyond the scope of this manuscript. We have highlighted the need for this in the manuscript revised text.

Revised Text: (Page 20) Along these lines, further work is needed to define optimal approaches to data quality control and filtering with consideration for probe specificity, probe gene detection confidence, and impacts on cellular annotations and cellular interactions. A detailed and biologically driven methodology for data filtering is yet to be developed for uniform application in spatial imaging-based technologies.

Comment by Reviewer 4:

The authors have addressed the critique 2 and added now data in supplementary figure 5, as well as in supplementary tables 4 and 5. The revised text is appropriate.

R4A2: We thank you for the time and effort in providing this critique and are grateful that the revisions addressed this adequately.

Critique 3: A preprint comparing CosMx and Xenium and come to other conclusions https://doi.org/10.1101/2024.12.23.630033). As it is a non peer reviewed preprint, they need still further evaluation to judge the quality of the preprint. Nevertheless, similar to the here mentioned preprint, it would be important to include antibody based ground truth. Then you could evaluate at which threshold setting the technologies fail to detect major cell types.

R4A3: We thank the reviewer for this point and agree that antibody-based ground truth is needed. In this regard, we provide spatial multi-omics data including a platform that uses primary conjugated antibodies (Cellscape). This multiomics validation of cellular interactions and spatial quantification of cellular proportions helps further strengthen the observations made in our study.

Comment by Reviewer 4:

The authors included Cellscape proteomics data, which is reassuring that the RNA spatial data is comparative to protein staining. Nevertheless, Fig 5 a is a bit unclear. The box in the upper panel shows single cells by proteomics. The lower panel highlights the RNA expression, such as FOXP3 etc: Two questions: What is the green stain in the lower panel? It looks like several stained cells. Second question: In the upper panel, is a single dot a single transcript? Please clarify.

Although the antibody staining is now included, the authors did not show at which threshold setting the GeoMx technology is still able to detect major cell types, or when it start to fail. For Xenium, such data is not included.

R4A3: We apologize for the lack of clarity in Figure 5. For panel A, there are two parts (upper and lower) and we have revised the text to better reflect that when we said ‘upper panel’ we meant upper half of the panel A and similarly for lower panel/lower half. We have also revised the figure legend to clarify that each dot in the lower half of the panel represents a single transcript detection. In the lower panels (panels E and F) the green stain is CD4. We are showing multiple CD4+ T cells, but only some of the express FOXP3 which is red, and we show the different expression level of FOXP3 visually by staining. We also apologize for any confusion we may have created in our response, but we have not used GeoMx in this revised manuscript. The protein staining is CosMx and as such does not have traditional threshold settings that need to be established like GeoMx. We unfortunately do not have multi-omics data for Xenium as our core facility stated they were not able to run multi-omics on Xenium at the time of our original analysis or revisions.

Revised Text: **a,** Upper half of panel A shows immunofluorescence protein markers (CD3/CD4, green; FOXP3, yellow; CD163, blue; CD68, red) with CosMx multi-omics (64-plex spatial proteomic panel and 6K spatial transcriptomic panel aligned to the single-cell spatial resolution for an inflamed ileal CD sample). Lower half of panel A highlights the colocalization of a pro-inflammatory T-reg (defined by spatial proteomics with CD3/CD4/FOXP3 showing RNA expression for FOXP3/TGFB1/RORC) with a Macrophage (defined by spatial proteomics with CD163/CD68) with expression of TNFRSF4 (OX40) a key pathway interaction identified by comparative analyses. Each dot represents a single transcript detection by CosMx.

Critique 4: Further stressing the filter setting, all downstream comparisons such as the detection of CSF3+ positive cells is affected (Fig 3). As you state in your manuscript, that Xenium 5k panel and multi-tissue had lower sensitivity than CosMx 6k panel in detecting this important expression marker, I wonder if this is a filter artefact, or biological truth.

R4A4: We thank the reviewer for this point. To address it, we explored gene specific probe detection confidence for Xenium 5K and observed that all samples had high confidence in probe detection. Meaning, whichever probes were detected for CSF3 in the panels was felt to be high confidence and filtering of low confidence genes was not a source of differences. We have included this in-depth analysis in supplementary table 7.

Revised Text: (Pages 10-11) In an inflamed UC sample, the Xenium multi-tissue panel detected 6-fold more cells expressing CSF3 (colony-stimulating factor 3) than Xenium 5K. However, both Xenium multi-tissue and Xenium 5K had substantially lower CSF3 expression than CosMx 6K in the same spatial regions (Fig. 3b), despite having high confidence in probe detection across panels (Supplementary Table 7).

Comment by Reviewer 4:

CSF3 has also often lower Q-scores (<20) in supplementary table 7. From my understanding, the standard Xenium pipeline would now remove this transcript due too low quality. This would lead to a similar situation as with FOXP3. Then it would be necessary to evaluated what will happen if the Q-Score filtering is adapted as you described in the revised manuscript starting line 202.

In line 243 the statement "CosMx uniquely identifies Treg biology in ulcerative colitis" seems overstated. As the important UC pathology marker CSF3 is detectable with both platforms GeoMx and Xenium, although potentially with less sensitivity.

Similar: The headline at line 182: "CosMx uniquely identifies Treg-Mast cell interaction in ileal Crohn's disease" needs to be adjusted, as this is Q-score filtering issue in the Xenium platform, as discussed by the authors

R4A4: We thank the reviewer for these comments. We agree that the low Q-scores for CSF3 resulted in filtering of this transcript due to low confidence in detection. When adapting the Q-score filtering as we did for FOXP3, we do indeed identify more CSF3 in the same spatial regions as we did for CosMx. We have added this statement in the manuscript now to reflect the continued importance and impact of Q-scores for Xenium. It should be noted that the Q-score for CSF3 in the Xenium 5K was quite good and high. Therefore, the drop off in detection with the higher panel was not due to Q-score filtering, but was truly due to loss in detection sensitivity.

Revised Text: However, both Xenium multi-tissue and Xenium 5K had substantially lower *CSF3* expression than CosMx 6K in the same spatial regions (**Fig. 3b**), despite the Xenium 5K panel having high confidence (Q-score) in probe detection (**Supplementary Table 7**). Similar to FOXP3, when removing the Q-score filter we were able to identify more *CSF3* expression in Xenium data in the same spatial regions as CosMx.

<u>Critique 5</u>: Similar holds true for the detection of FoxP3+ Tregs co-localization with mast cells and T cell marker up regulation detected by CosMx, but downregulation in Xenium. Does it hold true if the filter settings are changed?

R4A5: As noted in our overall response and detailed response to Reviewer 1, the differential detection of FOXP3 for CosMx and Xenium was due to low gene specific probe detection confidence for Xenium primarily in inflamed tissue. Upon removing the Q-score filter recommended by 10x, the FOXP3 gene does emerge as a differentially expressed gene, but it becomes difficult to ignore this filter because it is recommended by the company for removing low confidence probe detection and would introduce tremendous noise. Notably, this lower gene specific probe detection confidence for FOXP3 with Xenium was seen most predominately in inflamed IBD tissue highlighting the tissue specific variation for the platform and its influence on biological discovery/insights.

Comment by Reviewer 4:

This critique 5 is already well addressed to reviewer 1 and added in the revised manuscript.

<u>R4A5:</u> We thank the reviewer for noting that we have addressed this in detail for reviewer 1 and thank you again for the detailed and thoughtful critique.

<u>Critique 6</u>: (Results) Line 100: Instead of re-segmentation, investigating nuclear transcripts rather than all transcripts, would circumvent segmentation issues, as suggested in <u>https://doi.org/10.1038/s41592-025-02617-2</u> . This could be interesting to assess.

R4A6: We thank the reviewer for this point and agree that assessment of nuclear versus non-nuclear transcripts would be of interest. We have added this detailed assessment in supplementary table 7 and note that CosMx remains superior to Xenium even when limited to per cell nuclear detection.

Comment by Reviewer 4:

Additional quantitative data was added by the authors. But does this for instance improve the detection of highly relevant genes like FOXP3, CSF3 and others?

<u>R4A6:</u> The nuclear versus non-nuclear analysis did not impact detection of key genes highlighted (FOXP3 and CSF3) between platforms. We were not able to find any patterns in the data for specific genes that were most impacted by the nuclear versus non-nuclear detection.

<u>Critique 7</u>: (Results) Line 279: The change to an alternative reference atlas which contains neutrophils makes sense. Discussing the aspect of reference atlases, irrespective from CosMx or Xenium would be important to include.

R4A7: We thank the reviewer for highlighting the need to consider the reference atlas for annotation. We have

added a new annotation section and also added a specific sentence highlighting the need to be mindful of the reference atlas.
Revised Text: (Page 14) This re-analysis with an alternative single-cell RNA sequencing reference atlas highlights the importance of defining optimal strategies for annotation to guide accurate biological discovery.
Comment by Reviewer 4:
Critique 7 is well addressed

R4A7: Thank you for this point as it further highlights the importance of annotation in spatial data and need for work in this space.

Critique 8: Line 366+: For spatial biology discovery, potentially whole transcriptome platforms would be more beneficial due their untargeted nature.
R4A8: We thank the reviewer for this point and do agree whole transcriptome platforms may be more beneficial in their untargeted nature. We have not added a discussion to this effect in the manuscript because we have not yet fully evaluated CosMx WTX versus 6K, and would not want to prematurely suggest enhanced benefit without evaluating impacts to sensitivity with the panel size increase.
Comment by Reviewer 4:
Agree, manuscript is also already a complex topic.

R4A8: Thank you for your comment and for helping us identify other areas of exploration for future work.

Critique 9: Time and costs of the technologies would be interesting to add to the discussion.
R4A9: We thank the reviewer for pointing out this out and have added several sentences throughout on cost/time as a factor, particularly when considering customization.
Revised Text: (Page 19) Taken together, CosMx offers the best balance of feasibility for implementation, consistency in data quality, and detection efficiency, which makes it ideal for use in large multi-center collaborations and clinical trial programs for IBD given the cost and time intensive nature of these experiments.
Comment by Reviewer 4:
As a reader I do not see the costs or time invested in each technology to acquire and analyze the data. Simply stating that spatial technologies are time intensive by nature, is not very informative. It remains still interesting to know the time needed for each technology.

R4A9: We thank the reviewer for this note, and have added text to try to highlight this further. Both platforms take days to prep and run, and cost several thousand dollars per slide. Exact costs are hard to specify in the manuscript given each lab, core, program, may have some variability in pricing based on region, volume, and agreements. Exact timelines for each platform are also hard to comment on in the manuscript given it is

dependent on sample prep speed, efficiency, amount of tissue placed on the slide, and panel size. We have nonetheless attempted to quantify this as best as possible.

Revised Text: The extensive time needed for each run, often 2-3 days of prep and up to 2 weeks of instrument time, and the extensive cost in the several thousands of dollars per slide associated with these experiments, makes it important to maximize value in clinical research.

Critique 10: The list in Supplementary Table 1 is incomplete.
R4A10: We apologize for the confusion we may have created in our original submission on this table. This is only the overlapping genes between platforms, not the full panels for each platform.
Comment by Reviewer 4:
Critique 10 is well addressed

R4A10: Thank you and we appreciate the detailed review and helping to identify this gap originally.

Critique 11: Supplemental table 5 + 6: The p values are on the next pages, and it is not possible to identify the corresponding gene.
R4A11: We thank the reviewer for pointing this out. The DGE analyses now have the p value and adjusted p value on the same sheet as the gene information for CosMx and Xenium separately.
Comment by Reviewer 4:
Critique 11 is well addressed

R4A11: Thank you and we appreciate the reviewers efforts to make the data more readable and interpretable.

Reviewer #5
The authors have substantially addressed the major concerns raised in the initial review, and the revised manuscript represents a clear improvement in methodological rigor and interpretive balance. The added analyses examining results with and without QC filtering, together with run- and gene-level Q-score assessments, convincingly demonstrate that several previously noted inter-platform discrepancies reflect technical sensitivity rather than fundamental biological disagreement, including the clarified case of FOXP3. The inclusion of quantitative label-transfer confidence metrics and orthogonal spatial proteomic/multi-omic validation strengthens confidence in cell-type annotation, while the added power considerations and discussion of platform version effects appropriately temper cohort-level claims. One remaining minor suggestion is to further ensure that the Abstract and concluding statements consistently emphasize robustness trends and methodological insight rather than platform equivalence or broad generalizability. Overall, the substantive reviewer concerns have been adequately addressed, and the remaining issues are largely editorial.

R5A0: We thank the reviewer for this thoughtful review, response, and all the original critiques provided. We have worked to revise the abstract to emphasize the robustness trends as suggested by stating that CosMx ‘tended’ to achieve better quality.

Revised Text: Using over 100 intestinal tissue sections from non-diseased controls and patients with ulcerative colitis and Crohn’s disease across multiple IBD consortia, we generated a spatial atlas of over three million cells and systematically evaluated two imaging-based ST platforms. We observed that CosMx tended to achieve higher detection efficiency than Xenium across commercially available panels in both ulcerative colitis and Crohn’s disease. Xenium performance was negatively impacted by tissue type, block quality, and panel size. CosMx tended to have more consistent detection efficiency across inflamed samples resulting in the identification of Treg-associated biology in both IBD subtypes, which was validated by *in vitro* experiments and multi-plex spatial multi-omics. CosMx’s data quality was unaffected by fixation time or sectioning, supporting its operational feasibility for multi-center studies. This study establishes a technical and biological benchmark for the application of single-cell-resolved ST in gastrointestinal tissue, enabling more reliable application of spatial technologies in translational in IBD research.

Reviewer #1

The authors have done a thorough job of responding to our critiques. The data and analytic strategies presented will inform the field (at least for the time being) in terms of using spatial transcriptomics to better understand IBD pathogenesis and the effect of interventions at a more granular level.

R1A0: We thank the reviewer for the positive evaluation of our revisions and for recognizing the potential value of the spatial atlas and analytic framework presented in this manuscript. We appreciate the reviewer's constructive feedback throughout the review process.

Reviewer #3

The theme of the comments from all 5 reviewers is that this manuscript tends to overstate claims and exaggerate the broad generalizability of their findings. The 5 reviewers all suggested that the authors thoroughly revise their manuscript emphasize robustness trends and methodological insight rather than make broad conclusions about universal platform superiority vs. inferiority and the generalizability of their findings.

The authors have appropriately made revisions, but should review their manuscript carefully and continue to revise the manuscript, particularly the Discussion section, where they continue to overstate their conclusions.

R3A0: We thank the reviewer for the additional suggestion to further review the Discussion section for statements that may overgeneralize our findings. Following this comment, we have now revised the Discussion section to more clearly frame the results as context-dependent benchmarks intended to guide future inflammatory bowel disease research rather than broad claims about universal platform superiority. The edits are highlighted in the revised manuscript.

Reviewer #4

Overall, the authors have responded well to all my comments raised. They have reduced the overstatement regarding the superiority of one platform over the other, by focusing on their IBD cohort and addressing filtering issues. The manuscript is now more balanced.

R4A0: We thank the reviewer for their constructive feedback throughout the review process. We appreciate the reviewer's guidance in helping us clarify the scope of our conclusions.

Reviewer #5

The revisions have addressed the major concerns raised in the initial review.

R5A0: We thank the reviewer for their careful review and for confirming that the revisions addressed their concerns.
